# Synthesis and Aggregation of Amphiphilic Porphyrin‐Perylenebisimide Dyads

**DOI:** 10.1002/chem.202500279

**Published:** 2025-05-02

**Authors:** Erik J. Schulze, Mingjian Wu, Christian D. Methfessel, Erdmann Spiecker, Andreas Hirsch

**Affiliations:** ^1^ Department of Chemistry & Pharmacy Chair of Organic Chemistry II Friedrich‐Alexander‐Universität Erlangen‐Nürnberg Nikolaus‐Fiebiger‐Straße 10 91058 Erlangen Germany; ^2^ Institute of Micro‐ and Nanostructure Research (IMN) Center for Nanoanalysis and Electron Microscopy (CENEM) and Interdisciplinary Center for Nanostructured Films (IZNF) Friedrich‐Alexander Universität Erlangen‐Nürnberg Cauerstraße 3 91058 Erlangen Germany

**Keywords:** aggregation, amphiphiles, donor‐acceptor‐hybrids, perylenebisimide, porphyrinoids

## Abstract

We report the straightforward synthesis of a family of amphiphilic zinc‐porphyrin‐perylenebisimide (PBI) dyads containing sterically demanding substituents at the porphyrin *meso‐*position, as well as at the PBI bay‐position. The hydrophilic head group consists of oligo‐carboxylic acid G2‐Newkome dendrons attached to the porphyrin, whereas lipophilic pentyl‐hexyl‐swallowtails are connected to the PBI imide position. Using UV/Vis absorption and fluorescence emission spectroscopy, their tetrahydrofuran (THF) initiated disaggregation was studied in aqueous media. Without *meso*‐substituents, a stronger interaction between the porphyrins was observed in the aggregated state. Insights into the size and morphology of the aggregates were obtained by dynamic light scattering (DLS) and scanning transmission electron microscopy (STEM). Supported by density functional theory (DFT) calculations, these revealed micelles and liposomes as plausible architectures.

## Graphical abstract

We present the synthesis and characterization of water‐soluble porphyrin‐perylenebisimide dyads with varying sterically demanding side groups. The dyads strongly assemble in basic aqueous solutions, resulting in fully quenched fluorescence. Tracking the individualization through tetrahydrofuran (THF) by UV/Vis spectroscopy revealed stronger porphyrin‐porphyrin interactions for *meso*‐free dyads. STEM and DLS measurements supported by DFT models reveal micellular structures, as well as larger liposomes and agglomerates.

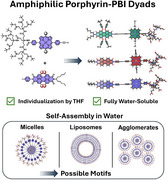

## 1 Introduction

Supramolecular self‐assembly lays at the foundation of many biological systems such as folded proteins, DNA, and membrane architectures.^[^ ^1^ ^]^ One particular high‐performance example is the light‐harvesting complex in photosynthesis.^[^ ^2^ ^]^ Generally, self‐assembly describes the formation of defined and stable aggregates in an equilibrium via noncovalent interactions.^[^ ^3^ ^]^ Taking inspiration from nature, applying the principles of self‐assembly to the design of new organic functional materials has led to the creation of a plethora of systems tailored to applications in, for example, organic solar cells^[^ ^4^ ^]^ or artificial photosynthesis.^[^ ^5^, ^6^ ^]^ Here, typical noncovalent interactions that guide self‐assembly include π–π stacking, hydrogen bonds, and hydrophobic forces. Consequently, gaining control over these interactions is highly important for the pursuit of stable architectures suitable for applications. This is especially essential for light harvesting, as the arrangement of the aggregates strongly influences the photophysical and electronic properties.^[^ ^6^, ^7^ ^]^

**Figure 1:**
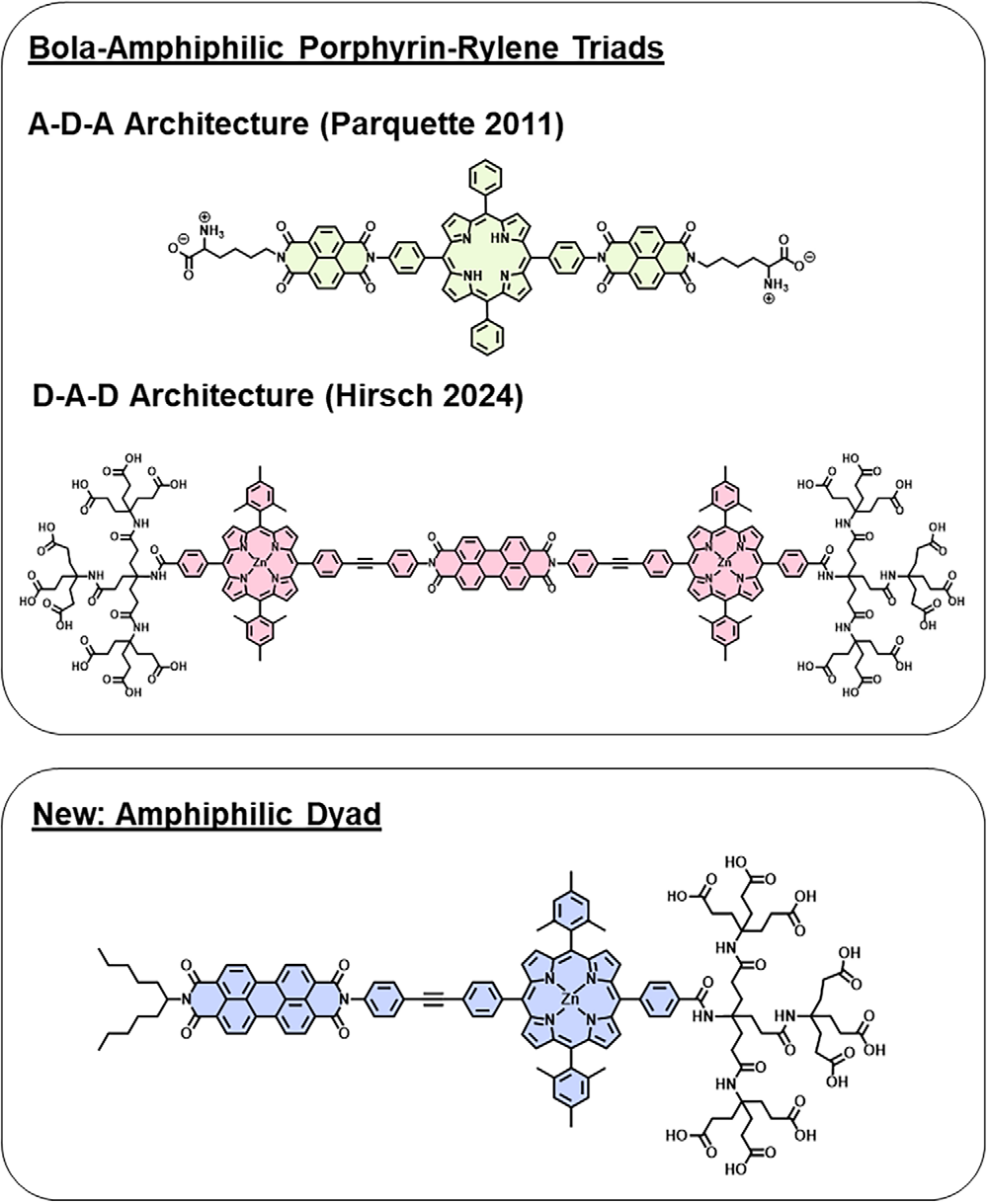
Schematic representation of top: bola‐amphiphilic porphyrin–rylene conjugates^[^ ^11^, ^30^ ^]^ bottom: a new amphiphilic porphyrin–PBI dyad.

In this context, the hierarchical organization of artificial electron donor‐acceptor (D‐A) systems, allowing for efficient photo induced charge separation, remains an attractive but challenging research field.^[^ ^8^ ^]^ Over the last years, impressive examples have been reported, combining a variety of donors and acceptors in amphiphilic motifs.^[^ ^9^, ^10^, ^11^, ^12^, ^13^, ^14^ ^]^ For example, the group of Parquette showed that bola‐amphiphilic porphyrin–naphthalenediimide conjugates assemble into nano‐tubular structures (Figure 1).^[^ ^11^, ^15^ ^]^ In the pursuit of new integrated D‐A amphiphiles and investigation of structure‐to‐function principles, the porphyrin–PBI couple is an excellent functional building block. It shows efficient charge transfer and separation, and both the individual chromophores and hybrid dyads have been extensively studied with respect to their photophysical and aggregation behavior.^[^ ^16^, ^17^, ^18^, ^19^, ^20^, ^21^, ^22^, ^23^, ^24^, ^25^, ^26^, ^27^, ^28^, ^29^ ^]^ Recently, we displayed the potential of oligo‐carboxylate Newkome dendrons in achieving water‐solubility of large bola‐amphiphilic porphyrin‐PBI triads (Figure 1).^[^ ^30^ ^]^ We demonstrated that the bola‐amphiphiles formed segregated stacks, rotated around an axis perpendicular to the PBI core. The steric hindrance induced by the mesityl‐groups at the *meso‐*position of the porphyrin suppressed a cofacial alignment of the porphyrins.^[^ ^30^ ^]^

However, the bola‐amphiphilic structure imposes restrictions, such as not allowing for stable micellular assemblies on the large scale and constricted interchromophore interactions on the small scale. To investigate the effects inflicted by these restrictions and targeting stable micelles, we aimed to expand the scope of amphiphilic porphyrin–PBI conjugates. Here, we report the synthesis of a family of fully water‐soluble porphyrin–PBI dyads based on asymmetrically functionalized PBIs and zinc‐porphyrins (Figure 1). These dyads were functionalized with different bulky substituents at the porphyrin and PBI to assess their impact on close‐range interactions and large‐scale aggregation behavior.

## 2 Results and Discussion

### 2.1 Synthesis

**Scheme 1:**
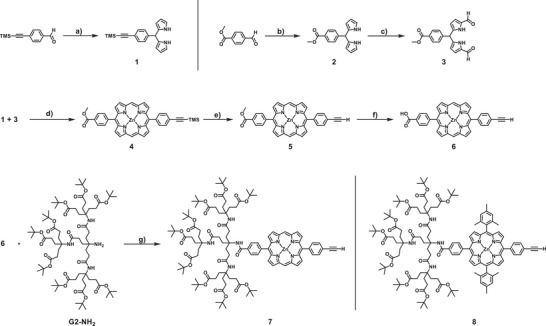
Synthesis of dendronized *meso*‐free AB_2_C porphyrin **7**; a) trifluoroacetic acid (TFA), pyrrole, rt, 10 minutes, 60%; b) TFA, pyrrole, rt, 10 minutes, 57%; c) DMF, POCl_3_, 0 °C, 1.5 hours, 21%; d) 1: 1‐propylamine, THF, rt, 1 hour; 2: Zn(OAc)_2_, toluene, air, reflux, 18 hours, 14%; e) TBAF, THF, rt, 3 hours, 95%; f) LiOH, THF/H_2_O, rt, 22 hours, 89%; g) HOBt•H_2_O, DCC, DMF, rt, 5 days, 45%; the synthetic procedures for **8** were reported previously.^[^ ^30^ ^]^

Our new architectures are based on bis‐phenylacetylene (tolane) as a linker between the building blocks for effective electronic ground‐state decoupling of the chromophores, and to maintain comparability with the bola‐amphiphilic system described previously (Figure 1).^[^ ^30^ ^]^ The synthesis of these dyads can be achieved either by an imidization reaction of a suitable amino‐tolane porphyrin or via Sonogashira cross‐coupling. Here, we opted for the cross‐coupling approach as it provides more flexibility and less harsh conditions. However, we could also show that the imidization approach works.^[^ ^31^ ^]^ To obtain the required porphyrin, a stepwise procedure via condensation of the respective dipyrromethane and the formylated dipyrromethane counterpart was chosen. This method was pursued in order to suppress the formation of side‐products and enabling an easier purification compared with statistical 2+2 condensation conditions.^[^ ^32^ ^]^ The synthesis of the required dendronized AB_2_C porphyrin **7** without substituents at the *meso*‐position (*meso*‐free) is displayed in Scheme 1. Dipyrromethanes **1** and **2** were obtained following literature procedures^[^ ^33^, ^34^ ^]^ in 60% and 57% yields, respectively. Formylation of **2** under Vilsmeier‐Haack conditions gave **3** in 21 % yield. Adapting a literature procedure by Lindsey,^[^ ^35^ ^]^ **3** was transformed into the respective propyl‐imine, and subsequent condensation with dipyrromethane **1** with zinc acetate under reflux gave **4** as the single porphyrin species in 14% yield. Here, toluene was chosen as the solvent. Regrettably, in ethanol, transesterification to ethyl benzoate was observed, despite the higher yields of the combined porphyrin species. The yield is not exceptionally good for this transformation, however, the ease of purification, as only one porphyrin species is formed, makes this route more attractive. With the porphyrin in hand, subsequent deprotection of trimethylsilyl‐acetylene with tetra‐*n*‐butylammonium fluoride (TBAF) in tetrahydrofuran (THF) at room temperature gave **5** in 95% yield. The methyl ester of **5** was saponified using lithium hydroxide in a water/THF mixture, affording benzoic acid porphyrin **6** in 89% yield. Finally, amide coupling of **6** with Newkome dendron **G2‐NH_2_**_,_ utilizing dicyclohexylcarbodiimide (DCC) and HOBt•H_2_O as coupling agents in dimethylformamide (DMF), afforded **7** in 45% yield. All *meso‐*free species are soluble in THF and dipolar aprotic solvents such as DMF and dimethylsulfoxide. The *meso*‐free porphyrins were fully characterized by ^1^H NMR and ^13^C NMR spectroscopy and by high‐resolution mass spectrometry (HRMS) (see Supporting Information). The ^1^H NMR spectrum (Figure S13) of **7** recorded in THF‐*d_8_* shows all the expected signals, with the *meso*‐protons being evident as a singlet at 10.29 ppm. The HRMS corroborates the successful synthesis with the molecular ion signal found at 2013.0305 *m/z* (calc.: 2013.0307 *m/z*).

To investigate the influence of the sterically demanding groups at the porphyrin and the PBI on the self‐assembly, dyads bearing mesityl groups at the porphyrin and *tert*‐butyl phenoxy groups at the bay‐position of the PBI were targeted. The synthetic details of the mesityl‐substituted porphyrin **8**, as well as the necessary iodo‐phenyl PBI precursors **9** and **10** were synthesized according to our recently published procedures.^[^ ^30^, ^36^ ^]^

**Scheme 2:**
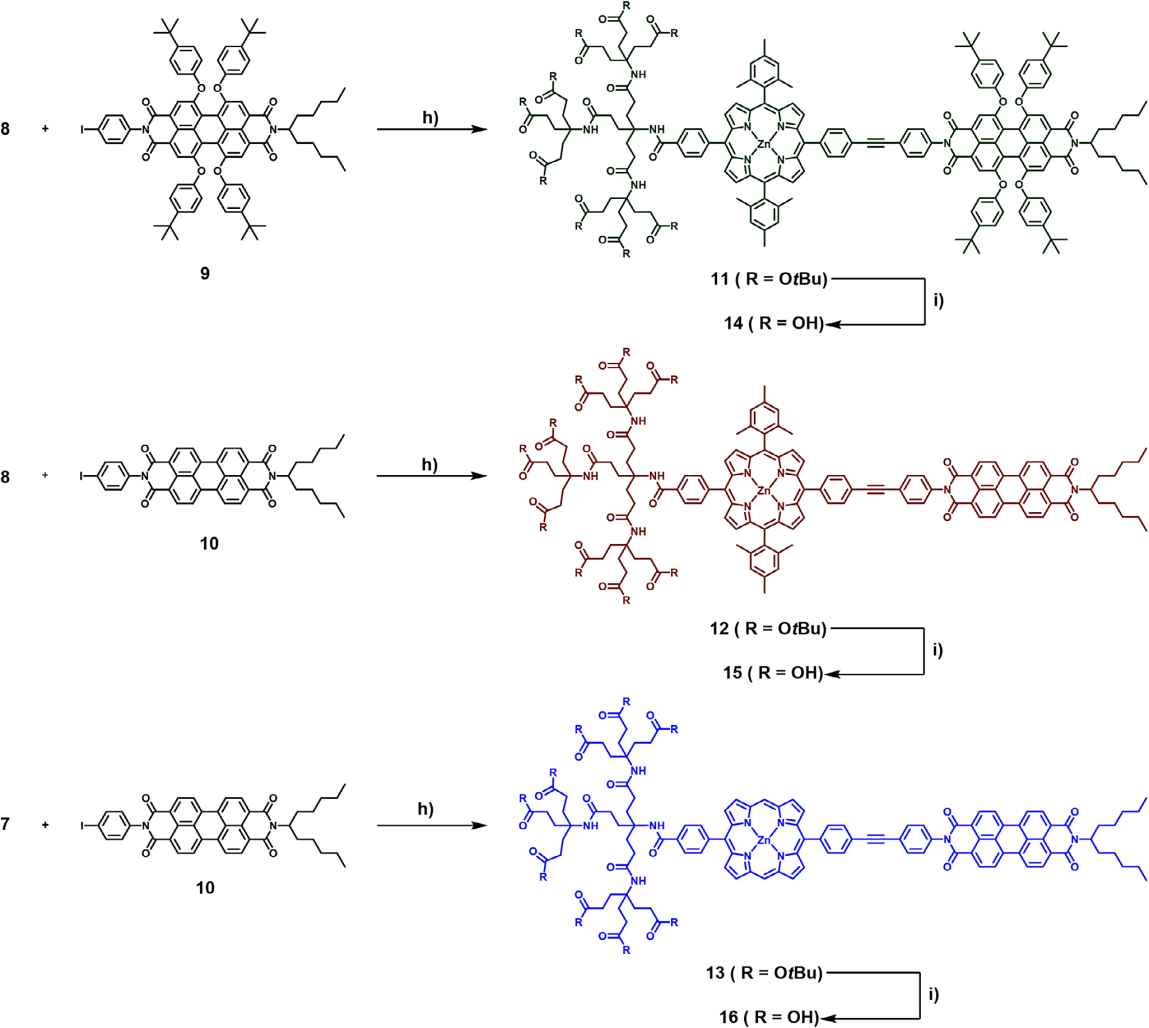
Synthesis of the amphiphiles **14**, **15**, and **16**; h) Pd(PPh_3_)_4_, CuI, PPh_3_, THF/NEt_3_, 85 °C, 20 hours, 84% (**11**), 59% (**12**), 52% (**13**); i) 1: HCOOH, rt, 3d, 2: Zn(OAc)_2_, THF, reflux, 12 hours, 99% (**14**), 93% (**15**), 70% (**16**); the synthetic procedures for **9** and **10** were reported by us previously.^[^ ^36^ ^]^

The synthesis of the final amphiphiles **14**, **15**, and **16** is displayed in Scheme 2. Subjecting dendronized acetylene porphyrins **7** and **8** and the respective iodo‐phenyl PBIs to Sonogashira conditions, using Pd(PPh_3_)_4_ and CuI as the catalytic system in a mixture of THF and NEt_3_ at 85 °C, gave the dyads **11**, **12**, and **13** in 84%, 59%, and 52% yield, respectively. Purification was achieved by gel permeation size‐exclusion chromatography using BioBeads SX1 in chloroform. All dyads display good solubility in common organic solvents and were fully characterized by NMR spectroscopy, as well as HRMS (see Supporting Information). The ^1^H NMR and ^13^C NMR of the dyads show all the expected signals, of both the parent porphyrin and perylene compounds without any significant changes. Matrix‐assisted laser desorption/ionization high resolution mass spectrometry (MALDI‐HRMS) for the dyads gives the molecular ion peaks for **11** at 3459.7947 *m/z* (calc. 3459.7943 *m/z*), **12** at 2867.4361 *m/z* (calc. 2867.4391 *m/z*), and **13** at 2631.2841 *m/z* (calc. 2631.2826).

**Figure 2:**
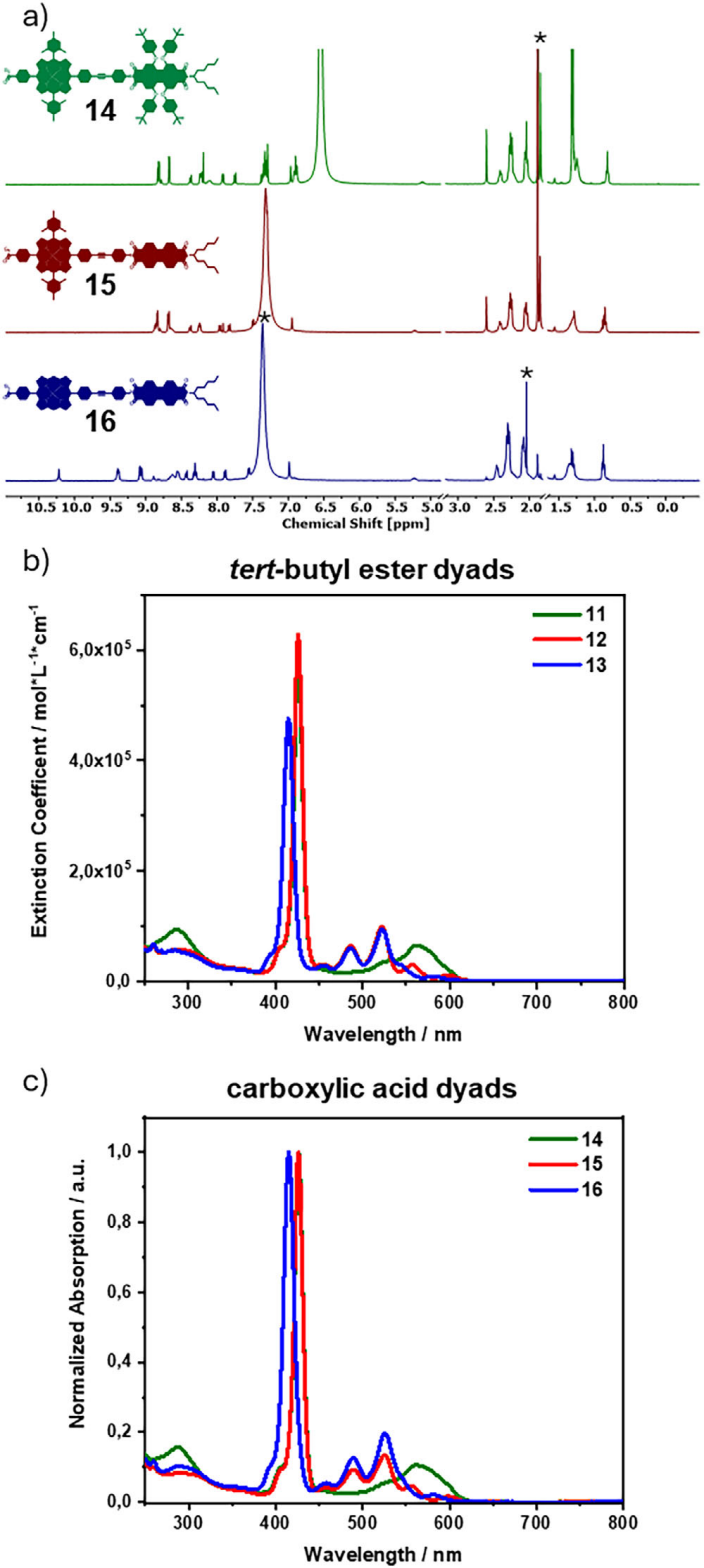
(a) NMR spectra (400 MHz, THF‐d_8_, 1 vol% TFA‐d, rt) of 14 (green), 15 (red), and 16 (blue), the asterisks mark residual signals of water and TFA; (c) Absorption spectra of 11, 12, and 13 in THF; (c) Normalized absorption spectra of 14, 15, and 16 in THF / 10 mM NaOH (aq) 1:1 v/v.

To obtain the oligo‐carboxylic acid amphiphiles **14**, **15**, and **16**, acidic cleavage of the *tert*‐butyl esters was carried out in neat formic acid at room temperature. Under these conditions, de‐metalation of the zinc‐porphyrin occurs, which requires subsequent re‐metalation. Therefore, upon azeotropic removal of the formic acid, the partially de‐metallated dyads were redissolved in THF and 4 eq. (for **14** and **16**) or 8 eq. (for **15**) of Zn(OAc)_2_ were added and heated to reflux for 12 hours. The progress of the of the re‐metalation was monitored by UV/Vis spectroscopy tracing the characteristic band of free‐base porphyrins at 650 nm. As the zinc ions can bind to up to two carboxylates, large networks of amphiphiles can be formed. Thus, workup was done by the addition of acetic acid to protonate the carboxylic acid groups, followed by precipitation after addition of heptane. Collection via filtration afforded the amphiphiles **14**, **15**, and **16**, in 93 %, 99%, and 70% yield, respectively. All amphiphiles were characterized by ^1^H NMR spectroscopy and HRMS. Unfortunately, we were unable to obtain ^13^C NMR spectra of the amphiphiles. Resolved spectra could only be obtained in THF‐*d_8_* / TFA‐*d* (1 vol%) mixtures in which the samples are not stable over time. The ^1^H NMR spectrum of the amphiphiles is shown in Figure 2a. Here, all the signals associated with the dyads can be observed, as well as the absence of the *tert*‐butyl ester peak (For **15**, the signals of the aromatic mesityl protons are overlayed by the TFA signal). Additionally, to assess the deprotection of the esters, attenuated total reflection infrared (ATR‐IR) spectra were recorded for all dyads (Figures S68, S69). Here, for the amphiphilic dyads, broad peaks at ṽ = 3306 cm^−1^ (**14**), 3290 cm^−1^ (**15**), and 3289 cm^−1^ (**16**) are observed, that are absent in the protected dyads. These correspond to O─H stretching signals of the carboxylic acids. Generally, all amphiphiles are soluble in basic aqueous media, as well as in acidic THF solutions, at the cost of being almost completely insoluble in other common organic solvents.

### 2.2 Steady‐State Absorption and Emission Spectroscopy

For all new dyads, UV/Vis absorption and fluorescence emission spectra were recorded. In the case of the dyads **11**, **12**, and **13**, absorption measurements were carried out in THF (Figure 2b). At first sight, the spectra of all three protected dyads resemble the sum of the individual chromophores, indicating weak ground‐state interactions. For **11**, the Soret‐band (S_0_→S_2_ transition) is evident at 426 nm, while the absorption of the porphyrin Q‐bands (S_0_→S_1_ transitions) and PBI are overlapping, resulting in only two maxima at 530 nm and 562 nm. In contrast, **12**, bearing the more electron deficient unsubstituted PBI core, does exhibit distinct porphyrin and PBI absorptions. Here, the Soret‐band is evident at 426 nm and the corresponding Q‐bands at 557 nm and 597 nm. The features stemming from the PBI can be found at 455 nm, 486 nm, and 522 nm. Finally, the *meso‐*free dyad **13** also shows distinctive features, with the Soret‐ and Q‐bands slightly blue‐shifted to 415 nm, 544 nm (shoulder), and 582 nm. The corresponding PBI signals can be observed at the same wavelength as for **12** (455 nm, 486 nm, and 522 nm). Additionally, when looking at the features stemming from the individual chromophore units (mesityl‐porphyrin for **11** and **12**, and the unsubstituted PBI for **12** and **13**), matching extinction coefficients are observed, corroborating the close to zero ground‐state interactions.

The corresponding fluorescence emission spectra for the *tert*‐butyl ester dyads **11**, **12**, and **13**, recorded in THF (Figures S49–S51) show the typical porphyrin and PBI emissions.

Upon excitation at the respective Soret‐band, emission bands at 604 nm and 655 nm (for **11** and **12**) and 590 nm and 638 nm (for **13**) can be observed. These agree with emission wavelengths observed for the zinc‐porphyrin precursors. Additionally, signals resembling PBI emission can be observed for **12** and **13** at 531 nm and 570 nm (partly overlapped by the porphyrin emission in **13**).

Upon excitation of **12** and **13** at the PBI (485 nm), four bands are observed, with significant emission of the porphyrin. Presumably, this originates from an energy transfer from the PBI (Por‐PBI*) to the porphyrin (Por*PBI), followed by fluorescence emission, as evident in comparable systems in the literature.^[^ ^20^ ^]^ For **11**, excitation at 525 nm leads to similar emission spectra than when exciting at the porphyrin, with slightly different shape. We ascribe this on the one hand to the overlap of the absorption of both chromophores at this wavelength and on the other hand to a possible energy transfer from the excited‐state Por–PBI* to Por*‐PBI, as with the other dyads. The emission spectra could not be deconvoluted, and therefore no further statement can be made about the relative intensities.

For the amphiphilic dyads **14**, **15**, and **16**, UV/Vis absorption spectroscopy was carried out in THF / 10 mM aqueous NaOH (1:1 v/v) mixtures (Figure 2c). This solvent was chosen, as under these conditions, the amphiphiles are expected to be mostly individualized and all carboxylic acids mostly deprotonated at pH ≈ 10, establishing uniformity of the system. Absorption spectra in pure phosphate buffered solutions at pH 7.2 and pH 12 led only to marginally different spectra (Figure S60) compared to pure NaOH solution.

In the THF/aqueous NaOH 1:1 mixture, the recorded spectra almost completely resemble the spectra of the *tert*‐butyl ester dyads in THF, with only marginal changes, which can be ascribed to the change in overall solvent polarity. However, the obtained extinction coefficients are significantly lower than of the non‐amphiphilic precursors (Figure S52). This can be caused by the change in solvent environment and the still remaining presence of loosely aggregated species. Similarly, fluorescence emission spectra recorded in the same solvent mixture (THF/10 mM aqueous NaOH (1:1 v/v) of **14**, **15**, and **16** show the same emission spectra as their *tert*‐butyl ester precursors regarding the porphyrin emission. However, small changes in relative emission intensity of PBI to porphyrin emission, as well as shifts in the PBI emission itself can be observed when the PBI is excited (see Figure S59). We attribute this to the change in solvent environment, which also affects the energy transfer rates. This corroborates that the amphiphiles are mostly individualized in this solvent and at this concentration range.

### 2.3 Aggregation–Disaggregation Studies

To gain insight into the aggregation behavior and the influence of the sterically demanding substituents, UV/Vis absorption and fluorescence emission spectra were recorded in varying mixtures of THF and 10 mM aqueous NaOH, while keeping the concentration of the respective amphiphile constant. Consequently, shifts in the absorption maxima and changes in fluorescence emission were used as monitoring parameters. All samples were freshly prepared and measured 1 minute after preparation. Measurements over the course of an hour only marginally changed the absorption and emission spectra. However, longer aging over the course of two days leads to significant, concentration dependent changes of the absorption spectra (**Figure** S61). This indicates the formation of thermodynamically more stable conformations and a complex assembly energy landscape. For the scope of this work, only the initially formed aggregates shall be discussed in the following in detail.

**Figure 3:**
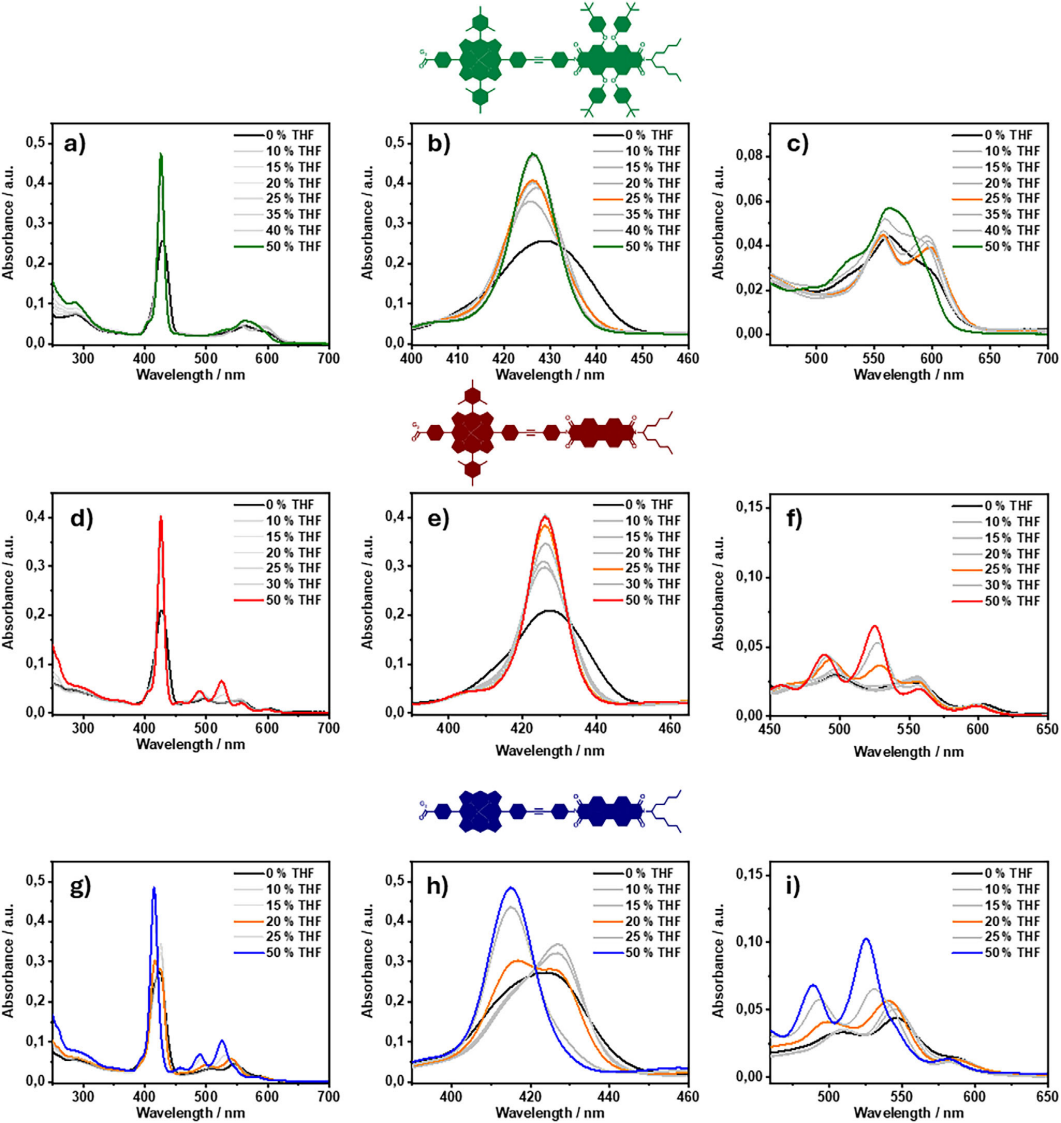
(a–c) UV/Vis absorption spectra of **14** (c = 1.42 × 10^−6^ M) in 10 mM aqueous NaOH with different parts of THF as indicated, (b,c) are partial spectra of (a); (d–f) UV/Vis absorption spectra of **15** (c) = 1.39 × 10^−6^ M) in 10 mM aqueous NaOH with different parts of THF as indicated, (e,f) are partial spectra of (d); (g–i) UV/Vis absorption spectra of **16** (c) = 1.38 × 10^−6^ M) in 10 mM aqueous NaOH with different parts of THF as indicated, (h) and (i) are partial spectra of (g); the complete spectra with all ratios of THF color coded can be found in the supporting information.

Figure 3 shows the absorption spectra of the amphiphilic dyads in 10 mM aqueous NaOH solutions with different portions of THF. For clarity, only three THF ratios are depicted in color. An assignment of all ratios can be found in the Supporting Information. At first sight, in pure basic aqueous solution (black lines), a broadening of the Soret‐band absorption, a loss of distinctive vibronic features of the PBI and a decrease in absorbance takes place for all amphiphiles.

Looking at the absorption of amphiphile **14** in a pure aqueous solution (Figure 3b, black), the Soret‐band maximum is slightly red‐shifted to 429 nm. The overlapping Q‐bands and PBI features show a maximum at 561 nm with a shoulder at approximately 597 nm (Figure 3c, black). When the THF concentration is increased to 25 vol% THF (Figure 3a–c, orange), the Soret‐band gains in intensity and shows a small blue‐shift. In addition, two distinctive bands appear at 557 nm and 598 nm. When reaching 50 vol% (Figure 3, green), the features of the dissolved dyad reemerge, with the absorption bands described above. The features at 25 vol% THF are distinct from the fully aggregated and monomeric species. This points to a non‐linear transition from the aggregated to monomeric state, with reorganization to different assemblies taking place over the course of THF addition.

The absorption spectra of amphiphile **15**, bearing the unsubstituted PBI and the mesityl porphyrin, are displayed in Figure 3d–f. In a pure aqueous medium, the Soret‐band also undergoes a small red‐shift to 427 nm compared to 50 vol% THF (Figure 3e, black). The PBI bands at 471 nm, 498 nm, and 542 nm are also red‐shifted. The Q‐bands appear at 556 nm and 603 nm, showing only a marginal red‐shift compared to the 50 vol% THF solution (Figure 3f, black). Upon gradual increase of the THF concentration up to 50 vol%, a continuous trend toward well‐resolved features can be observed. The Soret‐band (Figure 3e, red) gains in intensity and sharpness, while shifting slightly to 426 nm. The PBI‐bands also gain absorbance and significantly shift toward 458 nm, 489 nm, and 525 nm. Interestingly, the Q‐bands stay almost unchanged and experience only a slight shift which is attributed to the change in solvent polarity (Figure 3f, red).

Finally, the absorption spectra of **16** in different solvent mixtures of THF and aqueous NaOH are displayed in Figure 3g–i. In pure aqueous NaOH, the Soret‐band can be observed as a broad signal at $\lambda_{\max}=\;$424 nm (Figure 3h, black). The PBI‐bands are broadened and red‐shifted, and partially overlapped with the Q (1,0) band, resulting in two maxima at 509 nm and 546 nm, as well as a shoulder at 585 nm (Figure 3i, black). Furthermore, the PBI (2,0) band can no longer be observed. With increasing amounts of THF, the Soret‐band first shows a red‐shift toward 427 nm, while gaining in intensity and sharpness. Upon 20 vol% of THF (Figure 3h, orange) a split Soret‐band can be observed, with two maxima at 425 nm and 417 nm, where the first band decreases again in intensity. This trend continues over the addition of more THF. At 50 vol% (Figure 3h, blue), the original absorption at 415 nm is observed. The absorption features of the PBI show a continuous blue‐shift and an increase in absorption and sharpness when adding up to 50 vol% of THF (Figure 3i, orange / blue). Additionally, the PBI (2,0) band is reinstated between 20–25 vol%. The Q (0,0) band shows a small continuous blue‐shift, while the Q (1,0) band can be observed as a shoulder at around 542 nm, as described above. Similar to **14**, the spectra at 15 vol% THF do not resemble an intermediate state between fully aggregated and individualized, indicating a non‐linear transition between the species.

Generally, the addition of larger THF amounts than 50 vol% lead to reaggregation and finally to precipitation (**Figures** S53–S55).

**Figure 4:**
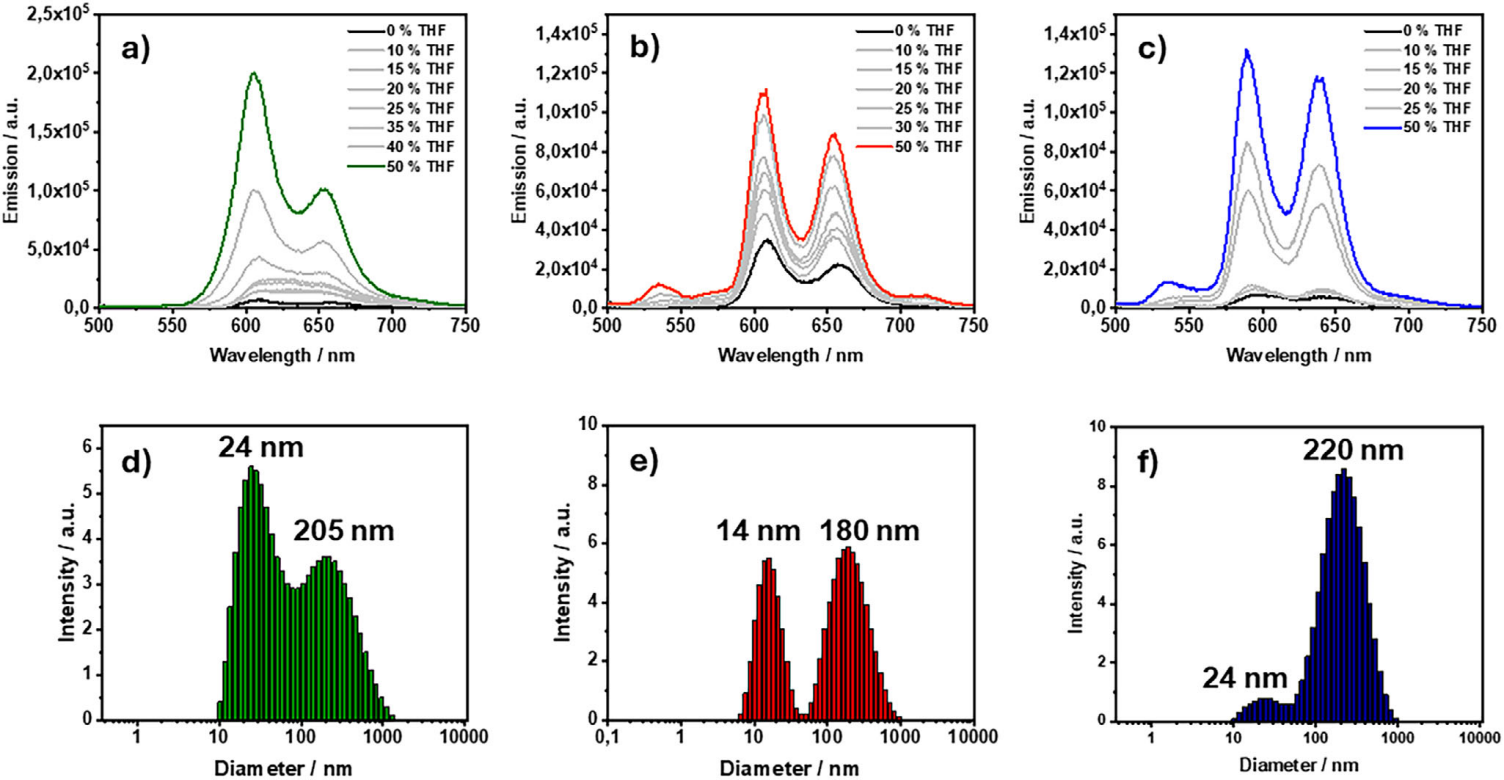
(a–c): Fluorescence emission spectra of (a) **14** (c) = 1.42 × 10^−6^ M) (b) **15** (c = 1.39 × 10^−6^ M) (c) **16** (c) = 1.38 × 10^−6^ M) in in 10 mM aqueous NaOH with different parts of THF as indicated, the complete spectra with all ratios of THF color coded can be found in the supporting information; (d–f): DLS intensity size distributions of **14** (c) = 1.3 × 10^−6^ M) (b) **15** (c) = 2.1 × 10^−6^ M) (c) **16** (c) = 9.4 × 10^−7^ M) in phosphate buffered solution (pH 12), average of three measurements; the given values are the peaks of their underlying distributions.

To further verify the individualization process of the aggregates, fluorescence emission spectra were recorded. Upon excitation at the Soret‐band (Figure 4a–c), a strong quenching of the fluorescence can be observed in pure aqueous solution (black). Upon addition of THF, a continuous recovery of the initial fluorescence properties can be observed. Excitation at the PBI (Figure S56–S58) displays the same trend. It must be noted that the change in absorbance at the excitation wavelengths during the experiment also influences the emission intensity. This suggests that the amphiphiles are strongly aggregated in pure aqueous solutions, followed by individualization upon THF addition.

When comparing the results of the amphiphiles, a clear change in behavior of the *meso*‐free dyad **16** can be observed. Here, a close interaction between the porphyrin and their neighboring chromophore is evident. This manifests in a large red‐shift upon aggregation, as well as the presence of two distinct Soret‐bands as an intermediary state. Pronounced shifts of the PBI‐bands can be observed in all dyads, indicating strong interactions of the PBIs moieties. When taking this into consideration, an aggregation motif of the dyads being offset by an angle with overlapping PBIs can be imagined. In the *meso*‐free dyad, the porphyrins come in closer contact with each other due to the absence of the bulky mesityl groups. However, the distance between the *meso*‐free porphyrins is limited by the columbic and steric repulsion of the dendrons. Furthermore, as mentioned above, for **14** and **16**, the transition toward the individualized species does not appear to progress linearly, indicating complex individualization behavior. This may include possible reorganization into different aggregates. Consequently, further quantitative analysis by fitting isodesmic or (anti‐) cooperative models is not implemented at this point. The THF induced individualization of the aged solutions displayed trends comparable to the fresh solutions (Figure S62).

### 2.4 Theoretical and Morphological Investigations

To be able to assess the morphology and structure of the large‐scale aggregates, the electronic and geometric structures of the singular dyads were modelled at the DFT level of theory (Figures S63–S66; for details we refer to the Supporting Information). Geometry optimization in the gas phase reveals that the porphyrin and the PBI are co‐planar in respect to each other, with the whole dyads having an approximate length of 5.5 nm. For all amphiphiles, the HOMO is calculated to be exclusively located at the porphyrin, while the LUMO is found at the PBI. The resulting HOMO‐LUMO gaps are calculated to be Δ_HOMO‐LUMO _= 2.06 eV (**14**), 1.66 eV (**15**), and 1.77 eV (**16**).

**Figure 5:**
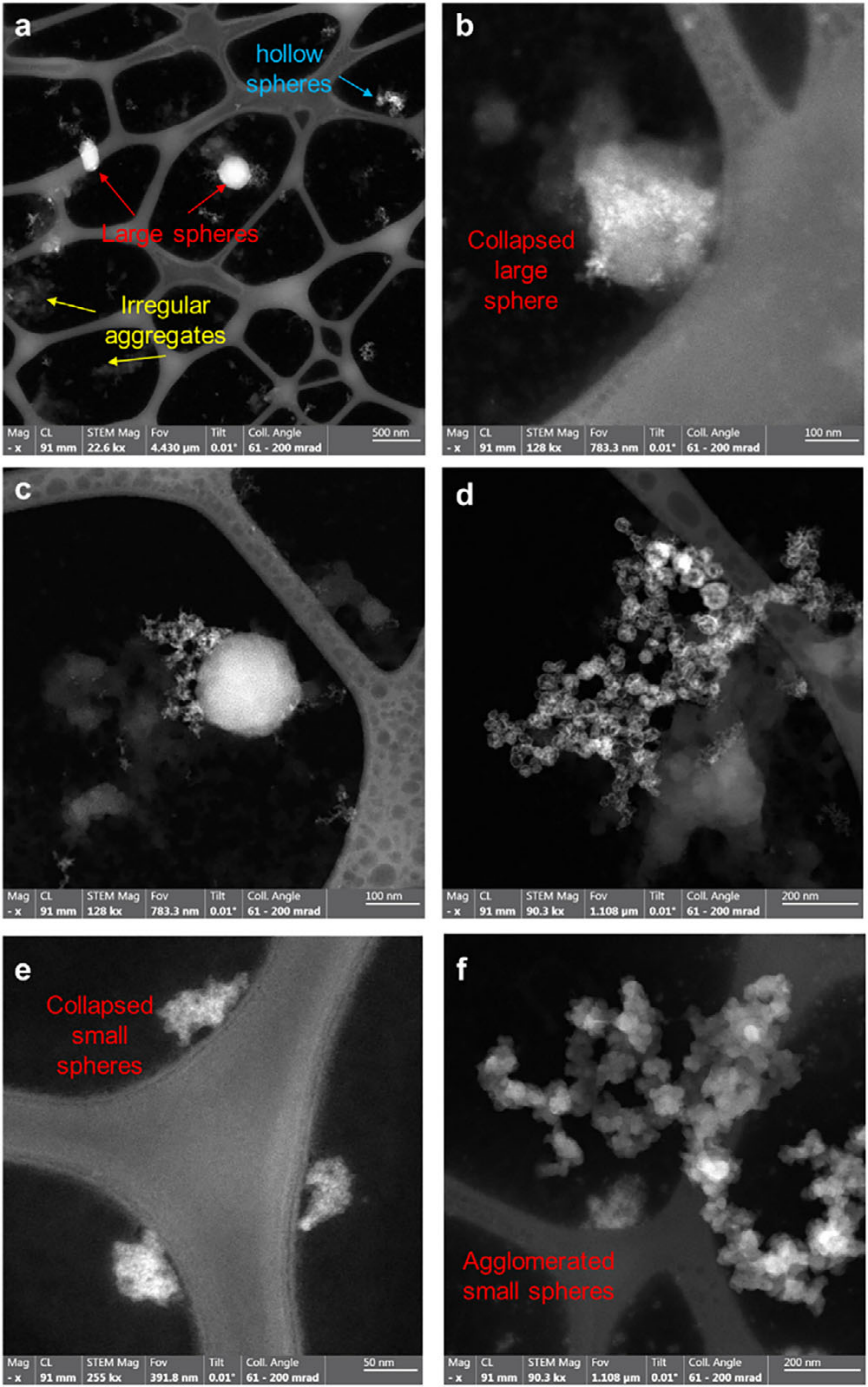
STEM‐HAADF image of 14 (after ∼18 hours (a,b) and ∼1 hour (c,d) of drying, respectively), 15 (e), and 16 (f). Various types of assemblies and aggregates are observed.

To reveal the morphology of the assembled amphiphiles **14**, **15**, and **16**, scanning transmission electron microscopy (STEM) imaging with high‐angle annual dark‐field contrast (HAADF, also known as Z‐contrast) was carried out (Figure 5). Samples were drop‐casted on a TEM grid and dried between 1 hour and 15 hours (for details see Methods). Generally, we observe the dried states of the samples in the vacuum of TEM, and therefore, influences due to the drying process as well as the surface on the 3D assemblies have to be considered. Nevertheless, the observed morphology of the samples still offers valuable hints and insights into their assembled states. For **14** (Figure 5a–d) large spheres were observed, in the size range of 100 nm – 200 nm. Further, smaller spheres of approximately 20 nm – 50 nm are evident. From the enhanced image intensity at the rim of these spheres (of ∼3 nm) in the Z‐contrast image and 2D projection of 3D objects, it is reasonable to interpret these spheres are of hollow nature. Additionally, smaller irregular agglomerates and large undissolved particles can be observed. Zn signals, as fingerprint of **14**, were detected with EDXS (Figure S70) from all these agglomerates. We further studied this sample dried in air for ∼18 hours and ∼1 hour, respectively. Interestingly, a lot more, hollow spheres are revealed in the sample after ∼1 hour drying compared to the ∼18 hours drying sample. This suggests that the small, hollow spheres collapse into irregular agglomerates after prolonged drying.

When examining images obtained from **15** (Figures 5e, S71), small regular particles in the size range of 20 nm – 50 nm become evident. Furthermore, agglomerates of several such small aggregates are observed. Similar to **14**, irregularly agglomerated particles are observed as well. Lastly, the images obtained from amphiphile **16** (Figures 5f, S72) reveal comparable results to **15**. Here, small regular spherical particles in the size range of 20 nm –40 nm and the respective agglomerates are observed as well. However, these are structurally more defined and not as diffuse as those of **15**. These are also accompanied by irregular, loosely aggregated particles.

**Figure 6:**
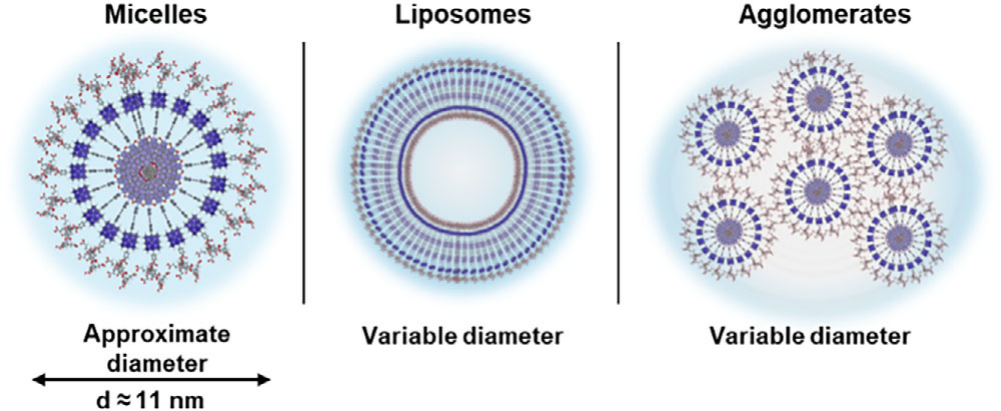
Possible aggregation motifs of the amphiphilic dyads under the assumption of spherical aggregates; the diameter of the micelle is approximated as two times the length of a single amphiphile.

We ascribe the small regular structures observed for **15** and **16** to micellular aggregates which tend to agglomerate (Compare Figure 6). However, it remains elusive to what extent agglomeration occurs in solution or how drying affects agglomeration. The larger structures of **14** are thought to be spherical liposome structures. The observed loose particles can be reasonably attributed to collapsed and dried assemblies, as exemplarily shown through variation of drying times of amphiphile **14**.

To gain more information about the assemblies in solution, dynamic light scattering (DLS) measurements (Figure 4d–f) were recorded in phosphate buffered solutions (pH 12, **14** (c = 1.3 × 10^−6^ M) **15** (c = 2.1 × 10^−6^ M) **16** (c = 9.4 × 10^−7^ M)) as well as 10 mM NaOH solutions (Figure S67). As the diffractive index of the sample is not known to us, further evaluation of the number and weight distribution was not done. However, when looking at the size distribution intensity, for all amphiphiles two main size regimes can be observed, from 10 nm to 100 nm, and a broader signal from 100 nm to 1 µm. We ascribe the smallest observed signals to micellular organized amphiphiles, as the diameter is in the range of two times the length of the amphiphiles. This is in good agreement with the small particles observed in the STEM images. The larger sizes stem from either liposomes or counter‐ion mediated agglomerates of the micelles. Interestingly, at lower concentrations (1/5^th^ of the respective solutions), the relative intensity of the smaller sizes decreased in respect to the 100 nm – 1 µm arrangements (Figure S67).

## 3 Conclusion

In summary, we accomplished the synthesis and full characterization of a family of three amphiphilic porphyrin–PBI dyads through Sonogashira cross‐coupling methodology of the respective asymmetric chromophores. Water solubility was realized by attaching large oligo‐carboxylic acid Newkome dendrons to the porphyrin. The dyads vary in sterically demanding side groups, namely, mesityl at the *meso*‐position of the porphyrins and *tert*‐butyl phenoxy at the bay‐position of the PBIs. UV/Vis absorption and fluorescence emission spectroscopy in THF/aqueous NaOH mixtures revealed the aggregation–disaggregation process of the initially formed supramolecular architectures. Preliminary time‐dependent measurements suggest the presence of other thermodynamically more stable aggregates and complex assembly behavior. The quenching of the fluorescence in the aggregates provides evidence of efficient charge separation. First insights into the effect of the side groups on close‐range aggregation motifs were gained. In detail, the *meso‐*free amphiphile showed a significant change in porphyrin absorption, indicating stronger intermolecular interactions of the porphyrins. Assessment of the morphology via STEM imaging accompanied by DLS measurements reveal the presence of micelles and their agglomerates, as well as liposome structures.

This set of donor‐acceptor amphiphiles rationally expands the family of porphyrin–PBI amphiphiles. Moreover, it provides an excellent starting point for investigating the influence of stronger porphyrin–porphyrin interactions on the photoinduced charge‐transfer properties in the aggregated state. Ultimately, it creates a stable confined environment by forming micellular structures in water. However, in order to definitely control the assembled structures, the complex assembly landscape needs to be further understood in more detail, which is currently being investigated in our laboratory. In the future, we envisage the possibility of using this confined environment as a reaction space to facilitate the conversion of solar energy into chemical energy.

## 4 Experimental Section

The detailed synthetic procedures and data used for characterization are provided in the Supporting Information.

### General Sonogashira cross‐coupling procedure

A oven dried microwave vial was charged with the respective dendronized acetylene porphyrin (**7**/**8**, 1.1 eq.), the asymmetric 4‐iodo‐phenyl‐PBI (**9**/**10,** 1.0 eq.), Pd(PPh_3_)_4_ (10–15 mol%), CuI (20–30 mol%) and PPh_3_ (20–30 mol%)). The vial was sealed and then evacuated and backfilled with nitrogen three times. To this, a prior degassed mixture of THF and NEt_3_ (2:1 v/v, c_PBI _= 2.5 − 3.3 mM) was added and the mixture was heated to 85 °C for 20 h. Then, the mixture was allowed to cool to room temperature and filtered through a plug of silica gel (eluent: Tol / THF 5:1 v/v). The resulting crude product was further purified by gel‐permeation size exclusion chromatography (CHCl_3_ Ø 2.5 cm, ↑ 60 cm) yielding the desired dyads **11 **(84 %), **12** (59%), and **13** (52%).

### General dendron deprotection procedure

In a 10 mL microwave vial, the respective *tert‐*butyl ester dyad (**11**–**13**) was dissolved in formic acid (c_dyad _≈ 1.1 mM) under a nitrogen atmosphere. The reaction mixture was stirred under the exclusion of light at room temperature for 3 days. Then, the solvent was removed azeotropically with toluene (three times) under reduced pressure. The remaining solid was redissolved in THF (5 mL) and Zn(OAc)_2_ (4 eq.) was added and the mixture was stirred at 55 °C for 12 hours to re‐metalate the dyad. When the dyad was fully metalated as judged by UV/Vis spectroscopy, the mixture was slightly acidified with a few drops of acetic acid and heptane was added to precipitate the dyad. The precipitate was collected by filtration and washed with H_2_O and acetone, yielding the desired amphiphiles **14 **(99%), **15** (93%), **16** (70%).

### UV/Vis / Fluorescence aggregation studies

Amphiphiles **14**, **15**, and **16** were dissolved in a freshly prepared solution of 10 mM NaOH in water (Milli‐Q). In separate screw‐cap vials, each stock‐solution was diluted with the corresponding amounts of 10 mM NaOH(aq) and THF (total volume = 3 mL) to give the respective mixtures (c(**14**) = 1.42 × 10^−6^ M; c(**15**) = 1.39 × 10^−6^ M; c(**16**) = 1.38 × 10^−6^ M). Each solution was then measured separately after a curing time of 1 minute during which the vials were kept tightly sealed to minimize evaporation of the solvent. UV/Vis and fluorescence measurements were carried out in direct succession in the same cuvette.

### STEM

The samples for STEM studies were prepared via a simple drop‐casting method on lacey‐ultrathin 400 mesh Cu grid (#0 1824, Plano). The concentrations of the aqueous solutions were 1 mM (for **14**) and 2 mM (for **15**, **16**). Sample **14** was to dried on air for 1 hours and 15 hours, **15** for 1 hour, and **16** for 15 hours. STEM experiments were performed on a double Cs‐corrected Thermo Fisher Scientific Titan Themis microscope operated at 300 kV. The microscope is equipped with a Super‐X EDXS (energy dispersive X‐ray spectroscopy) system. The HAADF images were recorded with an aberration corrected probe with 22 mrad and collection angular range of 60–300 mrad, typical to deliver Z‐contrast images.

## Supporting Information

The authors have cited additional references within the Supporting Information.^[^ ^33^, ^34^, ^37^ ^]^

## Conflict of Interests

The authors declare no conflict of interest.

## Supporting information



Supporting Information

## Data Availability

The data that support the findings of this study are openly available in Zenodo at http://doi.org/10.5281/zenodo.13912541.
