# Supplementary material for: Synthesis and Aggregation of Amphiphilic Porphyrin‐Perylenebisimide Dyads
Source: Chemistry. 2025 May 2;31(31):e202500279. doi: 10.1002/chem.202500279 (PMC12133624; doi:10.1002/chem.202500279)
Supplement: Supplementary file 1 — Supporting Information [file CHEM-31-e202500279-s001.pdf]

Table of content

1. **Materials and Methods** 2
2. **Synthesis** 3
3. **NMR Spectra** 16
4. **Mass Spectra** 32
5. **UV/Vis and Fluorescence Spectra** 39
6. **Theoretical Calculations** 51
7. **Dynamic Light Scattering (DLS)** 54
8. **IR-Spectra** 55
9. **STEM Imaging** 57
10. **References** 61

1. Materials and Methods

Chemicals were purchased from Sigma-Aldrich and TCI and used without any further purification. Solvents were distilled prior to usage. Solvents were degassed by three cycles of ultrasonication under vacuum followed by subsequent refilling with a protective gas (Ar or N_2). Thin layer chromatography (TLC) was performed on Merck silica gel 60 F524, detected by UV-light (254 nm, 366 nm). Silica gel plug filtration and column chromatography were performed on Macherey-Nagel silica gel 60 M (deactivated, 230−400 mesh, 0.04−0.063 mm). Gel permeation size exclusion chromatography was performed on BioBeads SX1 from BioRad in $CHCl_3$.

NMR spectra were recorded on a Bruker Avance 400 (^{1}H: 400 MHz, ^{13}C: 101 MHz), a Bruker Avance 500 (^{1}H: 500 MHz, ^{13}C: 126 MHz), or a Bruker Avance Neo Cryo-Probe DCH (^{1}H: 600 MHz, ^{13}C: 150 MHz). Deuterated solvents were purchased from Sigma-Aldrich, ARMAR Isotopes and Deutero and used as received. Chemical shifts are given in ppm at room temperature and are referenced to residual protic impurities in the solvents (^{1}H: $CDCl_3$: 7.24 ppm, CD_2Cl_2: 5.34 ppm, THF-d_8: 3.58 ppm, D_2O: 4.79 ppm) or the deuterated solvent itself (^{13}C{^{1}H}: $CDCl_3$: 77.16 ppm, CD_2Cl_2: 53.4 ppm, THF-d_8: 66.57 ppm). The resonance multiplicities are indicated as “s” (singlet), “brs” (broad singlet), “d” (doublet), “t” (triplet), “q” (quartet) and “m” (multiplet). Mass spectrometry was carried out with a Shimadzu AXIMA Confidence (MALDI-TOF, matrix: 2,5-dihydroxybenzoic acid DHB, trans-2-[3-(4-tert-butylphenyl)-2-methyl-2-propenyliden]-malononitrile, (DCTB) or without matrix (OM). High resolution mass spectrometry (HRMS) was recorded on a LDI/MALDI-ToF Bruker Ultraflex Extreme machine or on a micrOTOF II (ESI- / APPI–TOF) focus mass spectrometer (Bruker).

UV/vis spectroscopy was carried out on a Varian Cary 5000 UV−vis−NIR spectrometer. The spectra were recorded at room temperature in deoxygenated solutions in quartz cuvettes (edge length = 1 cm) under ambient conditions. Fluorescence spectra were obtained from a Shimadzu RF-5301 PC and a NanoLog spectrofluorometer. Dynamic Light Scattering (DLS) was performed on a Zetasizer Nano Series ZEN3600 (Malvern Instruments) with a 633 nm He-Ne laser. IR was measured on an PerkinElmer Frontier FT-IR spectrometer equipped with a PerkinElmer “Universal ATR Sampling Accessory” unit.

2. Synthesis

The synthetic procedures for **8**,[1] **9** and **10**[2] were published by us earlier.

5-(4-Methoxycarbonylphenyl)dipyrromethane (1)[3]

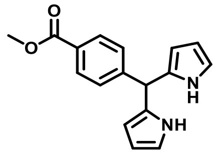

Methyl-4-formylbenzoate (3.50 g, 21.3 mmol) was dissolved in pyrrole (80.0 mL, 77.6 g, 1.16 mol, 55 eq). The solution was degassed with N_2 for 15 min under the exclusion of light. $BF_3 \cdot Et_2O$ (0.50 mL, 0.56 g, 4.00 mmol, 0.2 eq) was added and the reaction mixture was stirred for 10 min under light exclusion. After addition of an aqueous solution of NaOH (0.1 M, 50 mL) and DCM (50 mL), the phases were separated, the aqueous phase was extracted with DCM (20 mL) and the organic phase was washed with water (50 mL). The combined organic phases were dried over Na_2SO_4 and the solvent was removed under reduced pressure. After recrystallization from DCM/Pentane, compound **1** (3.58g, 12.8 mmol, 60%) was obtained as a white powder.

^{1}H NMR (400 MHz, THF-d_8): δ = 9.78 (s, 2H), 7.90 (d, J = 8.4 Hz, 2H), 7.27 (d, J = 8.1 Hz, 2H), 6.65 – 6.53 (m, 2H), 5.98 – 5.88 (m, 2H), 5.74 – 5.59 (m, 2H), 5.45 (s, 1H), 3.82 (s, 3H) ppm.

^{13}C NMR (101 MHz, THF-d_8): δ = 167.11, 150.15, 133.32, 130.18, 129.57, 129.51, 118.09, 108.20, 108.01, 52.11, 45.39 ppm.

MS (MALDI-TOF, dctb) m/z for $C_{17}H_{16}N_2O_2$: $[M]^+$ calculated: 280.12, found: 280.1175

5-[4-(2-(Trimethylsilyl)ethynyl)phenyl]dipyrromethane (2)[4]

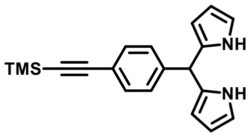

4-[(Trimethylsilyl)ethynyl]benzaldehyde (2.16 g, 10.7 mmol) was dissolved in pyrrole (40.0 mL, 38.8 g, 0.58 mol, 55 eq). The solution was degassed with N_2 for 10 min under the exclusion of light. $BF_3 \cdot Et_2O$ (0.25 mL, 0.28 g, 2.00 mmol, 0.2 eq) was added and the reaction mixture was stirred for 10 min under light exclusion. After addition of an aqueous solution of NaOH (0.1 M,

25 mL), the phases were separated, and the aqueous phase was extracted with DCM (20 mL). The combined organic phases were dried over Na_2SO_4 and the solvent was removed under reduced pressure, resulting in a viscous oil. The oil was dissolved in DCM and layered with *i*-hexane, yielding a solid which was subsequently recrystallized from ethanol and water. The precipitate was filtered and washed with methanol, yielding compound **2** (1.93 g, 6.05 mmol, 57%) as a beige solid.

^{1}H NMR (400 MHz, THF-d_8): δ = 9.73 (s, 2H), 7.35 – 7.25 (m, 2H), 7.18 – 7.09 (m, 2H), 6.64 – 6.53 (m, 2H), 5.96 – 5.86 (m, 2H), 5.69 – 5.58 (m, 2H), 5.37 (s, 1H), 0.21 (s, 9H) ppm.

^{13}C NMR (101 MHz, THF-d_8): δ = 145.67, 133.56, 132.46, 129.51, 122.20, 117.97, 108.14, 107.92, 106.51, 93.67, 45.25, 0.23 ppm.

MS (MALDI-TOF, dctb) m/z for $C_{20}H_{21}N_2Si$: $[M-H]^+$ calculated: 317.1474, found: 317.1445

1,9-Diformyl-5-(4-Methoxycarbonylphenyl)dipyrromethane (3)[3]

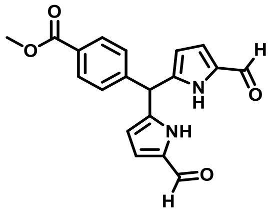

Under a N_2-atmosphere, $POCl_3$ (1.50 mL, 16.4 mmol) was slowly added to degassed DMF (10 mL), and the mixture was stirred for 5 min at 0 °C. The Vilsmeier reagent (7.50 mL, 10.7 mmol) was slowly added to a solution of **1** (1.26 g, 4.49 mmol, 2.4 eq) in DMF (15 mL) and the reaction mixture was stirred for 1.5 h at 0 °C. Afterwards, a saturated solution of sodium acetate (50 mL) was added and stirred at RT for 18 h before the mixture was diluted with EtOAc. The phases were separated, the organic layer was washed with water (20 mL) and brine (20 mL), and the aqueous layer was extracted with EtOAc (20 mL). The combined organic layers were dried over Na_2SO_4, concentrated and the crude product was purified by flash chromatography (eluent: DCM → DCM/EtOAc 1:1). The obtained semi-solid was recrystallized from DCM layered with *n*-heptane to yield compound **3** (0.32 g, 0.96 mmol, 21%) as a brown solid.

^{1}H NMR (300 MHz, $CDCl_3$): δ = 10.64 (s, 2H), 9.19 (s, 2H), 8.07 – 7.96 (m, 2H), 7.42 – 7.33 (m, 2H), 6.87 (dd, *J* = 3.9, 2.3 Hz, 2H), 6.08 – 6.00 (m, 2H), 5.63 (s, 1H), 3.91 (s, 3H) ppm.

^{13}C NMR (101 MHz, $CDCl_3$): δ = 178.94, 166.48, 144.06, 140.63, 132.62, 130.07, 129.39, 128.42, 122.09, 111.67, 52.03, 44.22 ppm.

MS (MALDI-TOF, dctb) m/z for $C_{19}H_{17}N_2O_4$: $[M+H]^+$ calculated: 337.12, found: 337.1270

Zinc-5-(4-Methoxycarbonylphenyl)-15-(4-(2-(Trimethylsilyl)ethynyl)phenyl)porphyrin (4)[5]

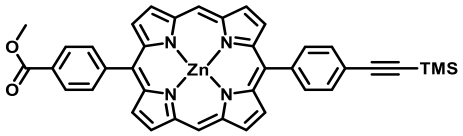

Adapting a literature procedure, **3** (51 mg, 0.15 mmol) was dissolved in THF (0.5 mL), treated with propylamine (0.25 mL, 0.18 g, 3.00 mmol, 20 eq) and stirred for 1 h at RT. The solution was concentrated to dryness and the resulting solid was dissolved in toluene (15 mL). **2** (48 mg, 0.15 mmol, 1 eq) and $Zn(OAc)_2$ (0.28g, 1.5 mmol, 10 eq) were added and the mixture was then refluxed open to air for 18 h. Upon cooling to RT, the crude product was purified by silica gel plug filtration (DCM) and column chromatography (SiO_2, eluent: DCM/hexanes 2:1) to afford compound **4** (14 mg, 21 µmol, 14%) as a purple solid.

^{1}H NMR (400 MHz, THF-d_8): δ = 10.31 (s, 2H), 9.46 – 9.41 (m, 4H), 9.05 – 8.99 (m, 4H), 8.49 – 8.44 (m, 2H), 8.39 – 8.35 (m, 2H), 8.28 – 8.21 (m, 2H), 7.92 – 7.86 (m, 2H), 4.09 (s, 3H), 0.40 (s, 9H) ppm.

^{13}C NMR (101 MHz, THF-d_8): δ = 167.57, 150.81, 150.76, 150.55, 149.35, 144.98, 135.84, 135.73, 132.79, 132.71, 132.65, 132.50, 130.96, 130.46, 128.54, 123.46, 119.77, 119.21, 106.97, 106.58, 95.39, 52.54, 0.34 ppm.

HRMS (MALDI-TOF, dctb) m/z for $C_{39}H_{30}N_4O_2SiZn$: $[M]^+$ calculated: 678.1424, found: 678.1412

UV/Vis (THF): qualitatively (λ_{max}) = 415, 544, 582 nm.

Fluorescence (THF, rt, exc. 415 nm) λ_{max} [nm] (rel. Int. [%]): 589 (100), 638 (97).

Zinc-5-(4-Methoxycarbonylphenyl)-15-(4-(ethynyl)phenyl)porphyrin (5)

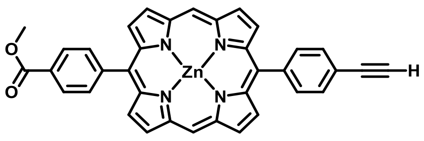

4 (14 mg, 20 µmol) was dissolved in THF (5.0 mL) under N_2-atmosphere. TBAF (22 µL, 22 µmol, 1.1 eq) was added and the solution was stirred for 3 h. Water (0.1 mL) was added slowly before the reaction mixture was diluted with DCM and the phases were separated. The aqueous layer was extracted with DCM (3 x 10 mL) and the combined organic layers were washed with brine (15 mL), dried over Na_2SO_4 and concentrated. The obtained product was dissolved in DCM and precipitated with *n*-pentane to yield compound **5** (11.5 mg, 19 µmol, 95%) as a purple solid.

^{1}H NMR (400 MHz, THF-d_8): δ = 10.28 (s, 2H), 9.45 – 9.40 (m, 4H), 9.01 (dd, *J* = 12.8, 4.4 Hz, 4H), 8.49 – 8.44 (m, 2H), 8.39 – 8.35 (m, 2H), 8.27 – 8.22 (m, 2H), 7.94 – 7.88 (m, 2H), 4.08 (s, 3H), 3.82 (s, 1H) ppm.

^{13}C NMR (101 MHz, THF-d_8): δ = 167.58, 150.80, 150.76, 150.54, 149.46, 145.14, 135.87, 135.80, 132.71, 132.64, 132.44, 131.11, 130.40, 128.50, 122.76, 119.67, 119.15, 106.93, 84.67, 79.87, 52.52 ppm.

HRMS (MALDI-TOF, dctb) m/z for $C_{36}H_{22}N_4O_2Zn$: $[M]^+$ calculated: 606.1029, found: 606.1026

UV/Vis (THF): qualitatively (λ_{max}) = 415, 544, 582 nm.

Fluorescence (THF, rt, exc. 415 nm) λ_{max} [nm] (rel. Int. [%]): 587 (93), 638 (100).

Zinc-5-(4-Carboxylphenyl)-15-(4-(ethynyl)phenyl)porphyrin (6)

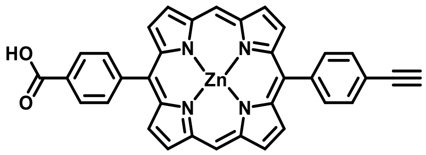

5 (11.5 mg, 19 µmol) was dissolved in THF (4.6 mL) under N_2-atmosphere and treated with LiOH (5.4 mg, 227 µmol, 12 eq) dissolved in water (1.15 mL). The solution was stirred for 22 h before water (3 mL) and HCl (1M, 0.2 mL) were added to acidify the product. The obtained precipitate was filtered and washed with water to afford compound **6** (10.2 mg, 17 µmol, 89%) as a purple solid.

¹H NMR (400 MHz, THF-*d*$_8$): δ = 10.31 (s, 2H), 9.46 – 9.41 (d, *J* = 4.5 Hz, 4H), 9.04 (dd, *J* = 6.0, 4.5 Hz, 4H), 8.50 – 8.46 (m, 2H), 8.38 – 8.33 (m, 2H), 8.27 – 8.23 (m, 2H), 7.94 – 7.90 (m, 2H), 3.82 (s, 1H) ppm.

¹³C NMR (101 MHz, THF-*d*$_8$): δ = 168.18, 150.79, 150.77, 150.75, 150.63, 145.05, 135.78, 135.73, 132.75, 132.70, 132.65, 132.59, 131.14, 128.81, 122.81, 119.67, 119.46, 106.93, 84.66, 79.90 ppm.

HRMS (MALDI-TOF, dctb) m/z for $C_{36}H_{22}N_4O_2Zn$: $[M]^+$ calculated: 592.0872, found: 592.0876

UV/Vis (THF): quantitatively (λ_{max}) = 415, 544, 582 nm.

Fluorescence (THF, rt, exc. 415 nm) λ_{max} [nm] (rel. Int. [%]): 589 (91), 638 (100).

G2-Newkome-zinc-AB$_2$C-acetylene porphyrin (7)

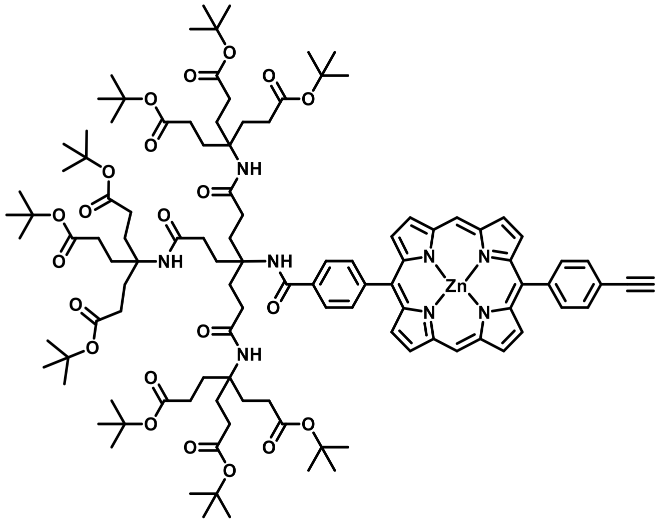

6 (4.9 mg, 8.2 µmol), Newkome G2 (18 mg, 12 µmol, 1.5 eq) and HOBt·H_2O (1.9 mg, 11 µmol, 1.3 eq) were dissolved in DMF (5.0 mL). DCC (2.2 mg, 11 µmol, 1.3 eq) dissolved in DMF (2.5 mL) was added and stirred for 5 d at rt. The reaction mixture was concentrated under reduced pressure and crude product was purified by silica gel plug filtration (EtOAc) and subsequent column chromatography (SiO_2, eluent: toluene/THF 5:1 v/v) to afford compound **7** (7.5 mg, 3.7 µmol, 45%) as a purple solid.

¹H NMR (400 MHz, THF-*d*$_8$): δ = 10.29 (s, 2H), 9.45 – 9.40 (m, 4H), 9.05 (dd, *J* = 14.5, 4.5 Hz, 4H), 8.91 (s, 1H), 8.45 – 8.40 (m, 2H), 8.33 – 8.29 (m, 2H), 8.26 – 8.22 (m, 2H,), 7.93 – 7.89 (m, 2H), 6.90 (s, 3H,), 3.81 (s, 1H), 2.47 – 2.40 (m, 6H), 2.35 – 2.28 (m, 6H), 2.27 – 2.19 (m, 18H), 2.05 – 1.95 (m, 18H), 1.41 (s, 81H) ppm.

^{13}C NMR (126 MHz, THF-d_8): δ = 173.89, 173.17, 166.91, 157.42, 150.93, 150.77, 150.70, 150.67, 146.87, 145.12, 135.88, 135.77, 135.55, 132.98, 132.61, 132.58, 131.12, 126.72, 122.77, 120.11, 119.46, 106.79, 84.66, 80.29, 79.86, 59.18, 58.12, 32.35, 32.14, 30.60, 30.40, 28.54 ppm.

HRMS (MALDI-TOF, dctb) m/z for $C_{36}H_{22}N_4O_2Zn$: $[M]^+$ calculated: 2013.0307, found: 2013.0305

UV/Vis (THF): quantitatively (λ_{max}) = 415, 544, 581 nm.

Fluorescence (THF, rt, exc. 415 nm) λ_{max} [nm] (rel. Int. [%]): 587 (85), 639 (100).

***t*-Bu-G2-mesityl-zinc-porphyrin-*t*-Bu-phenoxylperyenebisimide Dyad (11)**

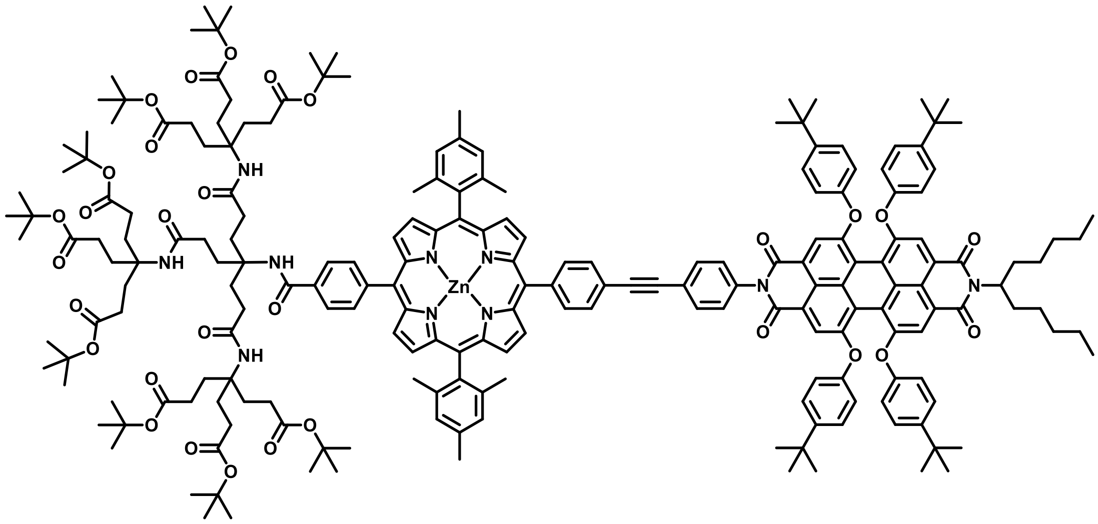

A microwave vial was charged with G2-mesityl Porphyrin **8** (27 mg, 12 µmol, 1.2 eq), tBu phenoxy PBI **9** (13.4 mg, 10 µmol, 1 eq.), CuI (0.4 mg, 2.2 µmol), $Pd(PPh_3)_4$ (1.3 mg, 1.1 µmol) and PPh_3 (0.6 mg, 2.2 µmol) the vial was sealed and then evacuated and refilled with nitrogen three times. Subsequently, an externally degassed mixture of THF/NEt_3 (2 mL / 1 mL) was added and the reaction mixture was stirred at 85 °C for 20 h. Then, the mixture was allowed to cool to room temperature and then filtered through a plug of silica gel (eluent: Tol / THF 5:1 v/v). The resulting crude product was further purified by gel permeation size exclusion chromatography ($CHCl_3$ Ø 2.5 cm, ↑ 60 cm) yielding the desired dyad as a dark purple solid (29 mg, 8.37 µmol, 84 %)

^{1}H NMR (500 MHz, THF-*d*$_8$) δ = 8.92 (s, 1H), 8.84 – 8.80 (m, 4H), 8.70 – 8.66 (m, 4H), 8.40 – 8.34 (m, 2H), 8.27 – 8.21 (m, 4H), 8.20 (s, 2H), 8.14 – 8.07 (m, 2H), 7.94 – 7.90 (m, 2H), 7.77 – 7.73 (m, 2H), 7.40 – 7.36 (m, 2H), 7.35 – 7.27 (m, 12H), 6.95 – 6.86 (m, 8H), 6.84 (s, 3H), 5.13 (tt, *J*=10.1, 5.5, 1H), 2.61 (s, 6H), 2.43 – 2.37 (m, 6H), 2.30 – 2.24 (m, 6H), 2.24 – 2.18 (m, 18H), 2.00 – 1.94 (m, 18H), 1.85 (s, 12H), 1.39 (s, 81H), 1.34 – 1.27 (m, 54H), 0.91 – 0.87 (m, 6H).

^{13}C NMR (126 MHz, THF-*d*$_8$) δ = 173.84, 173.14, 166.74, 163.49, 154.56, 150.87, 150.81, 150.65, 148.27, 148.11, 146.98, 145.05, 140.86, 140.03, 138.23, 137.13, 135.64, 135.26, 134.24, 132.93, 131.11, 131.05, 130.57, 130.46, 128.68, 127.70, 127.67, 126.58, 124.20, 123.34, 120.88, 120.73, 120.70, 120.41, 120.15, 120.00, 119.64, 80.25, 59.12, 58.07, 55.24, 35.24, 33.37, 33.00, 32.92, 32.28, 32.08, 31.97, 30.60, 30.38, 30.14, 28.54, 27.59, 23.73, 23.61, 22.16, 21.75, 14.59 *(The sp carbons of the tolane linker are not observed due to their low intensity)*

HRMS (MALDI-TOF, dctb) m/z for $C_{210}H_{254}N_{10}O_{30}Zn$: $[M]^+$ calculated: 3459.7943, found: 3459.7947

IR (ATR, cm^{-1}): $\tilde{\nu}$=: 2970, 1729, 1699, 1661, 1585, 1504, 1455, 1409, 1393, 1366, 1338, 1288, 1213, 1148, 995, 881

UV/Vis (THF): λ_{max}(ε[mol*$L^{-1}cm^{-1}$]) = 287 (95000), 426 (582000), 529 (37000), 562 (66000) nm.

Fluorescence (THF, rt, exc. 425 nm) λ_{max} [nm] (rel. Int. [%]): 604 (100), 655 (55).

***t*-Bu-G2-mesityl-zinc-porphyrin-perylenebisimide Dyad (12)**

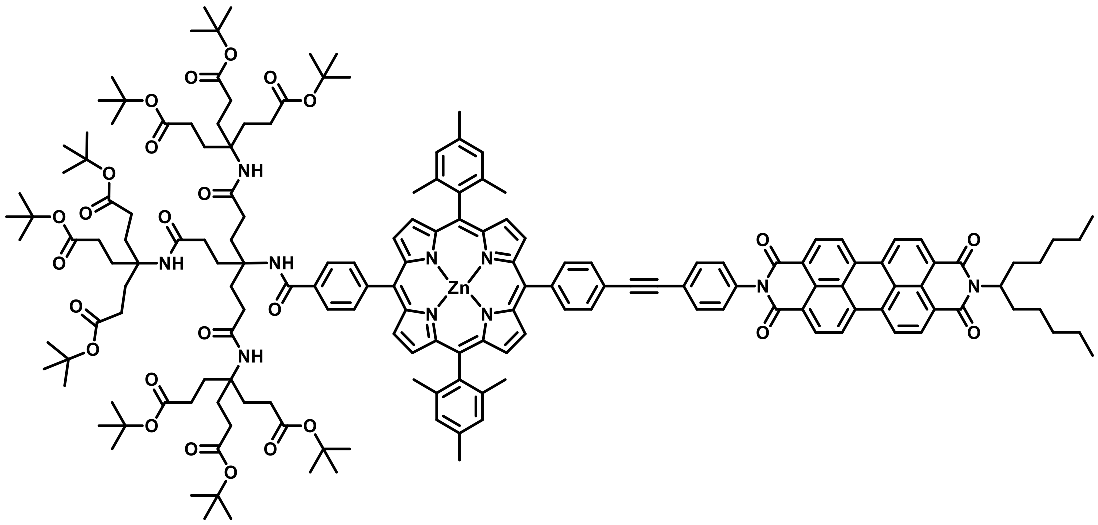

A microwave vial was charged with G2-mesityl Porphyrin **8** (27 mg, 12 µmol, 1.2 eq), Iodo-phenyl perylenebisimide **10** (7.5 mg, 10 µmol, 1 eq.), CuI (0.4 mg, 2.2 µmol), $Pd(PPh_3)_4$ (1.3 mg, 1.1 µmol) and PPh_3 (0.6 mg, 2.2 µmol) the vial was sealed and then evacuated and refilled with nitrogen three times. Subsequently, an externally degassed mixture of THF/NEt_3 (2 mL / 1 mL) was added and the reaction mixture was stirred at 85 °C for 20 h. Then, the mixture was allowed to cool to room temperature and then filtered through a plug of silica gel (eluent: Tol / THF 5:1 v/v). The resulting crude product was further purified by gel permeation size exclusion chromatography ($CHCl_3$ Ø 2.5 cm, ↑ 60 cm) yielding the desired dyad as a dark purple solid (17 mg, 5.92 µmol, 59 %)

^{1}H NMR (600 MHz, THF-d_8) δ = 8.92 (s, 1H), 8.87 – 8.81 (m, 4H), 8.73 – 8.62 (m, 8H), 8.58 – 8.50 (m, 5H), 8.41 – 8.34 (m, 2H), 8.29 – 8.24 (m, 4H), 8.01 – 7.95 (m, 2H), 7.88 – 7.83 (m, 2H), 7.58 – 7.54 (m, 2H), 7.30 (s, 4H), 6.87 (s, 3H), 5.22 (tt, *J*=9.2, 5.9, 1H), 2.61 (s, 6H), 2.44 – 2.39 (m, 6H), 2.31 – 2.25 (m, 6H), 2.24 – 2.18 (m, 18H), 2.02 – 1.95 (m, 18H), 1.86 (s, 12H), 1.39 (s, 81H), 1.36 – 1.26 (m, 16H), 0.88 (t, *J*=7.1, 6H).

^{13}C NMR (151 MHz, THF-d_8) δ = 173.87, 173.15, 166.77, 163.77, 150.87, 150.82, 150.68, 146.99, 145.06, 140.87, 140.03, 138.24, 137.22, 136.21, 135.82, 135.67, 135.61, 135.26, 133.09, 132.99, 132.71, 131.85, 131.12, 131.06, 130.67, 130.63, 130.52, 130.33, 128.68, 127.30, 127.16, 126.59, 124.69, 124.53, 124.47, 123.42, 120.69, 120.18, 119.65, 91.08, 90.80,

80.26, 59.12, 58.06, 55.24, 33.39, 32.96, 32.27, 32.09, 30.57, 30.37, 28.54, 27.76, 23.66, 22.18, 21.76, 14.62

HRMS (MALDI-TOF, dctb) m/z for $C_{170}H_{206}N_{10}O_{26}Zn$: $[M]^+$ calculated: 2867.4391, found: 2867.4361

IR (ATR, cm^{-1}): $\tilde{\nu}$=: 2923, 1728, 1658, 1594, 1522, 1455, 1367, 1341, 1254, 1150, 1101, 996, 848. 810, 797, 747, 720, 619

UV/Vis (THF): λ_{max}(ε[mol*L^{-1}cm^{-1}]) = 426 (628000), 486 (64000), 522 (100000), 557 (30000), 597 (12000) nm.

Fluorescence (THF, rt, exc. 425 nm) λ_{max} [nm] (rel. Int. [%]): 605 (100), 656 (73).

***t*-Bu-G2-zinc-porphyrin-perylenebisimide Dyad (13)**

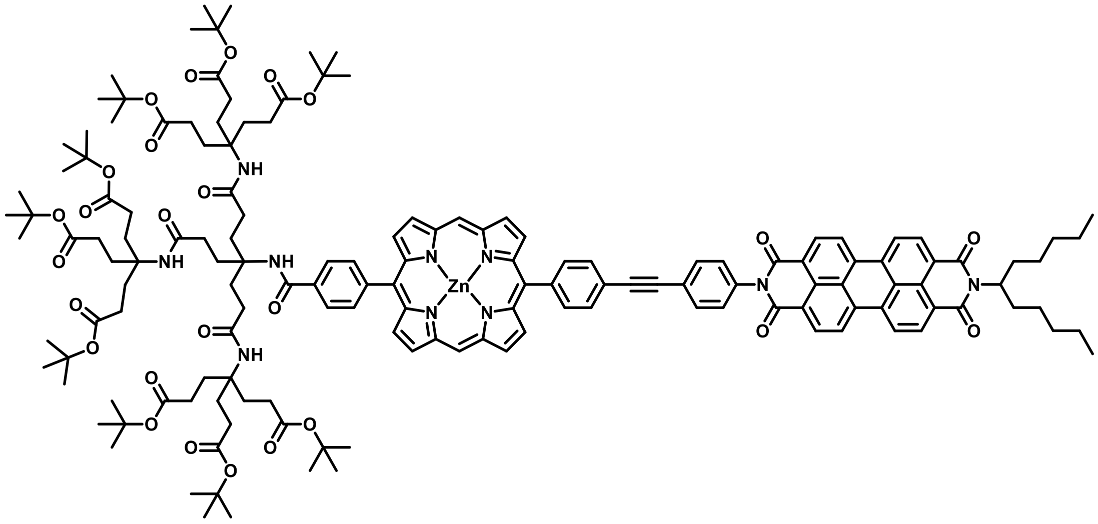

A microwave vial was charged with G2- Porphyrin **7** (15 mg, 7.4 µmol, 1.1 eq), Iodo-phenyl perylenebisimide **10** (5.0 mg, 6.76 µmol, 1 eq.), CuI (0.4 mg, 2.2 µmol), $Pd(PPh_3)_4$ (1.3 mg, 1.1 µmol) and PPh_3 (0.6 mg, 2.2 µmol) the vial was sealed and then evacuated and refilled with nitrogen three times. Subsequently, an externally degassed mixture of THF/NEt_3 (2 mL / 1 mL) was added and the reaction mixture was stirred at 85 °C for 20 h. Then, the mixture was allowed to cool to room temperature and then filtered through a plug of silica gel (eluent: Tol / THF 5:1 v/v). The resulting crude product was further purified by gel permeation size exclusion chromatography ($CHCl_3$ Ø 2.5 cm, ↑ 60 cm) yielding the desired dyad as a dark purple solid (9.3 mg, 3.53 µmol, 52 %)

^{1}H NMR (500 MHz, THF-d_8) δ = 10.25 (s, 2H), 9.47 – 9.36 (m, 4H), 9.13 – 9.05 (m, 4H), 8.89 (s, 1H), 8.55 – 8.40 (m, 10H), 8.33 (dd, *J*=7.9, 4.8, 4H), 8.09 – 8.05 (m, 2H), 7.94 – 7.89 (m, 2H), 7.63 – 7.57 (m, 2H), 6.92 (s, 3H), 5.24 (td, *J*=9.1, 4.6, 1H), 2.44 (d, *J*=7.3, 6H), 2.35 – 2.30 (m, 6H), 2.27 – 2.21 (m, 18H), 2.06 – 1.98 (m, 18H), 1.42 (s, 81H), 1.37 – 1.27 (m, 16H), 0.90 (t, 6H).

^{13}C NMR (126 MHz, THF-d_8) δ = 173.91, 173.19, 166.95, 163.74, 150.89, 150.71, 150.67, 146.86, 144.92, 136.01, 135.59, 135.25, 134.95, 133.05, 132.65, 132.58, 131.62, 130.83, 130.71, 130.24, 130.18, 126.88, 126.75, 124.41, 124.29, 124.16, 123.46, 120.07, 119.59, 106.79, 91.12, 90.88, 80.30, 59.19, 58.14, 55.20, 35.04, 33.41, 32.98, 32.38, 32.16, 30.60, 30.41, 28.55, 27.78, 23.69, 14.64.

HRMS (MALDI-TOF, dctb) m/z for $C_{152}H_{186}N_{10}O_{26}Zn$: $[M]^+$ calculated: 2631.2826, found: 2631.2841

IR (ATR, cm^{-1}): $\tilde{v}$=: 2924, 1727, 1657, 1594, 1519, 1454, 1393, 1367, 1341, 1254, 1148, 1101, 1061, 991

UV/Vis (THF): λ_{max} (ε[mol*L^{-1}cm^{-1}]) = 415 (476000), 455 (29000) 487 (61000), 523 (95000), 544 (31000), 582 (9600) nm.

Fluorescence (THF, rt, exc. 415 nm) λ_{max} [nm] (rel. Int. [%]): 590 (85), 638 (95).

Carboxylic acid-G2-mesityl-zinc-porphyrin-*t*-Bu-phenoxyperylenebisimide Dyad (14)

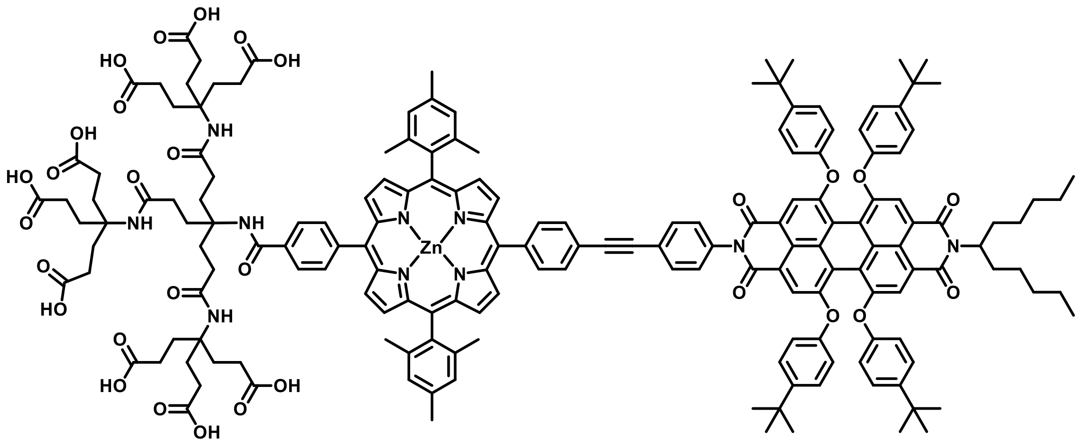

In a 10 mL microwave vial, *t*Bu-G2-dendron dyad **11** (15 mg, 4.3 µmol) was dissolved in formic acid (4 mL) under a nitrogen atmosphere. The reaction mixture was stirred under the exclusion of light at room temperature for 3 d. Then, the solvent was removed azeotropically with toluene (3x 15 mL) under reduced pressure. The remaining solid was redissolved in THF (5 mL) and $Zn(OAc)_2$ (3.2 mg, 17.3 µmol, 4 eq.) was added and the mixture was stirred at 55 °C for 12 h to re-metalate the dyad. When the dyad was fully metalated as judged by UV/Vis spectroscopy, the mixture was slightly acidified with a few drops of acetic acid and heptane was added to precipitate the dyad. The precipitate was collected by filtration and washed with H_2O (3x 5 mL) and acetone (1x 5 mL), yielding the desired amphiphile **14** as a dark purple solid (11.8 mg, 4.0 µmol, 93 %)

^{1}H NMR (400 MHz, THF-d_8 / 1 vol% TFA-d) δ = 8.84 – 8.80 (m, 4H), 8.78 (s, 1H), 8.67 (d, *J*=4.6, 4H), 8.40 – 8.34 (m, 2H), 8.26 – 8.21 (m, 4H), 8.19 (s, 2H), 8.15 – 8.08 (m, 2H), 7.95 – 7.90 (m, 2H), 7.78 – 7.73 (m, 2H), 7.40 – 7.28 (m, 14H), 6.97 (s, 3H), 6.94 – 6.87 (m, 8H), 5.17 – 5.07 (m, 1H), 2.60 (s, 6H), 2.45 – 2.37 (m, 6H), 2.32 – 2.22 (m, 26H), 2.10 – 2.00 (m, 20H), 1.84 (s, 12H), 1.32 (s, 18H), 1.30 (s, 18H), 1.27 – 1.21 (m, 12H), 0.84 – 0.80 (m, 6H).

^{13}C NMR *Due to the instability of the molecule towards the acidic conditions over the prolonged times required for the measurement, we were not able to acquire a suitable carbon NMR spectrum.*

HRMS (MALDI-TOF, dctb) m/z for $C_{174}H_{182}N_{10}NaO_{30}Zn$: $[M]^+$ calculated: 2955.2309, found: 2955.2397

IR (ATR, cm^{-1}): $\tilde{\nu}$=: 3306, 2961, 1699, 1663, 1586, 1505, 1410, 1339, 1300, 1201, 1148, 997, 879, 832

UV/Vis (THF/10 mM NaOH (aq) 1:1 v/v): λ_{max} (ε[mol*L^{-1}cm^{-1}]) = 426 (471000), 530 (28000), 562 (50000) nm.

Fluorescence (THF/10 mM NaOH (aq) 1:1 v/v, rt, exc. 425 nm) λ_{max} [nm] (rel. Int. [%]): 605 (100), 654 (51).

Carboxylic acid-G2-mesityl-zinc-porphyrin-perylenebisimide Dyad (15)

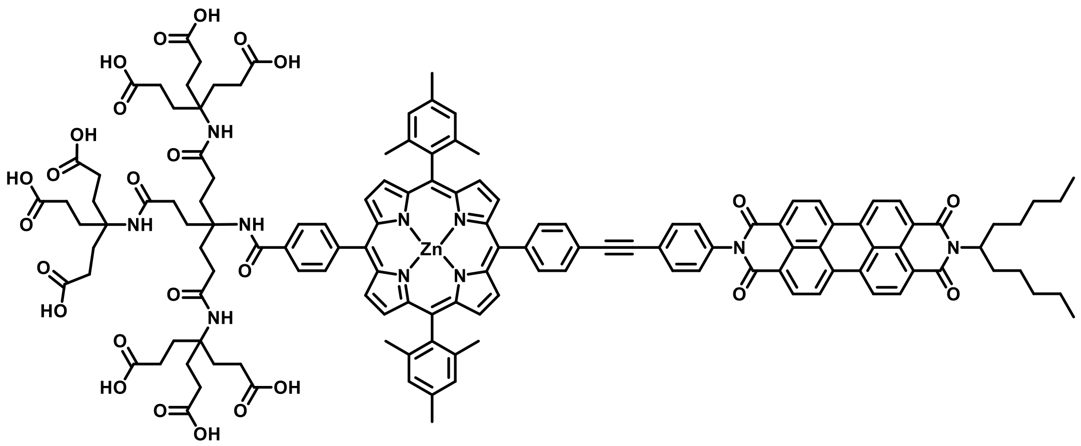

In a 10 mL microwave vial, *t*Bu-G2-dendron dyad **12** (9.0 mg, 3.1 µmol) was dissolved in formic acid (3 mL) under a nitrogen atmosphere. The reaction mixture was stirred under the exclusion of light at room temperature for 3 d. Then, the solvent was removed azeotropically with toluene (3x 15 mL) under reduced pressure. The remaining solid was redissolved in THF (5 mL) and $Zn(OAc)_2$ (4.8 mg, 25.0 µmol, 8 eq.) was added and the mixture was stirred at 55 °C for 12 h to re-metalate the dyad. When the dyad was fully metalated as judged by UV/Vis spectroscopy,

the mixture was slightly acidified with a few drops of acetic acid and heptane was added to precipitate the protonated dyad. The precipitate was collected by filtration and washed with H_2O (3x 5 mL) and acetone (1x 5 mL), yielding the desired amphiphile **15** as a dark red solid (7.3 mg, 3.1 µmol, 99 %)

^{1}H NMR (400 MHz, THF-d_8 / 1 vol% TFA-d)) δ = 8.89 – 8.81 (m, 8H), 8.80 (s, 1H), 8.71 – 8.66 (m, 4H), 8.66 – 8.60 (m, 4H), 8.38 (d, *J*=7.9, 2H), 8.28 – 8.22 (m, 4H), 7.98 – 7.94 (m, 2H), 7.85 – 7.81 (m, 2H), 7.51 – 7.48 (m, 2H, *partially overlayed by the TFA signal*), 6.95 (s, 3H), 5.27 – 5.18 (m, 1H), 2.60 (s, 6H), 2.45 – 2.38 (m, 6H), 2.32 – 2.22 (m, 26H), 2.10 – 2.00 (m, 20H), 1.85 (s, 12H), 1.39 – 1.26 (m, 12H), 0.88 – 0.83 (m, 6H).

The signal of the mesityl group is overlayed by the TFA signal and cannot be observed

^{13}C NMR *Due to the instability of the molecule towards the acidic conditions over the prolonged times required for the measurement, we were not able to acquire a suitable carbon NMR spectrum.*

HRMS (ESI-TOF, positive mode) m/z for $C_{134}H_{134}N_{10}NaO_{26}Zn$: $[M+Na]^+$ calculated: 2385.8654, found: 2385.8649

IR (ATR, cm^{-1}): $\tilde{\nu}$=: 3290, 2957, 1698, 1557, 1405¸1342¸1255, 1203, 1176, 1105, 1051, 1025, 996, 960

UV/Vis (THF/10 mM NaOH (aq) 1:1 v/v): λ_{max} (ε[mol*L^{-1}cm^{-1}]) = 426 (334000), 490 (32000) 525 (45000), 557 (16000) 600 (5600) nm.

Fluorescence (THF/10 mM NaOH (aq), rt, exc. 425 nm) λ_{max} [nm] (rel. Int. [%]): 607 (100), 655 (80).

Carboxylic acid-G2-zinc-porphyrin-perylenebisimide Dyad (16)

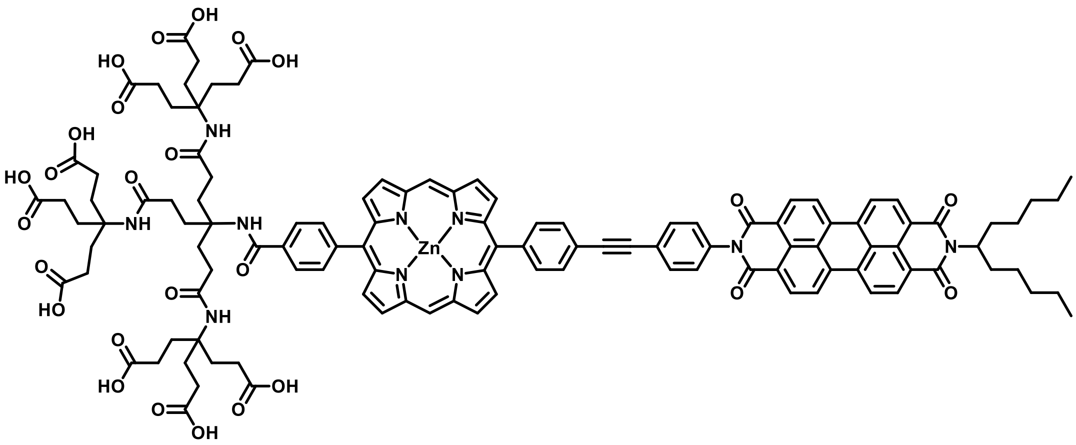

In a 10 mL microwave vial, *t*Bu-G2-dendron dyad **13** (6.0 mg, 2.3 µmol) was dissolved in formic acid (2 mL) under a nitrogen atmosphere. The reaction mixture was stirred under the exclusion of light at room temperature for 3 d. Then, the solvent was removed azeotropically with toluene

(3x 15 mL) under reduced pressure. The remaining solid was redissolved in THF (5 mL) and $Zn(OAc)_2$ (1.6 mg, 9.1 µmol,4 eq.) was added and the mixture was stirred at 55 °C for 12 h to re-metalate the dyad. When the dyad was fully metalated as judged by UV/Vis spectroscopy, the mixture was slightly acidified with a few drops of acetic acid and heptane was added to precipitate the protonated dyad. The precipitate was collected by filtration and washed with H_2O (3x 5 mL) and acetone (1x 5 mL), yielding the desired amphiphile **16** as a dark red solid (3.4 mg, 1.6 µmol, 70 %)

^{1}H NMR (400 MHz, THF-d_8 / 1 vol% TFA-d)) δ = 10.22 (s, 2H), 9.46 – 9.34 (m, 4H), 9.07 (dd, *J*=10.3, 4.4, 4H), 8.89 (s, 1H), 8.71 – 8.50 (m, 8H), 8.48 – 8.40 (m, 2H), 8.36 – 8.27 (m, 4H), 8.09 – 8.01 (m, 2H), 7.93 – 7.85 (m, 2H), 7.59 – 7.53 (m, 2H), 6.99 (s, 3H), 5.29 – 5.19 (m, 1H), 2.52 – 2.42 (m, 6H), 2.39 – 2.18 (m, 26H), 2.16 – 1.96 (m, 18H), 1.45 – 1.21 (m, 12H), 0.88 (t, *J*=7.2, 6H).

^{13}C NMR *Due to the instability of the molecule towards the acidic conditions over the prolonged times required for the measurement, we were not able to acquire a suitable carbon NMR spectrum.*

HRMS (ESI-TOF, positive mode) m/z for $C_{116}H_{114}N_{10}NaO_{26}Zn$: $[M+Na]^+$ calculated: 2149.7089, found: 2149.7091

IR (ATR, cm^{-1}): $\tilde{\nu}$=: 3289, 2922, 1699, 1653, 1593, 1548, 1404, 1342, 1254, 1196, 1148, 1103, 1060, 995, 851, 809

UV/Vis (THF/10 mM NaOH (aq) 1:1 v/v): λ_{max} (ε[mol*L^{-1}cm^{-1}]) = 415 (329000), 458 (19000) 489 (42000), 526 (65000) 581 (7400) nm.

Fluorescence (THF/10 mM NaOH (aq), rt, exc. 415 nm) λ_{max} [nm] (rel. Int. [%]): 589 (100), 637 (90).

3. NMR Spectra

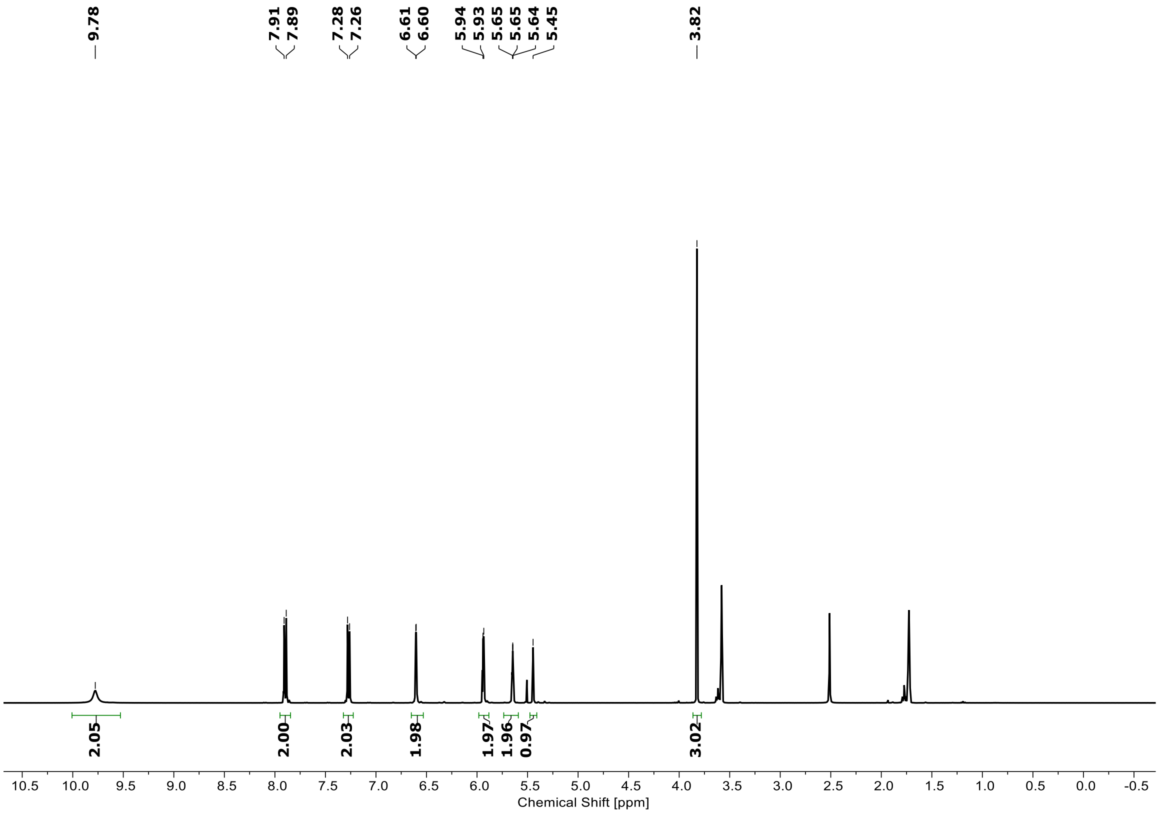

Figure S1 ^{1}H NMR (400 MHz, THF-d_8) of compound **1** with residual water and DCM.

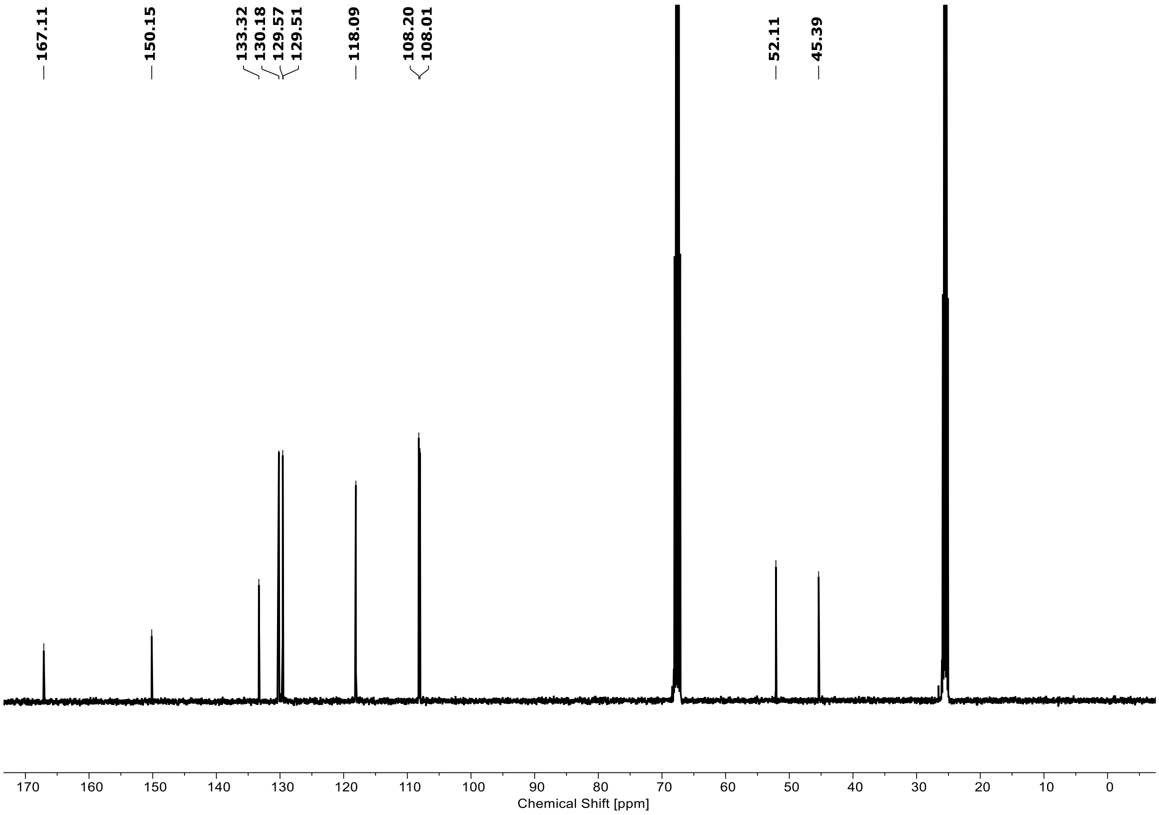

Figure S2 ^{13}C NMR (101 MHz, THF-d_8) of compound **1**.

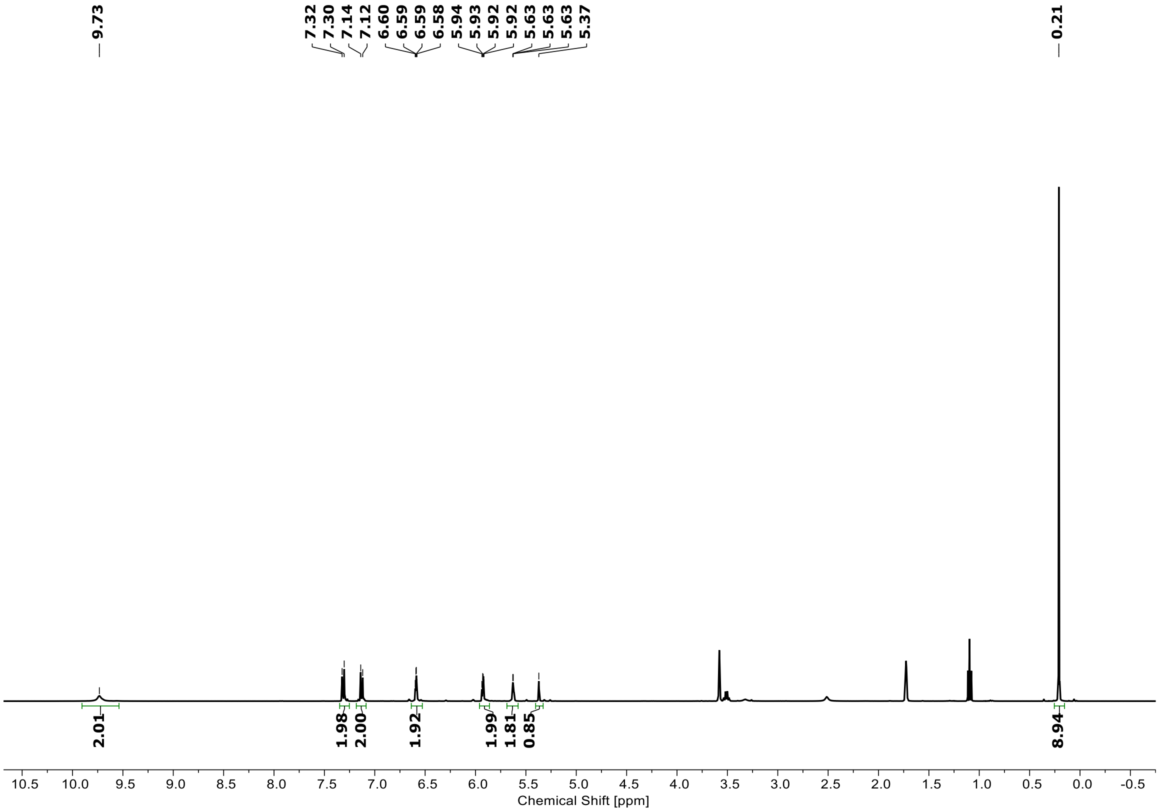

Figure S3 ^{1}H NMR (400 MHz, THF-*d*$_{8}$) of compound **2** with residual water and EtOH.

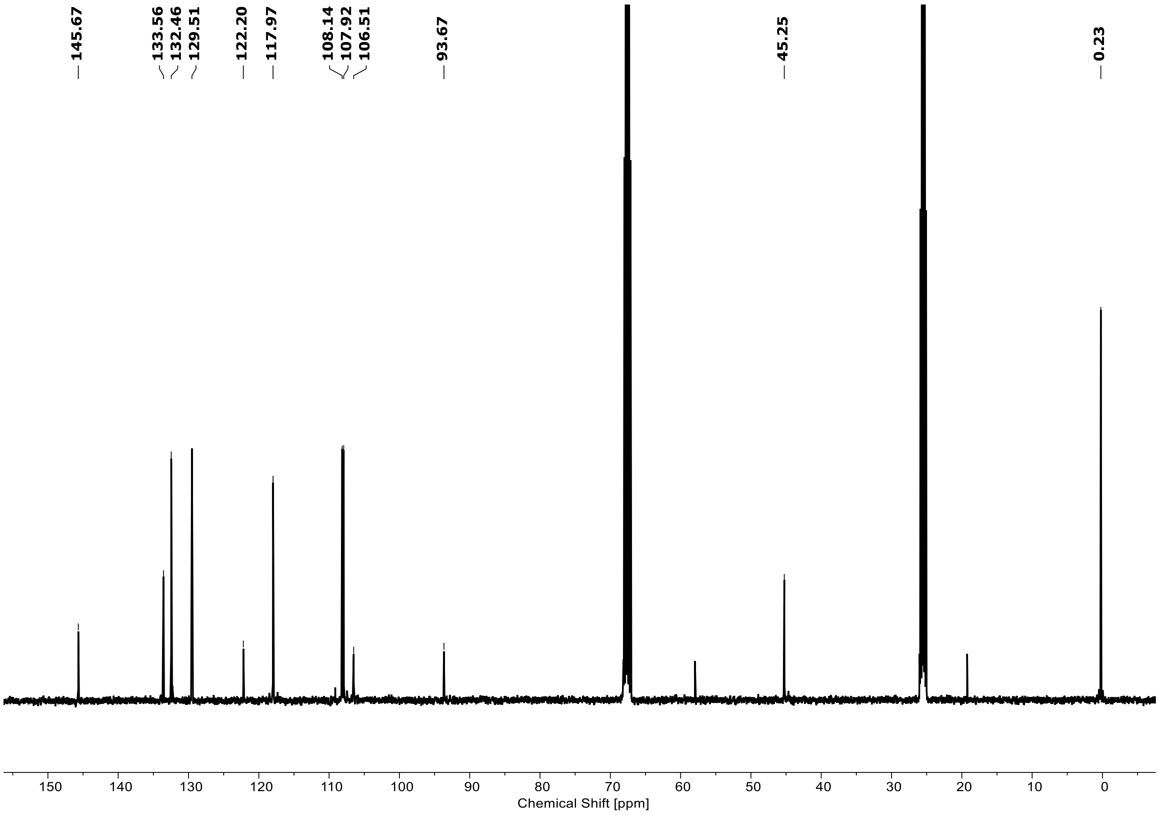

Figure S4 ^{13}C NMR (101 MHz, THF-d8) of compound **2**.

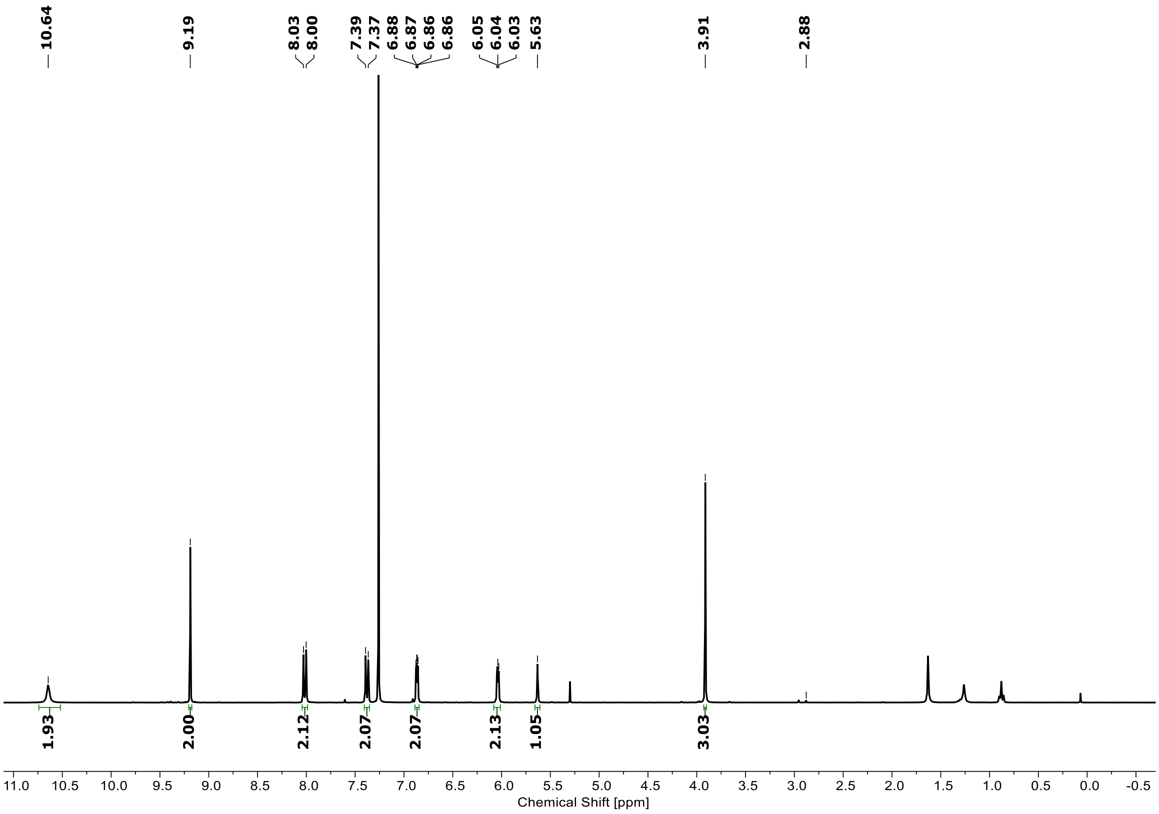

Figure S5 ^{1}H NMR (300 MHz, $CDCl_3$) of compound **3** with residual DCM, DMF, water, n- heptane and silicone grease.

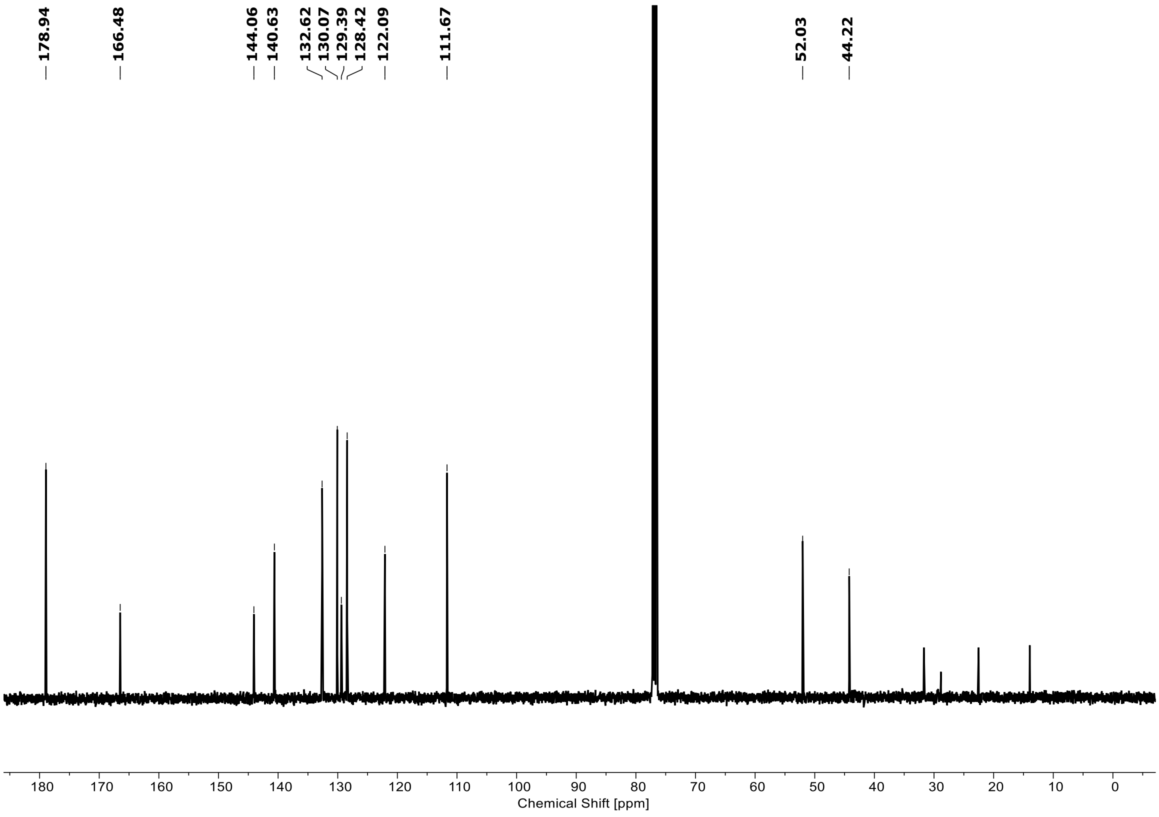

Figure S6 ^{13}C NMR (101 MHz, $CDCl_3$) of compound **3.**

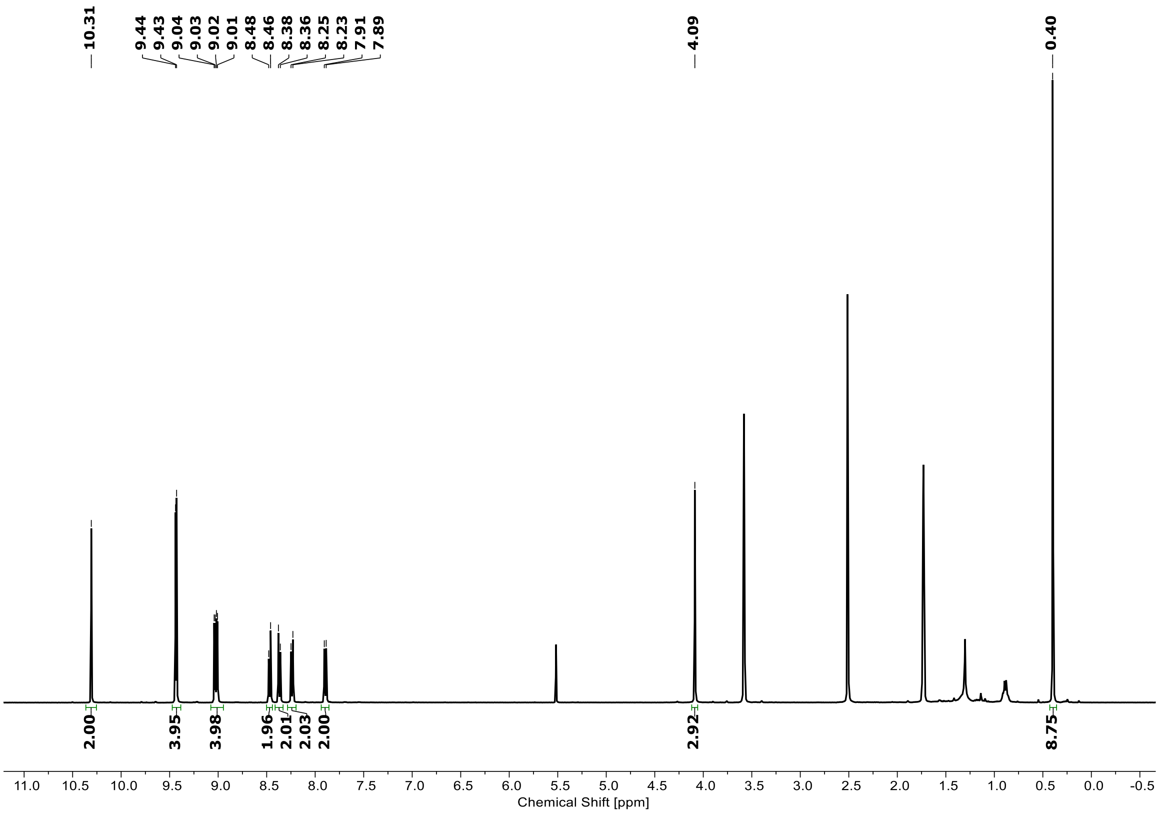

Figure S7 ^{1}H NMR (400 MHz, THF-d_8) of compound **4** with residual DCM, water and hexanes.

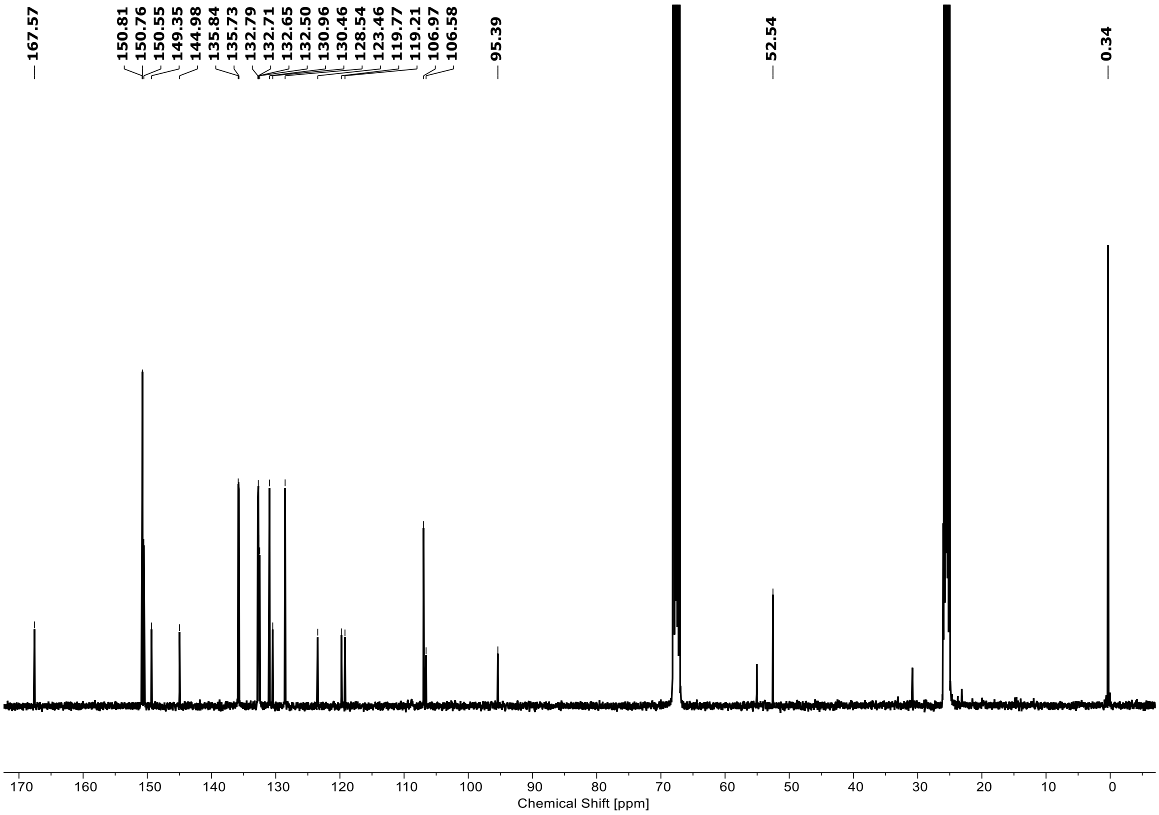

Figure S8 ^{13}C NMR (101 MHz, THF-d_8) of compound **4.**

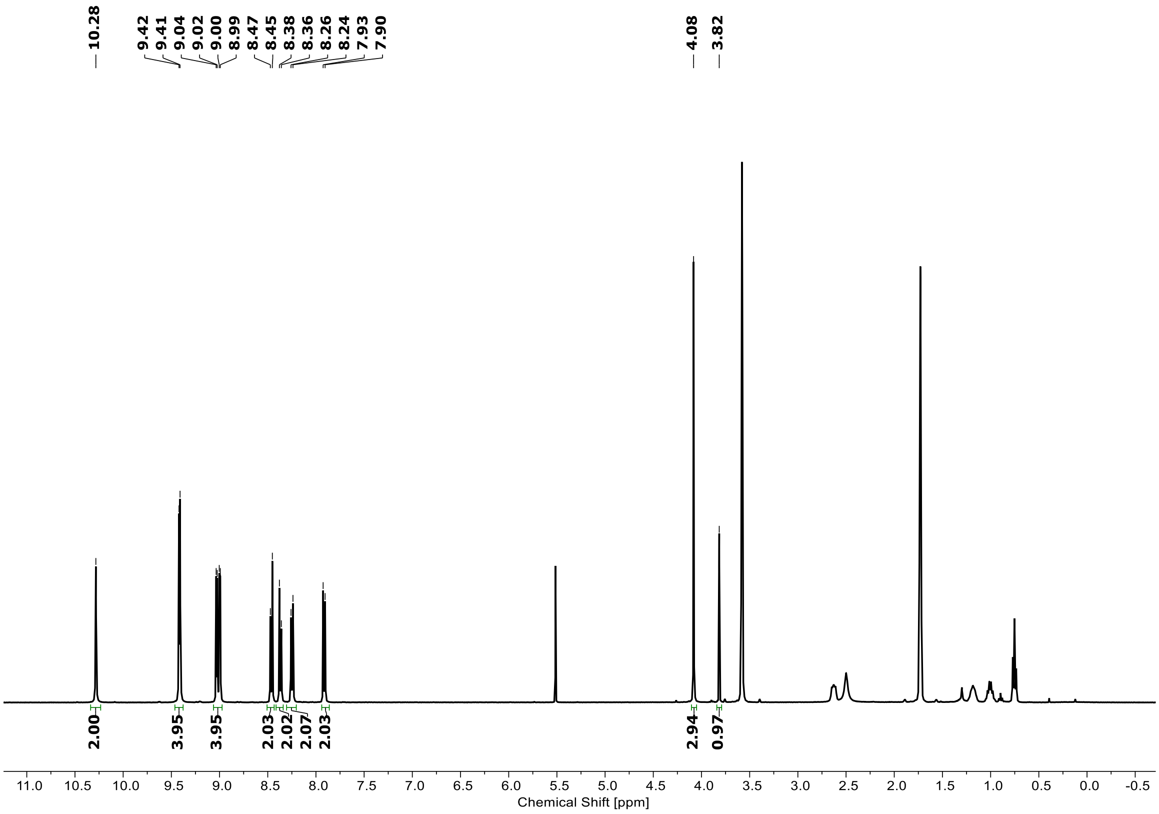

Figure S9 ^{1}H NMR (400 MHz, THF-d_8) of compound **5** with residual DCM, water and n-pentane.

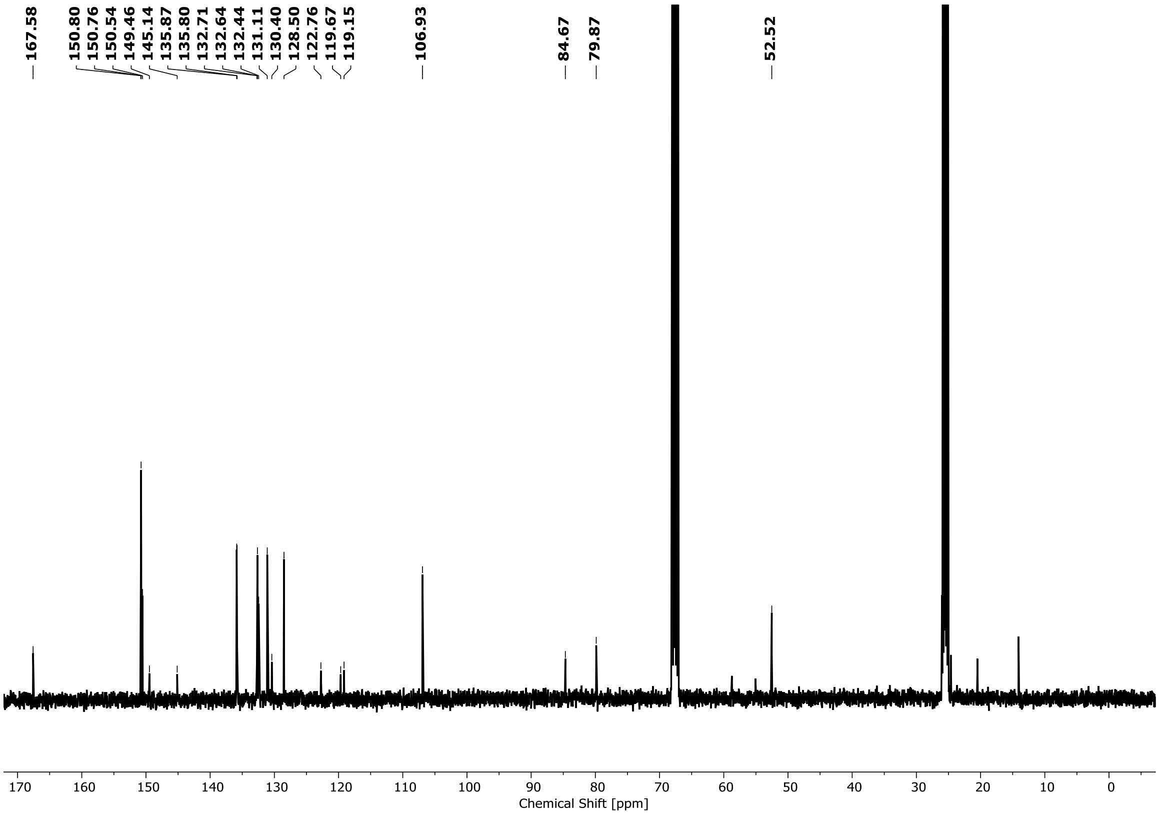

Figure S10 ^{13}C NMR (101 MHz, THF-d_8) of compound **5.**

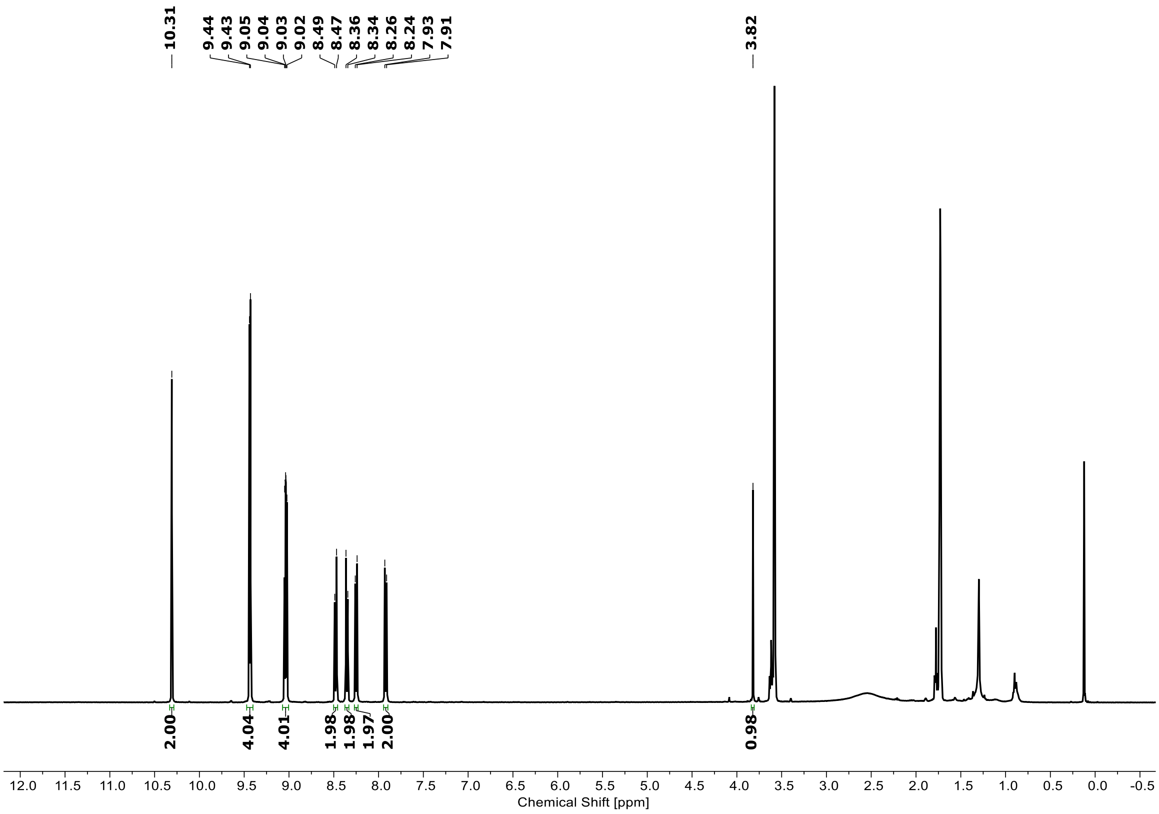

Figure S11 ^{1}H NMR (400 MHz, THF-d_8) of compound **6** with residual water, n-heptane, and silicone grease.

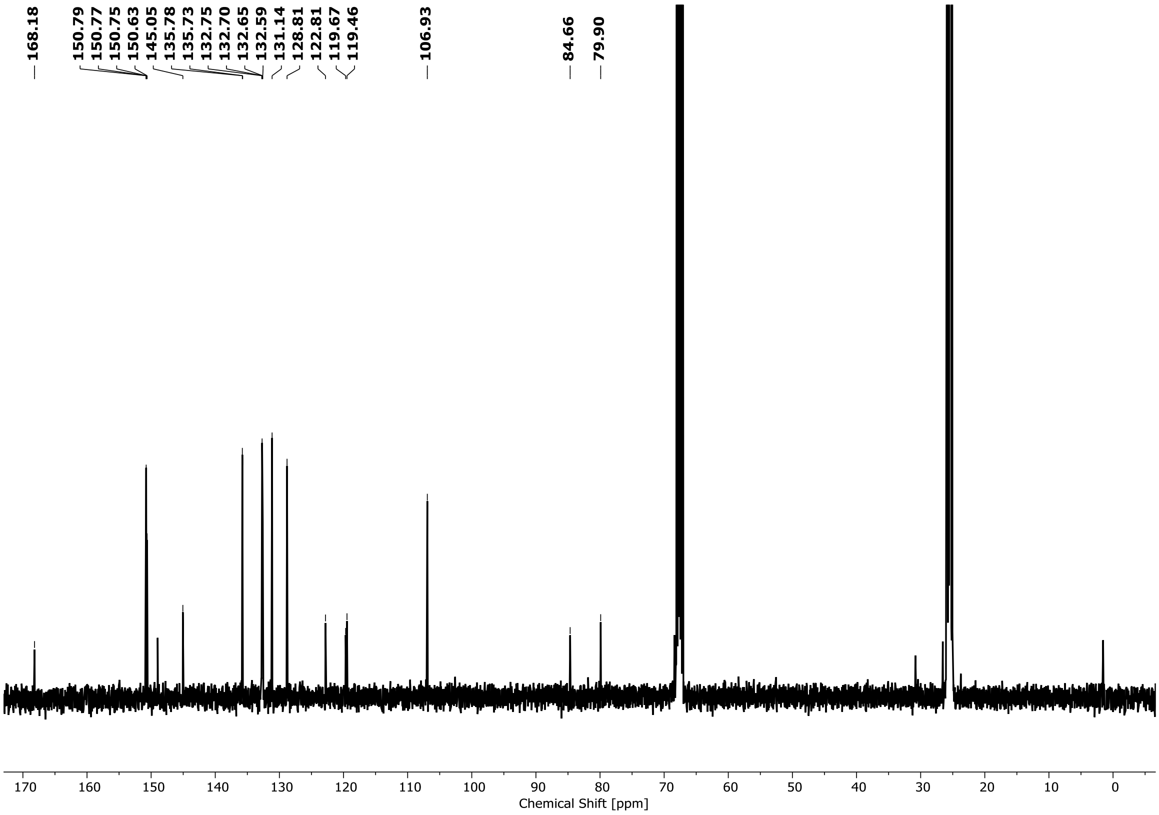

Figure S12 ^{13}C NMR (101 MHz, THF-d_8) of compound **6.**

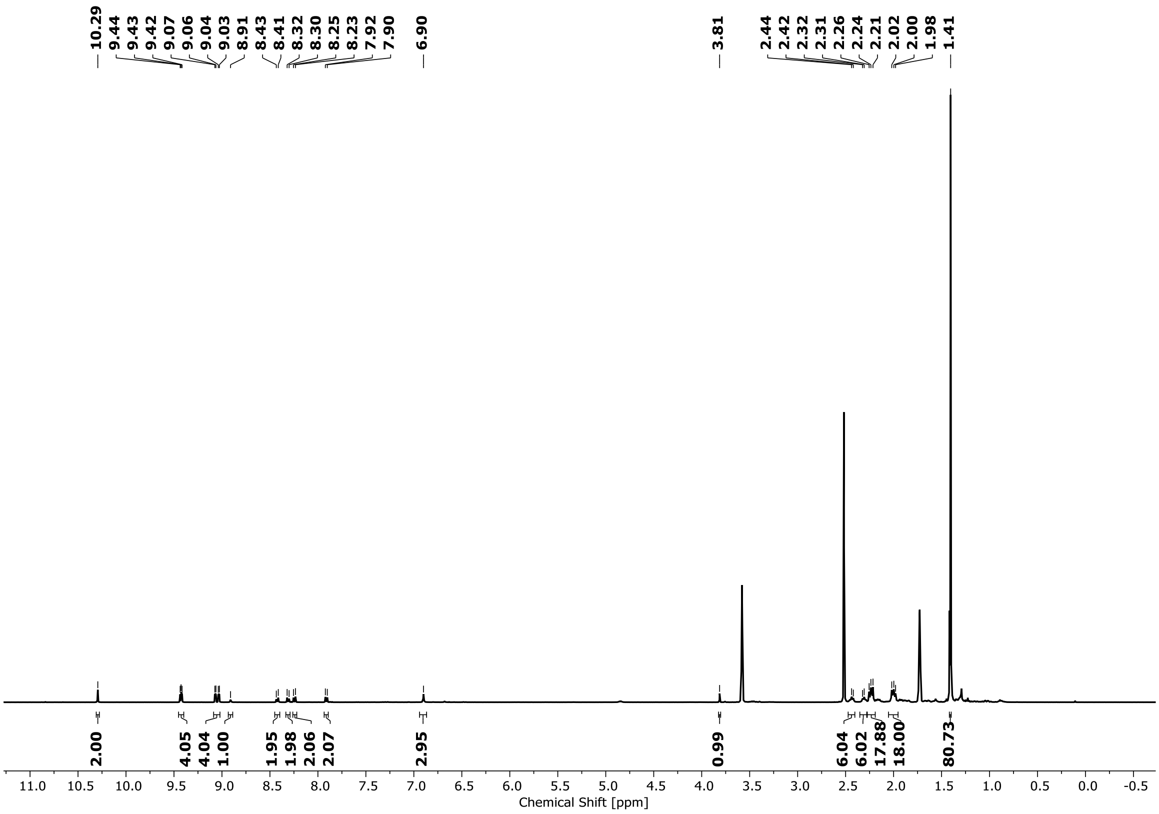

Figure S13 ^{1}H NMR (400 MHz, THF-d_8) of compound **7** with residual water and silicone grease.

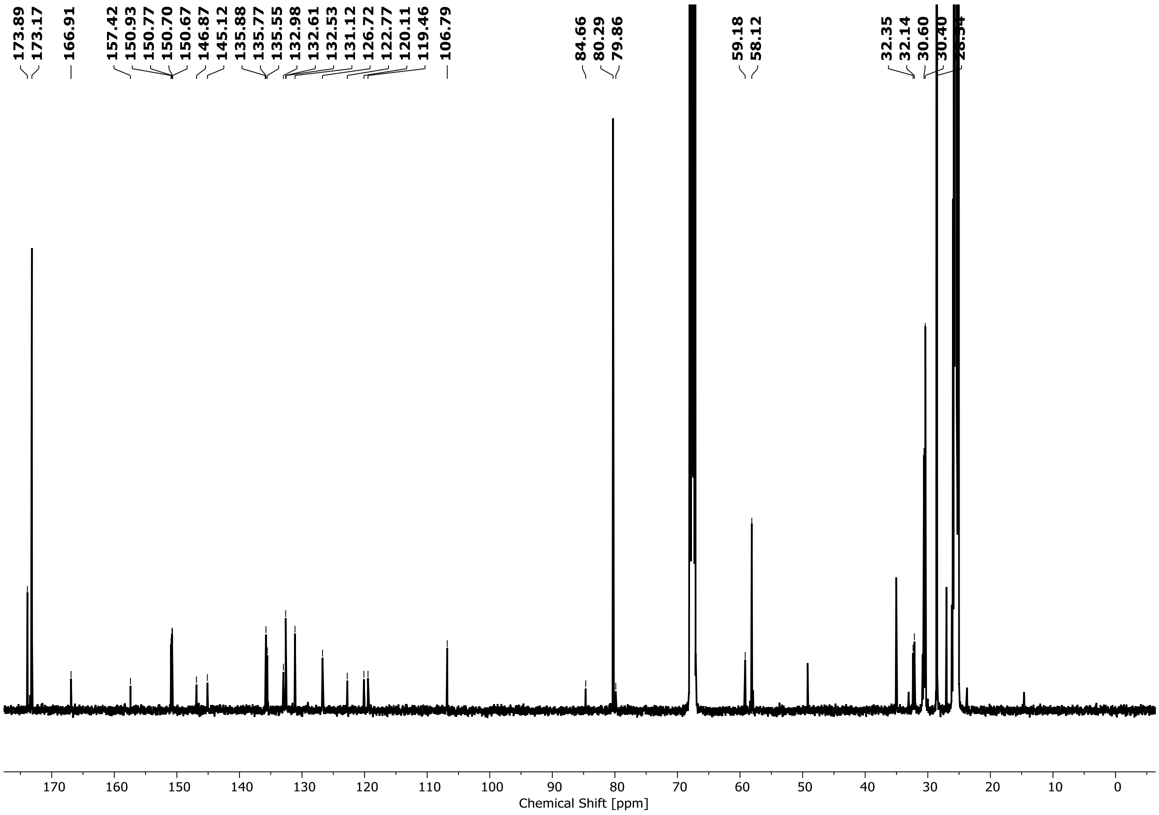

Figure S14 ^{13}C NMR (126 MHz, THF-d_8) of compound **7**.

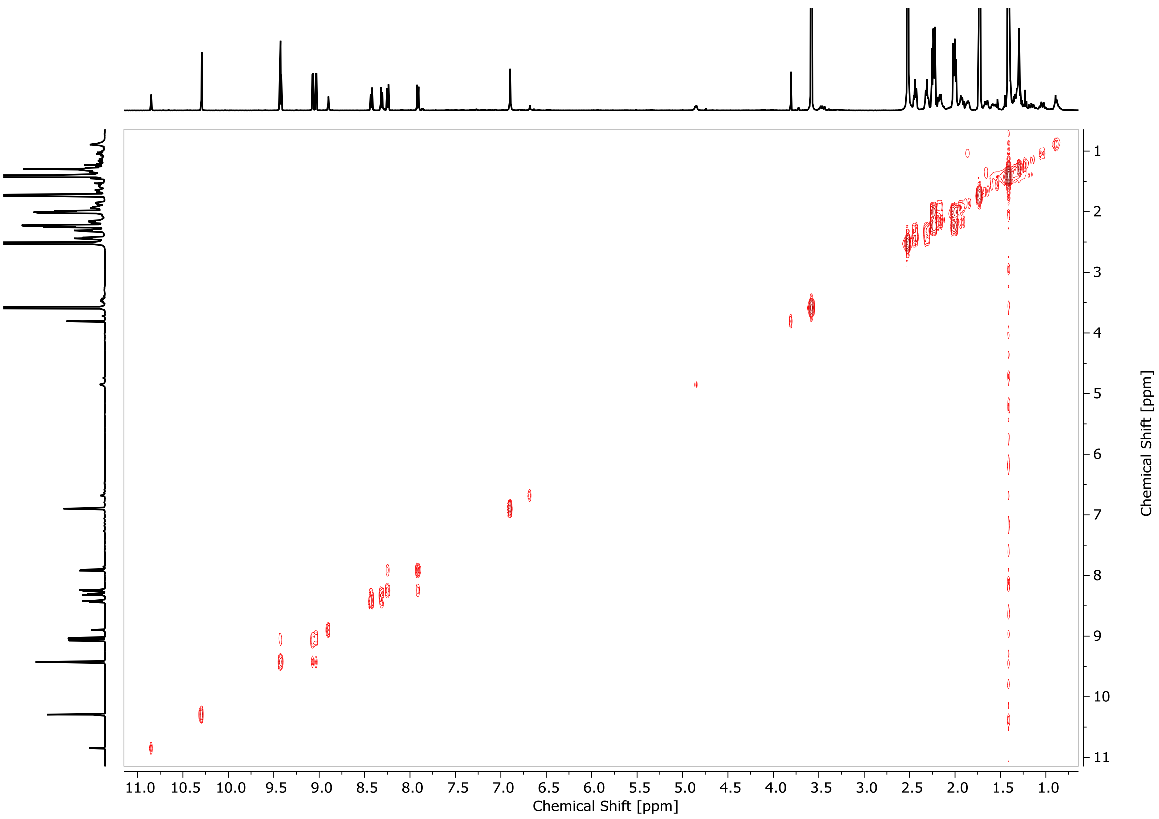

Figure S15 COSY-NMR spectrum of compound **7**.

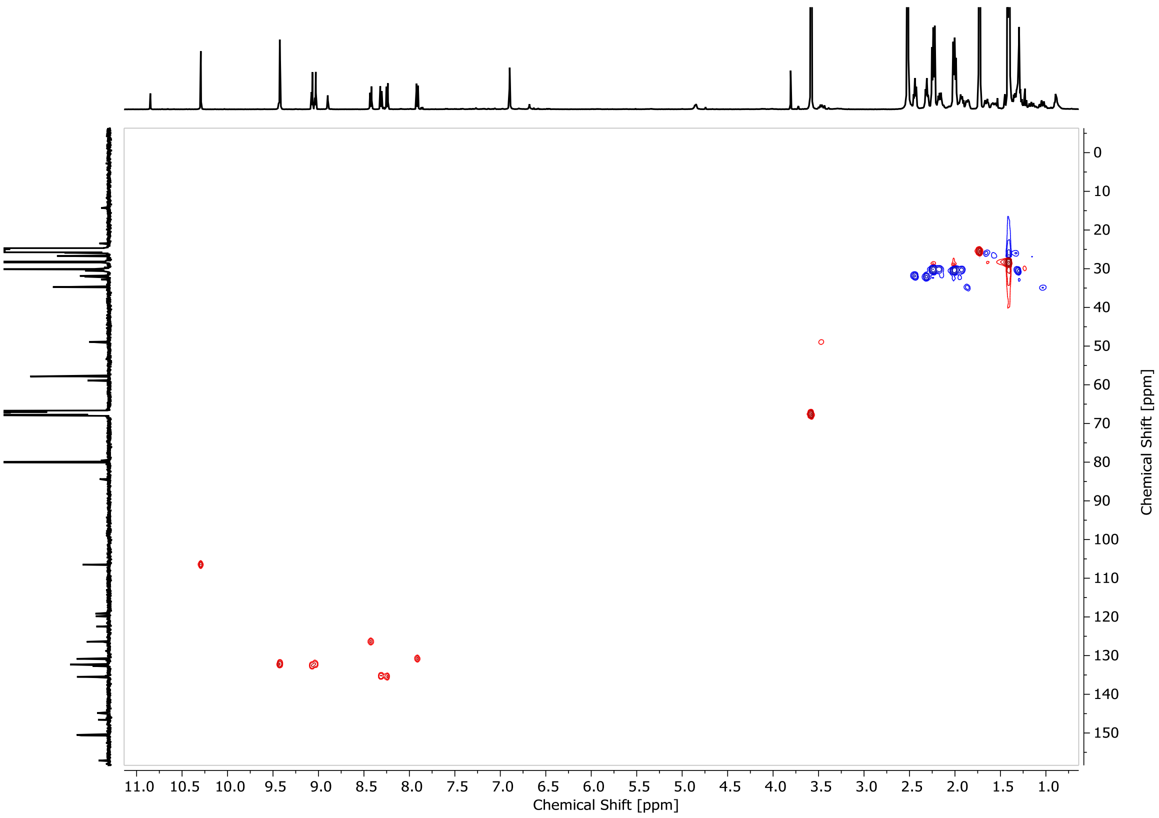

Figure S16 HSQC-NMR spectrum of compound **7**.

t-Bu-G2-mesityl-zinc-porphyrin-t-Bu-phenoxyperylenebisimide Dyad (11)

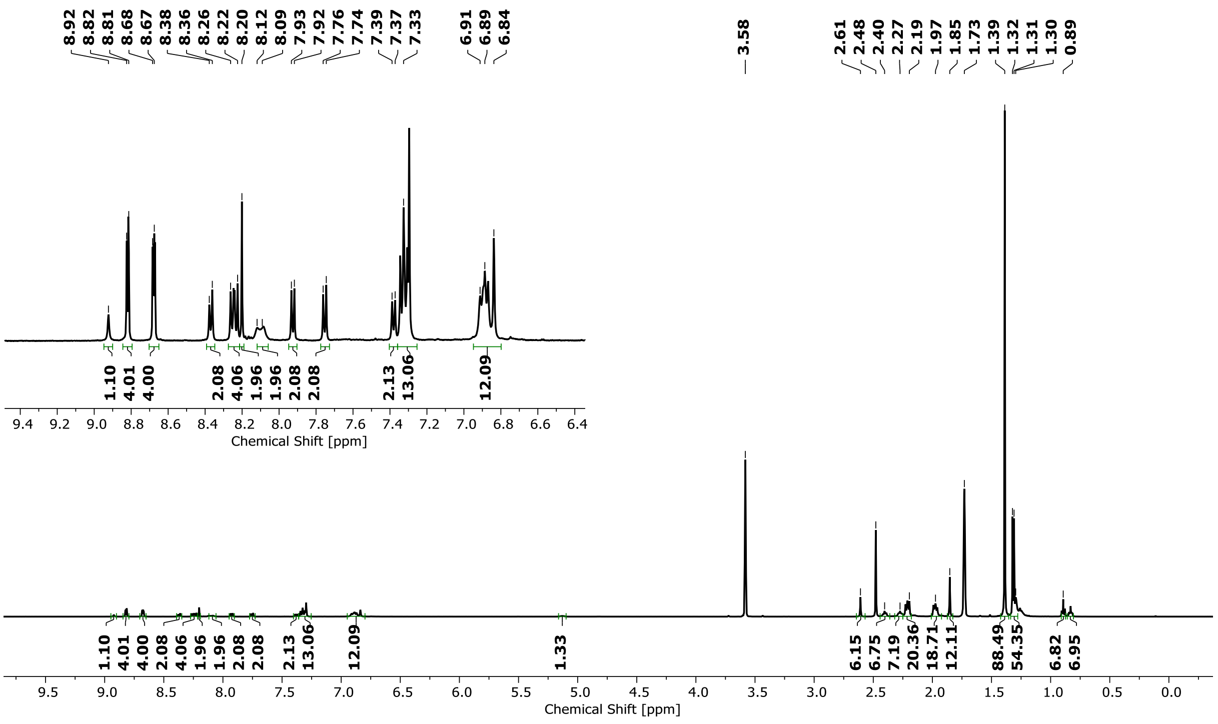

Figure S17 ^{1}H NMR (500 MHz, THF-d_8) of compound **11**.

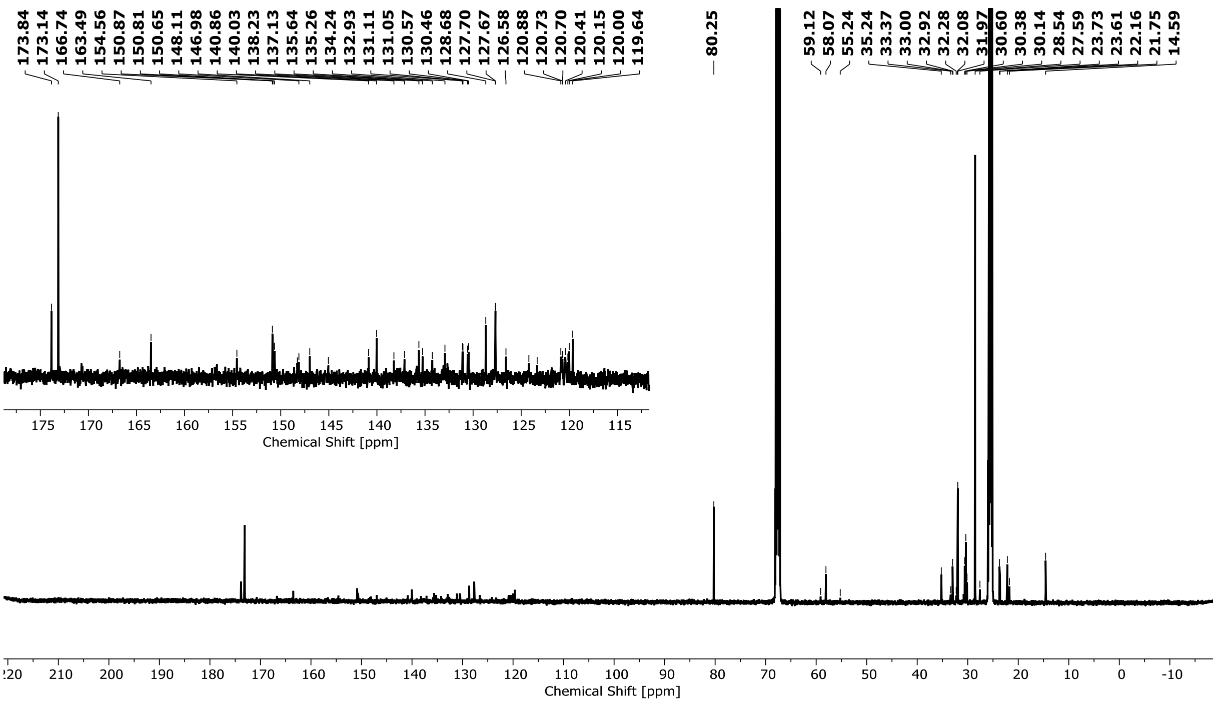

Figure S18 ^{13}C NMR (125 MHz, THF-d_8) of compound **11.**

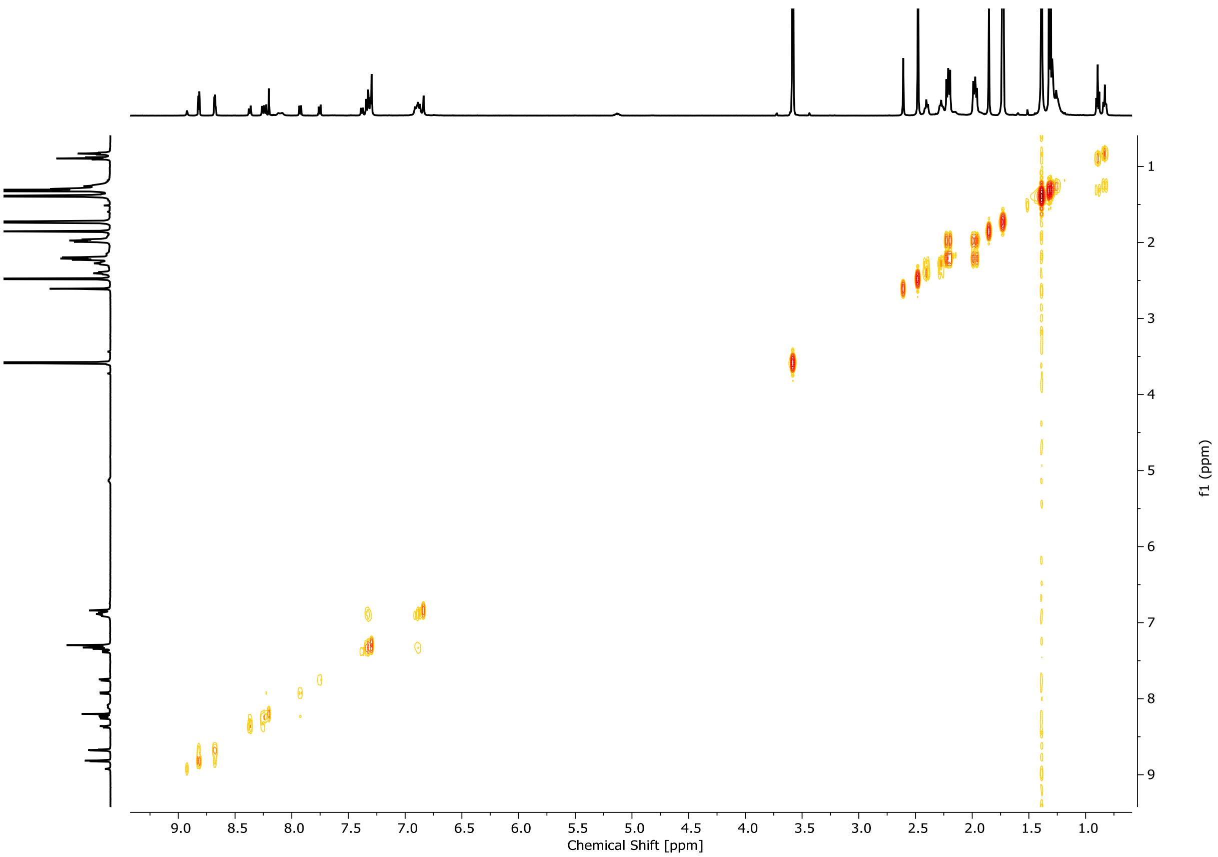

Figure S19 COSY-NMR spectrum of compound **11**.

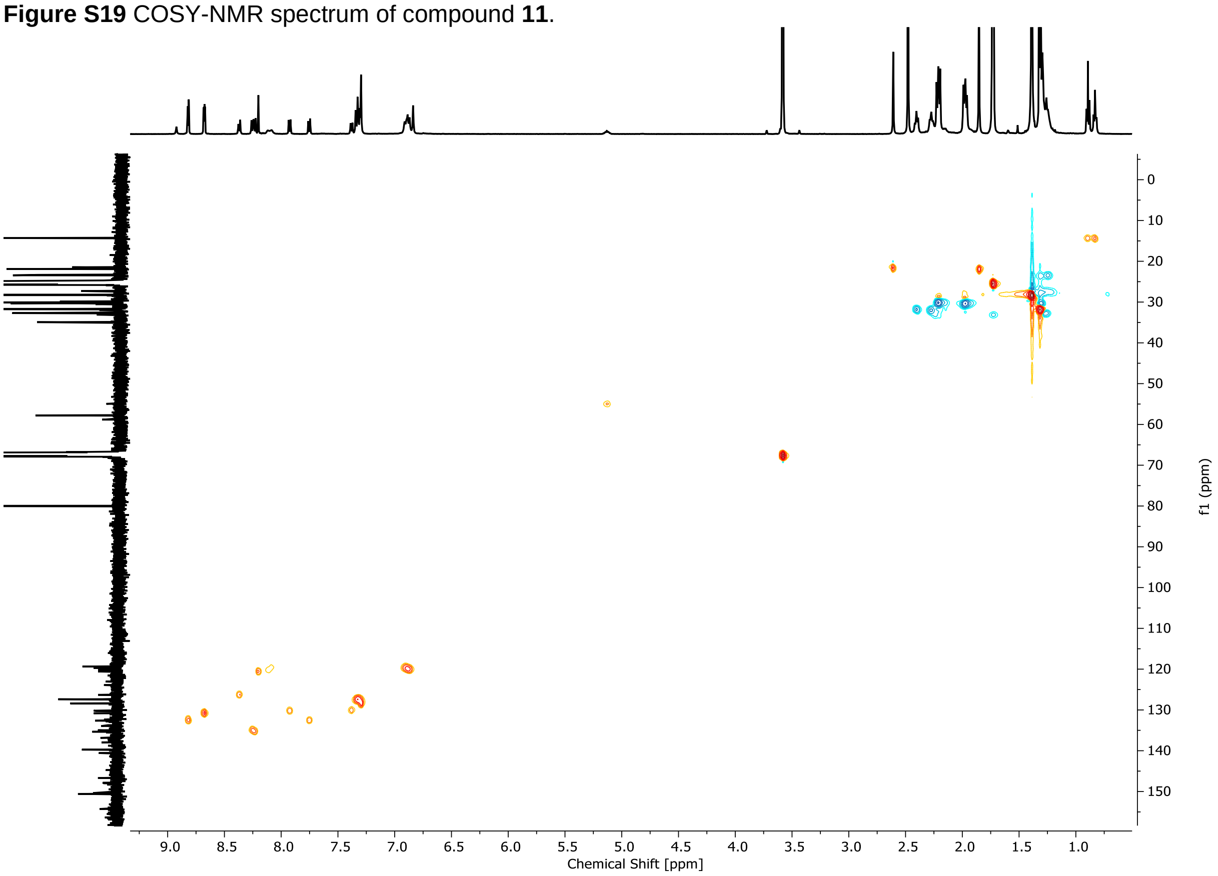

Figure S20 HSQC-NMR spectrum of compound **11**

t-Bu-G2-mesityl-zinc-porphyrin-perylenebisimide Dyad (12)

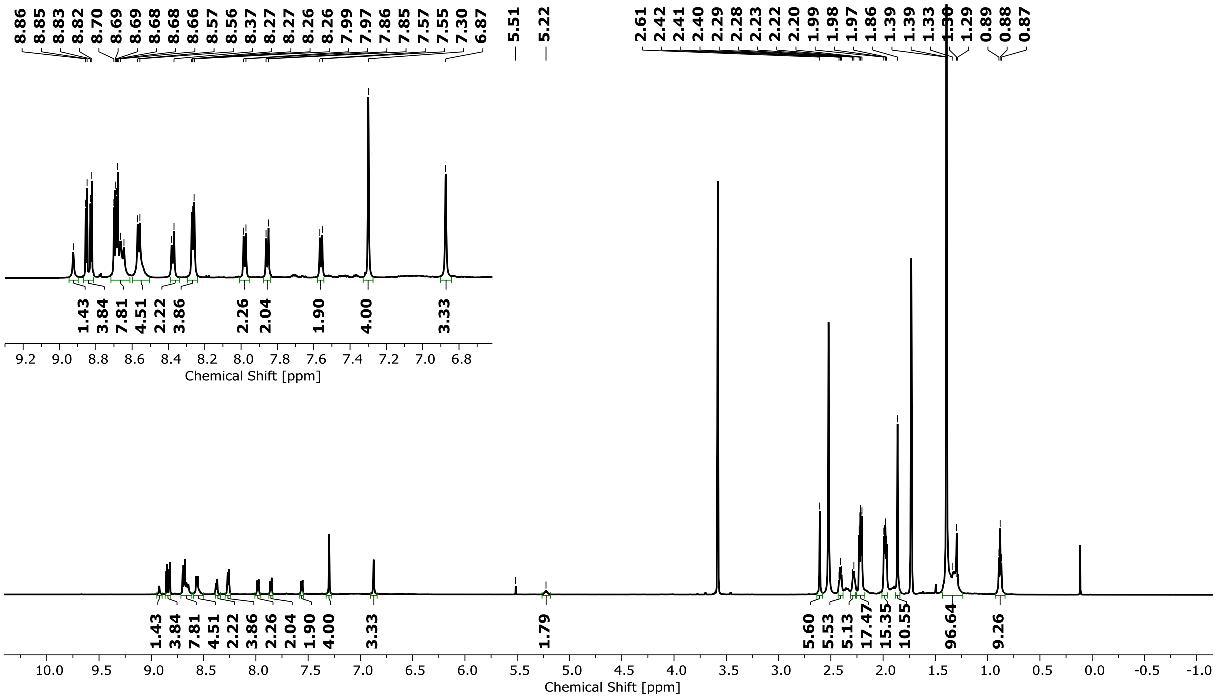

Figure S21 ^{1}H NMR (500 MHz, THF-d_8) of compound **12.**

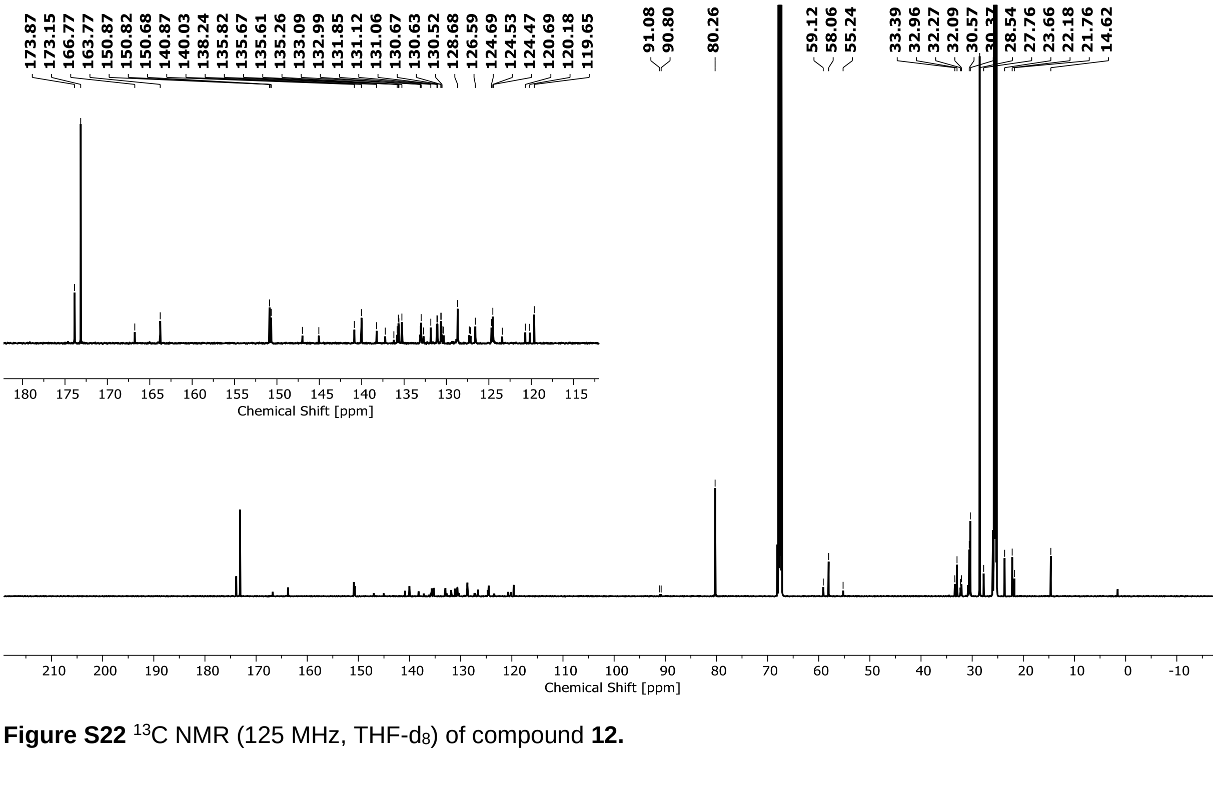

Figure S22 ^{13}C NMR (125 MHz, THF-d_8) of compound **12.**

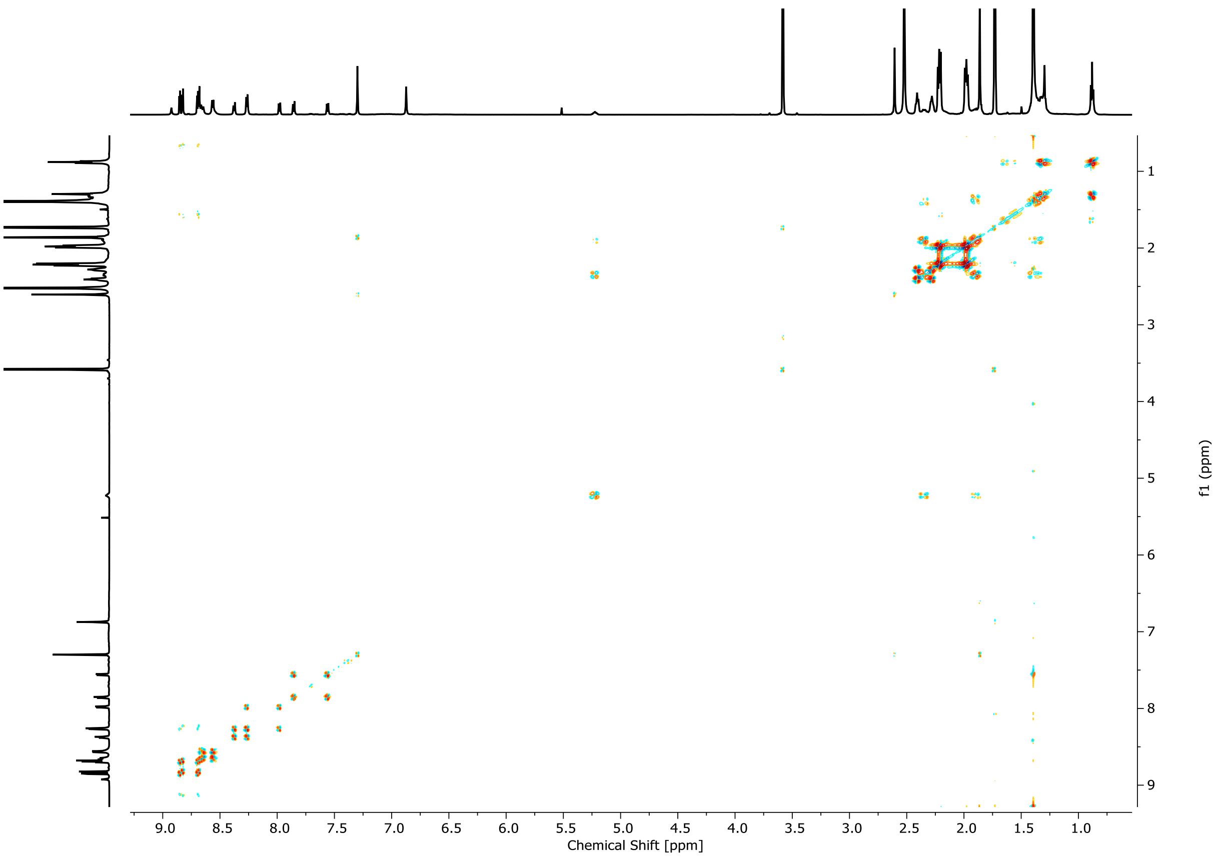

Figure S23 COSY-NMR spectrum of compound **12**.

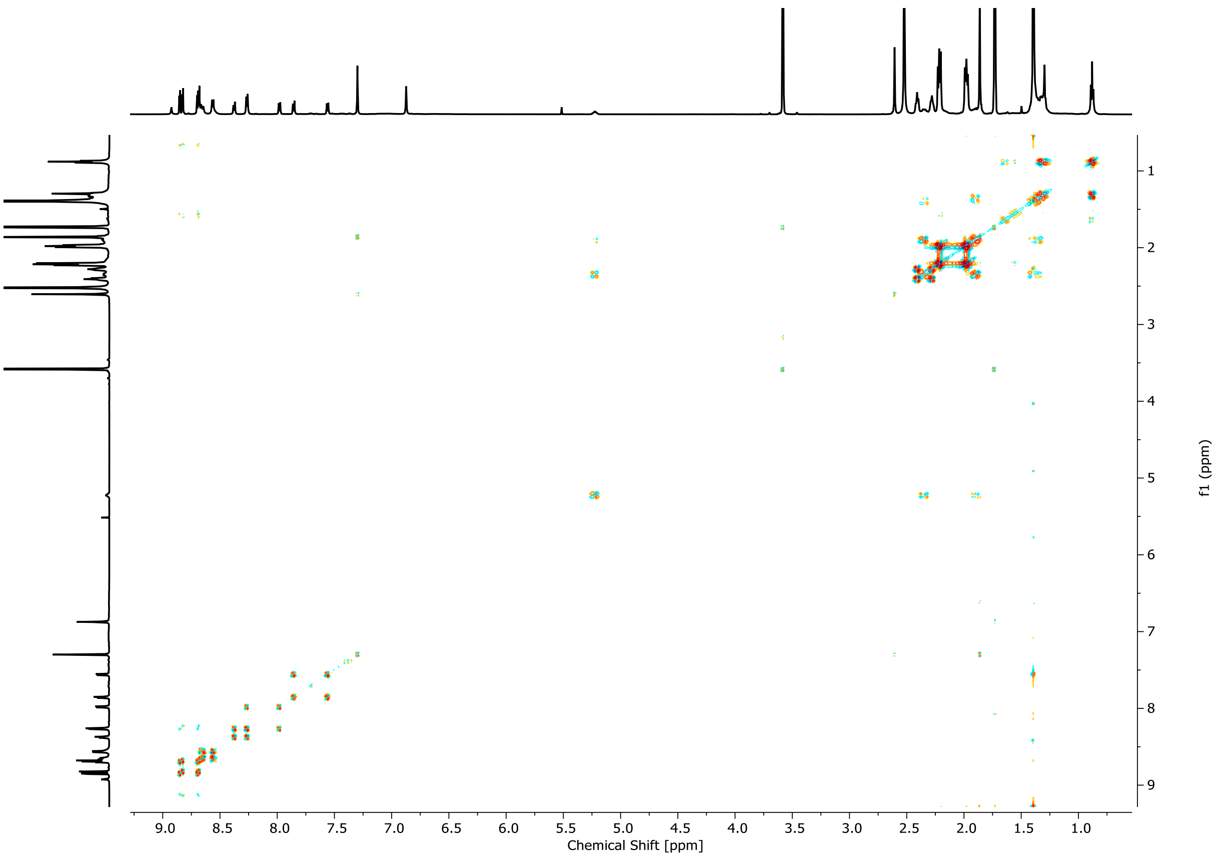

Figure S24 HSQC-NMR spectrum of compound **12**

t-Bu-G2-zinc-porphyrin-perylenebisimide Dyad (13)

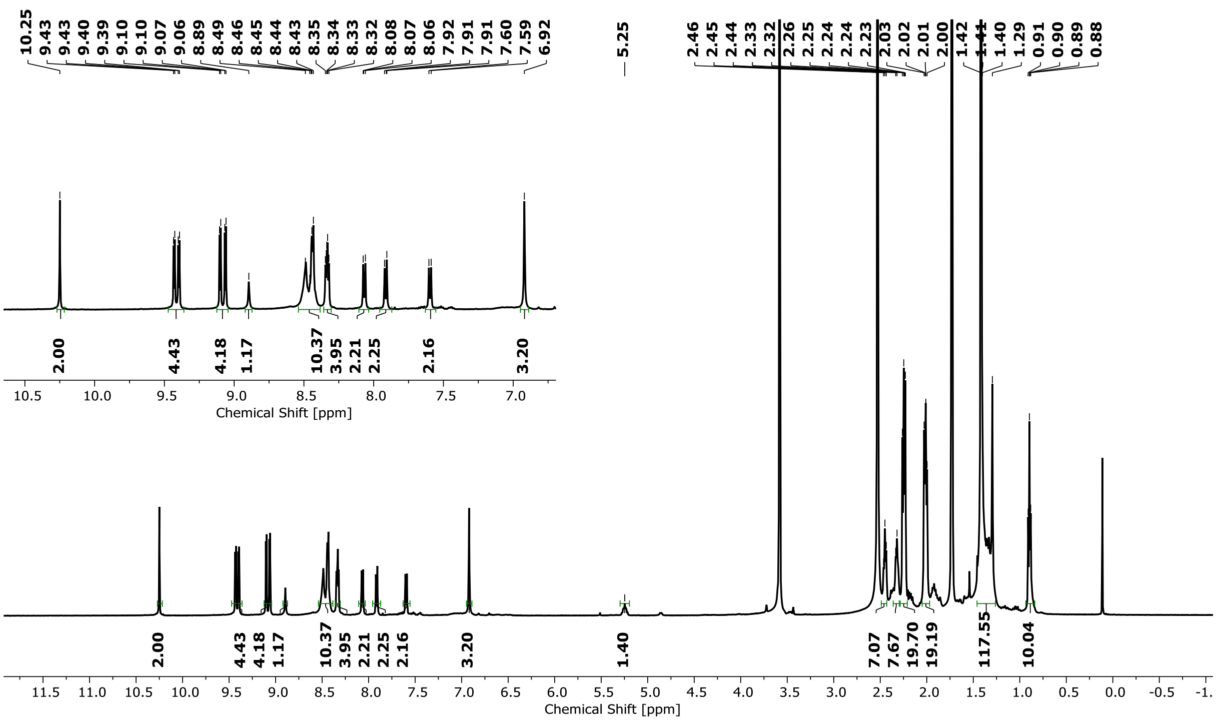

Figure S25 ^{1}H NMR (500 MHz, THF-d_8) of compound **13**.

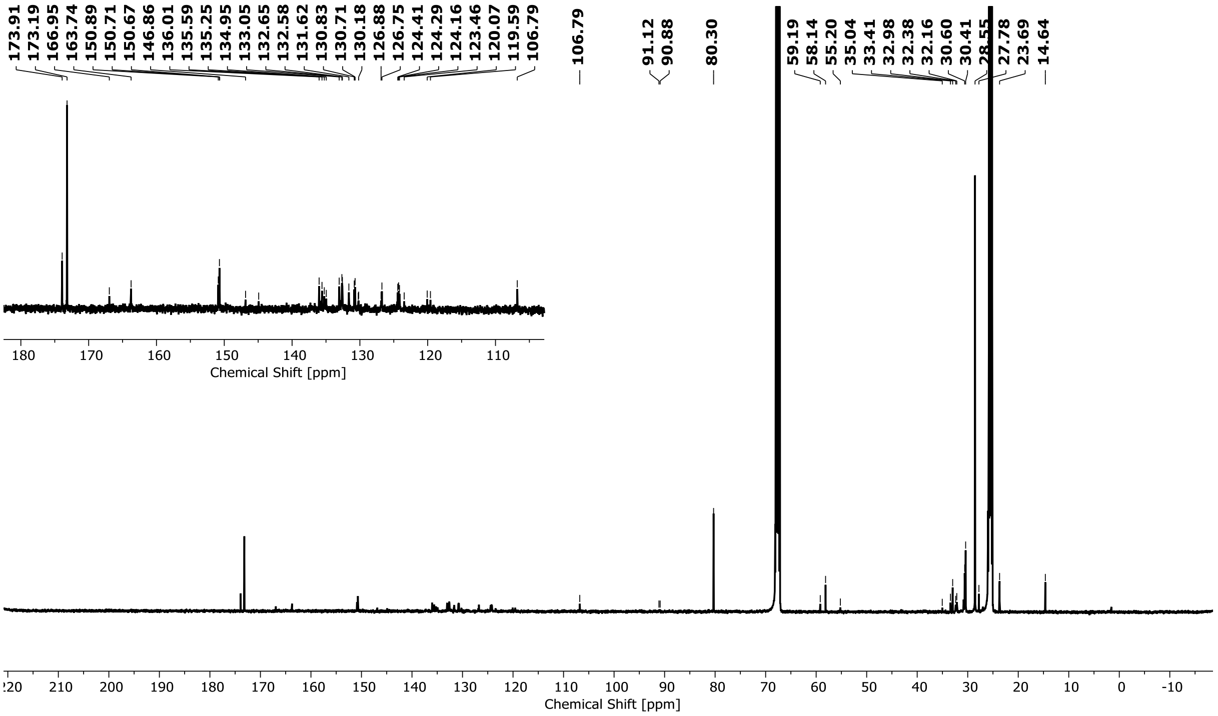

Figure S26 ^{13}C NMR (125 MHz, THF-d_8) of compound **13**.

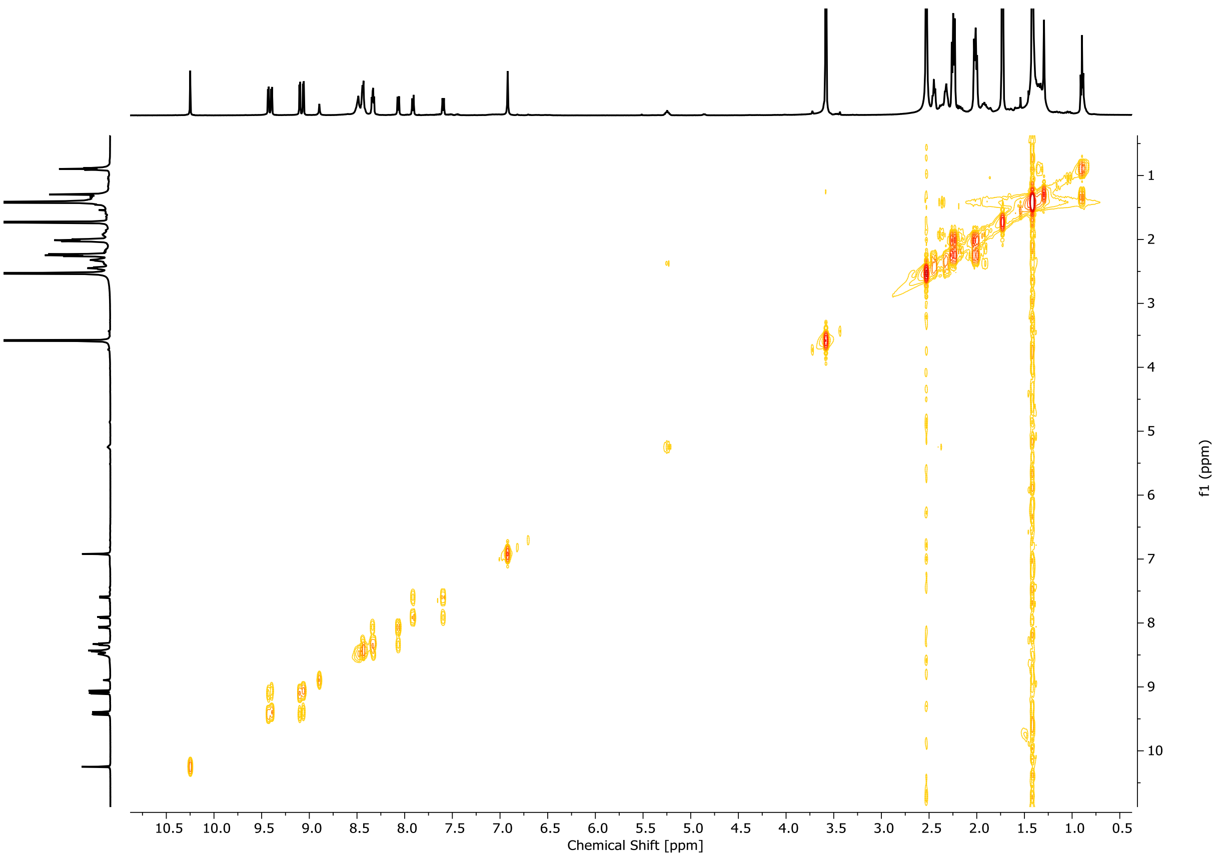

Figure S27 COSY-NMR spectrum of compound **13**.

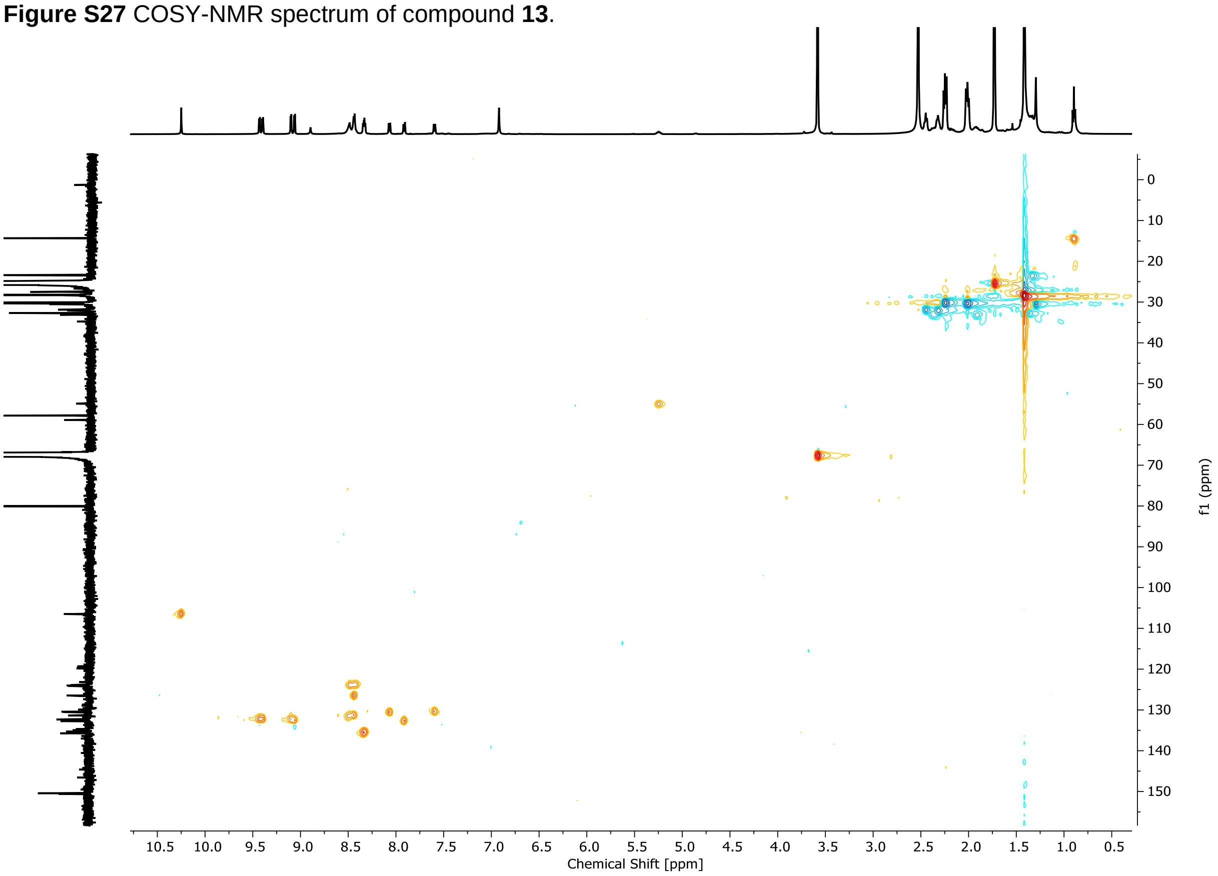

Figure S28 HSQC-NMR spectrum of compound **13**

Carboxylic acid-G2-mesityl-zinc-porphyrin-t-Bu-phenoxyperylenebisimide Dyad (14)

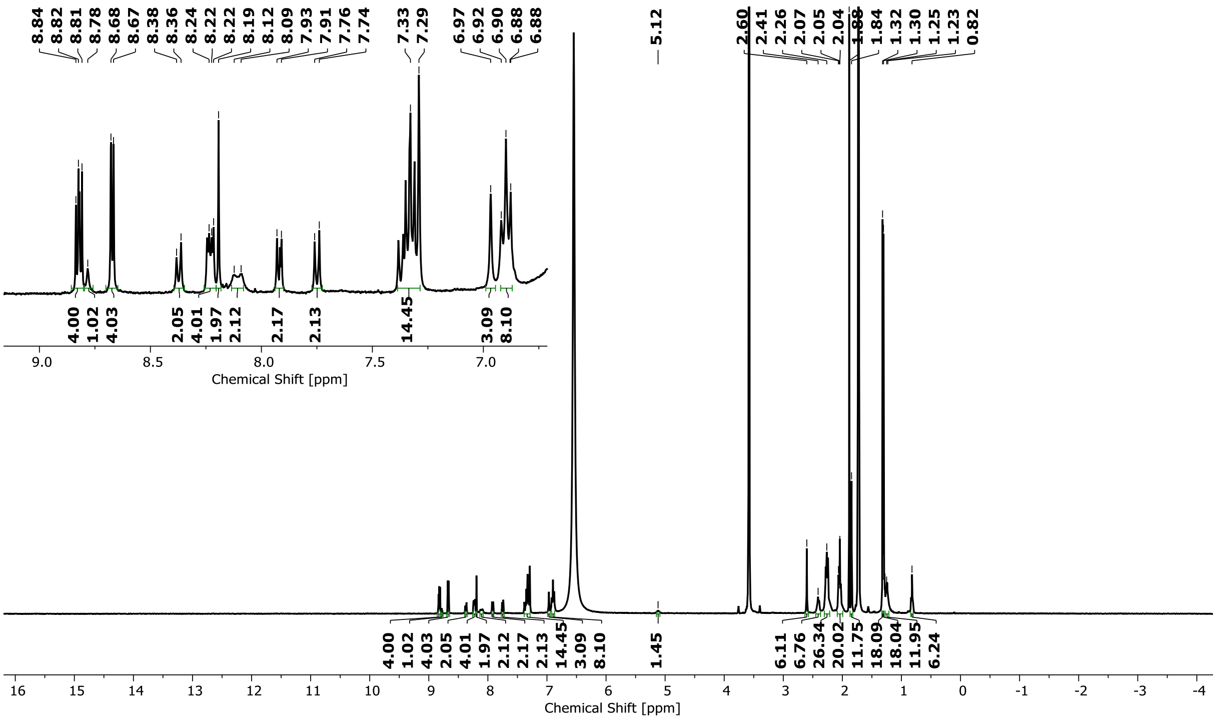

Figure S29 ^{1}H NMR (400 MHz, THF-d8 / 1 vol% TFA-d) of compound **14**.

Carboxylic acid-G2-mesityl-zinc-porphyrin-perylenebisimide Dyad (15)

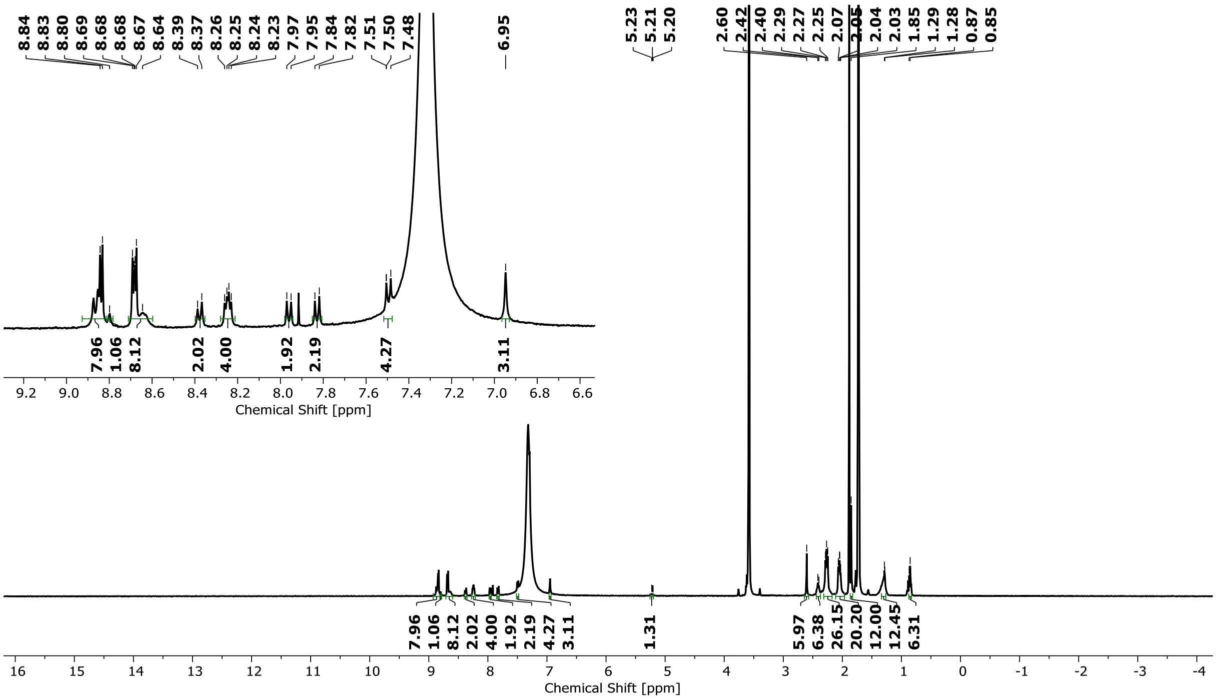

Figure S30 ^{1}H NMR (400 MHz, THF-d8 / 1 vol% TFA-d) of compound **15**.

Carboxylic acid-G2-zinc-porphyrin-perylenebisimide Dyad (16)

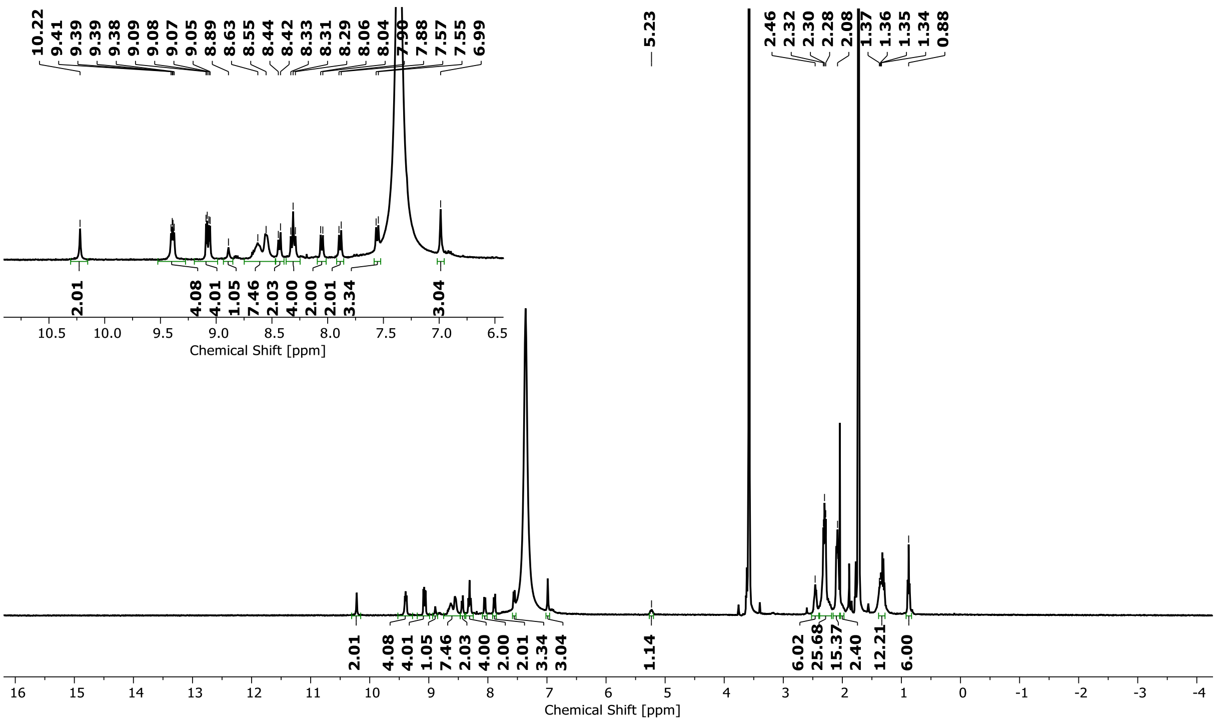

Figure S31 ^{1}H NMR (400 MHz, THF-d_8 / 1 vol% TFA-d) of compound **16**.

4. Mass Spectra

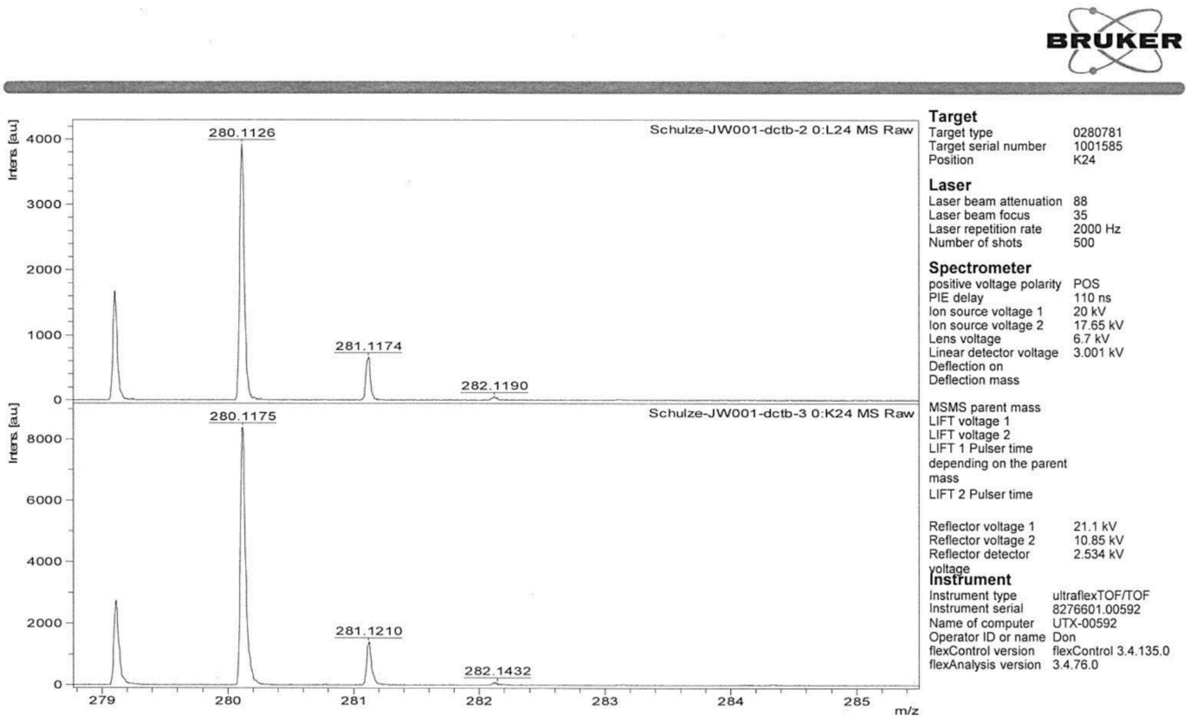

Figure S32 MS spectrum (MALDI-TOF) of compound **1**

.

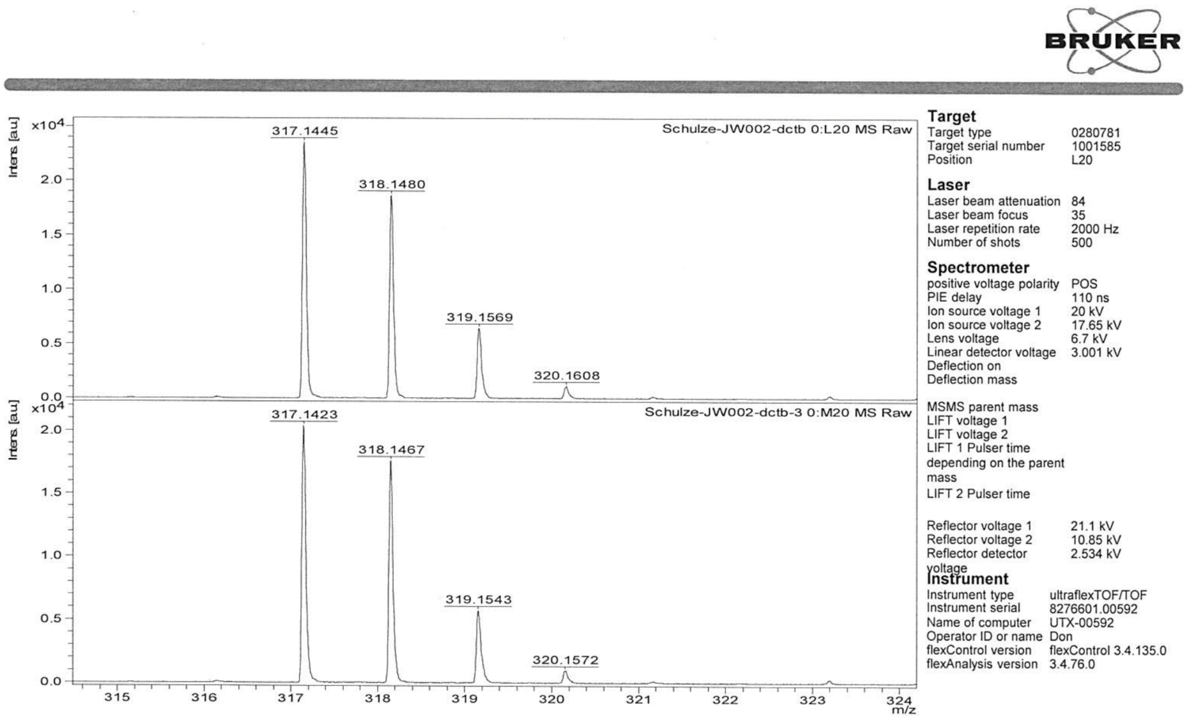

Figure S33 MS spectrum (MALDI-TOF) of compound **2**.

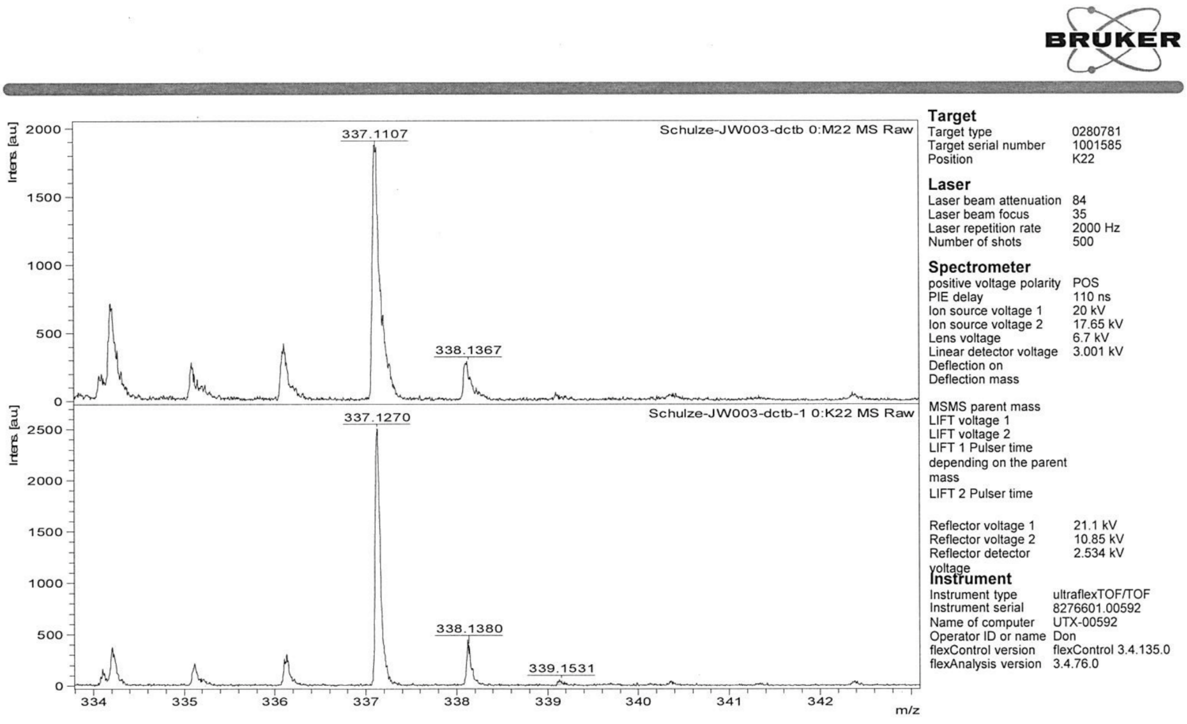

Figure S34 MS spectrum (MALDI-TOF) of compound **3**.

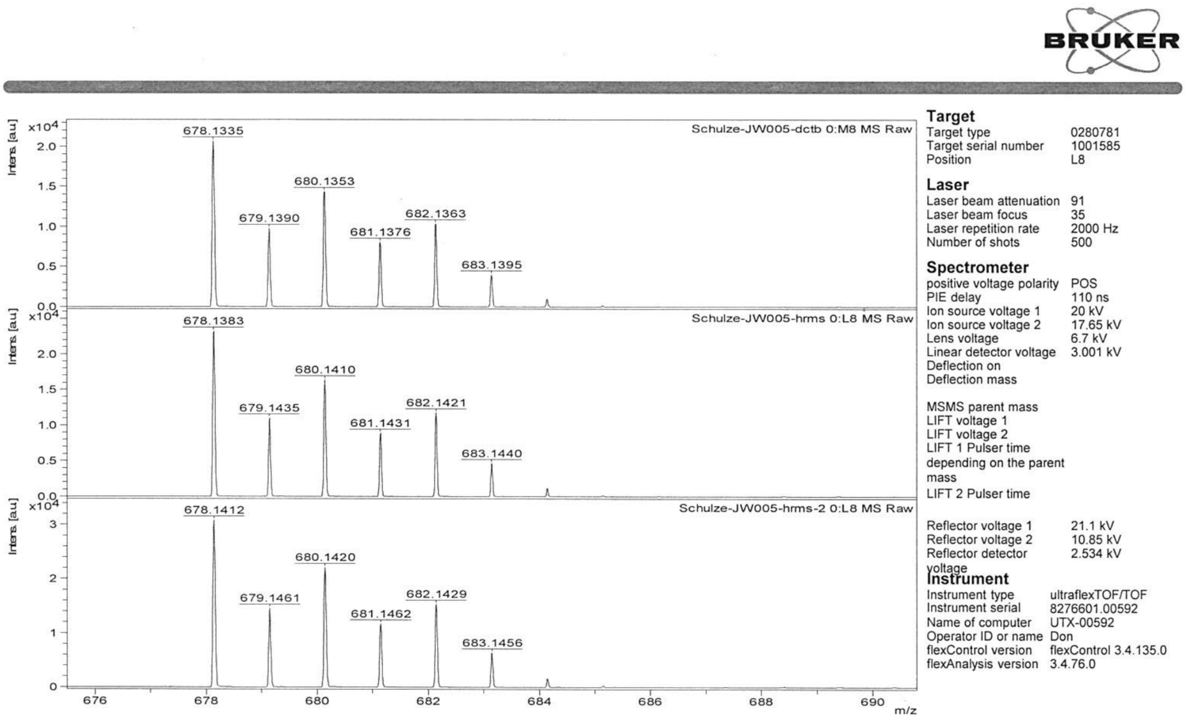

Figure S35 HRMS spectrum (MALDI-TOF) of compound **4**.

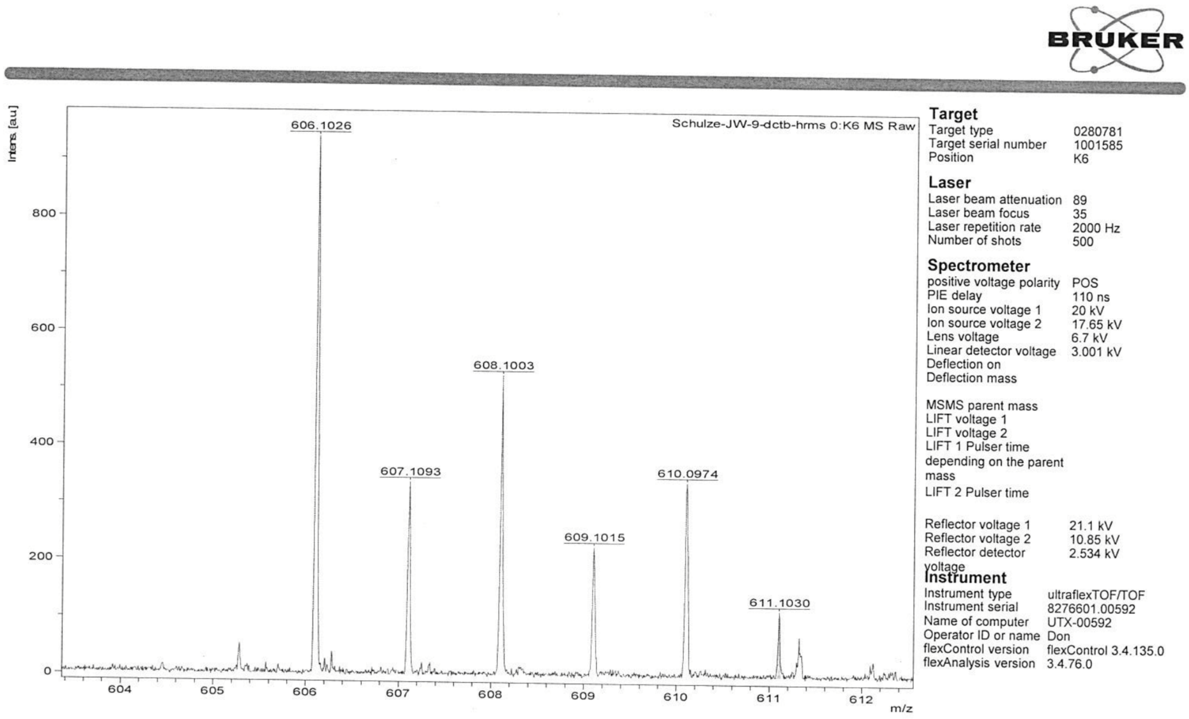

Figure S36 HRMS spectrum (MALDI-TOF) of compound **5**.

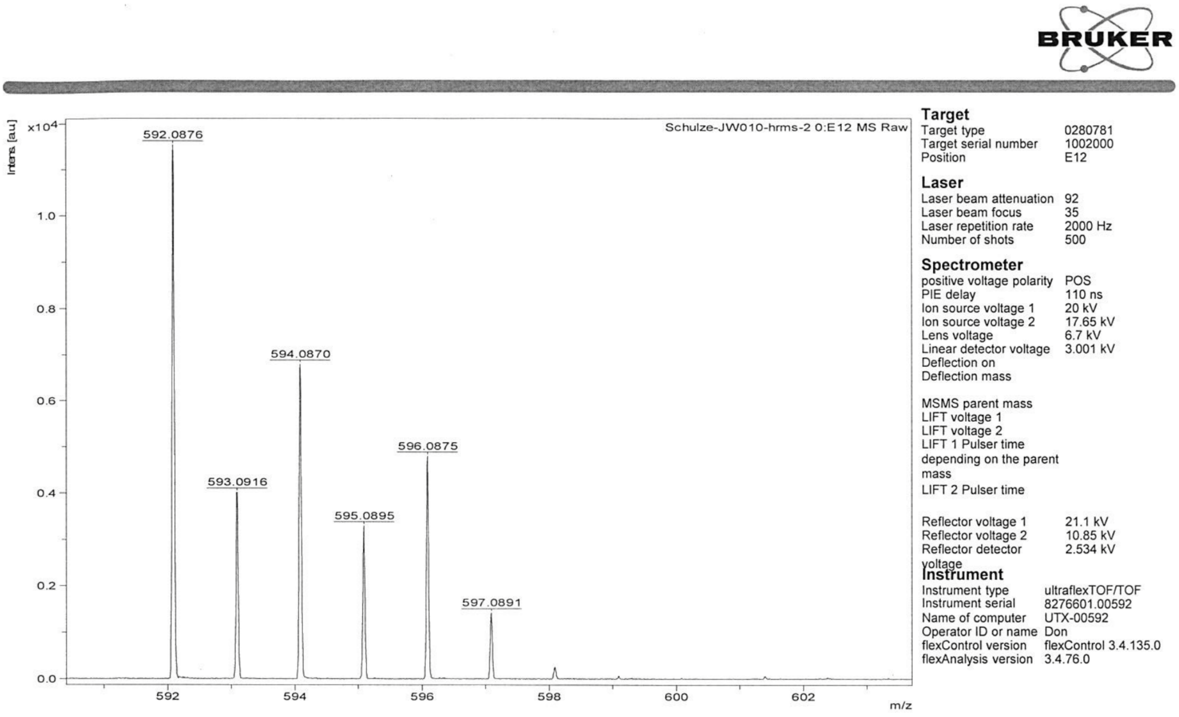

Figure S37 HRMS spectrum (MALDI-TOF) of compound **6**.

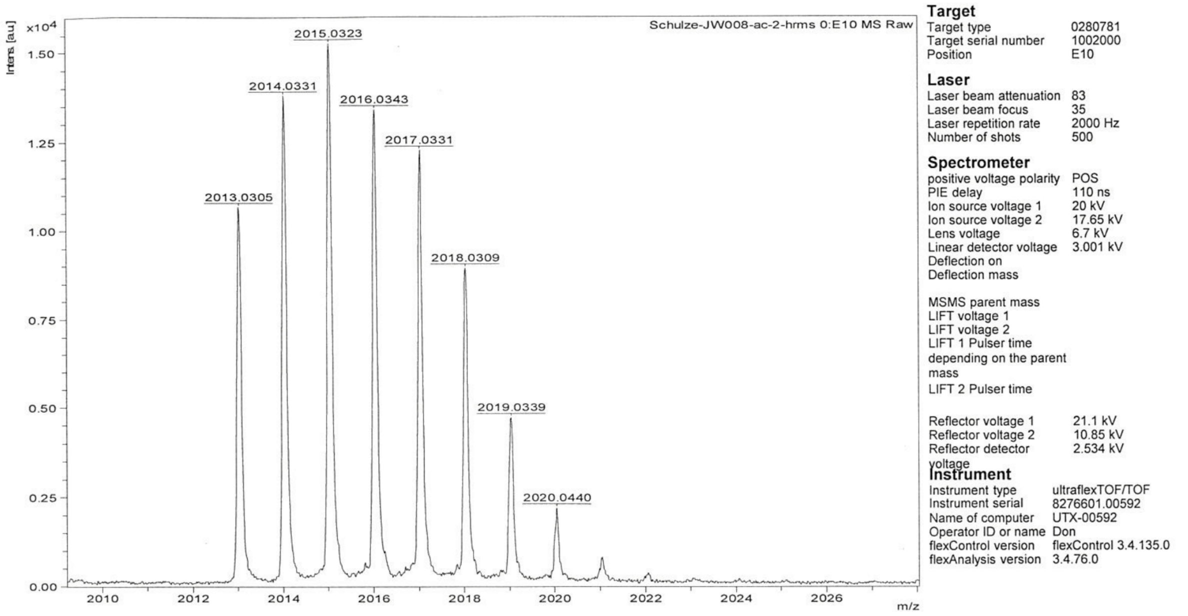

Figure S38 HRMS spectrum (MALDI-TOF) of compound **7**.

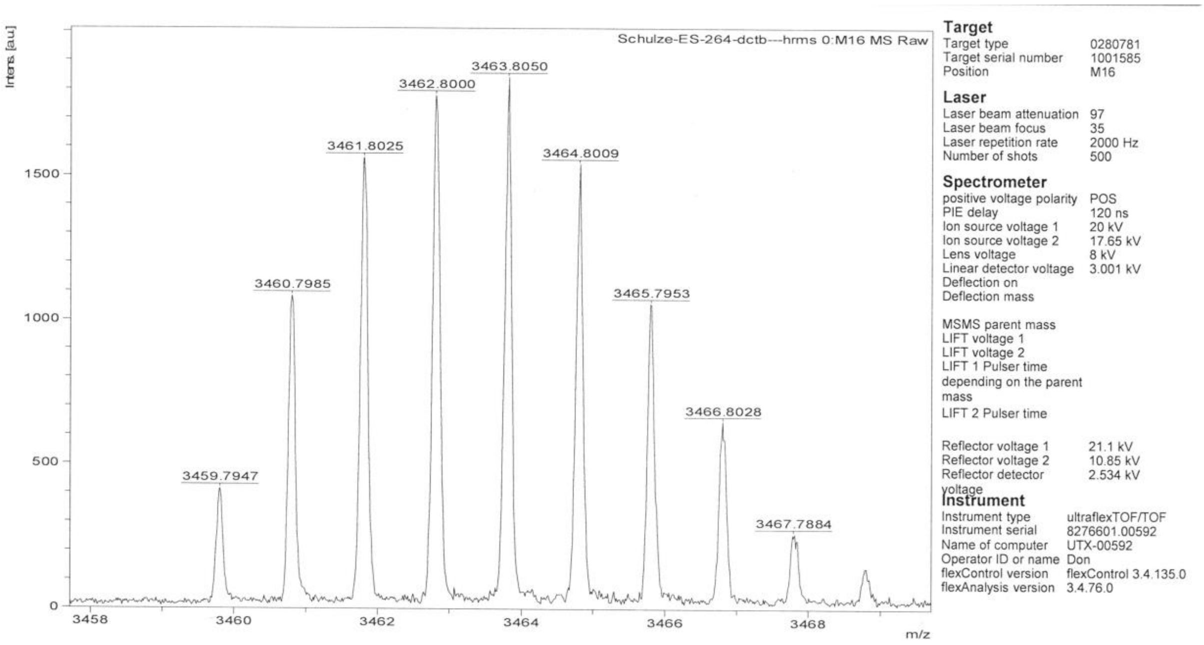

Figure S39 HRMS spectrum (MALDI-TOF) of compound **11.**

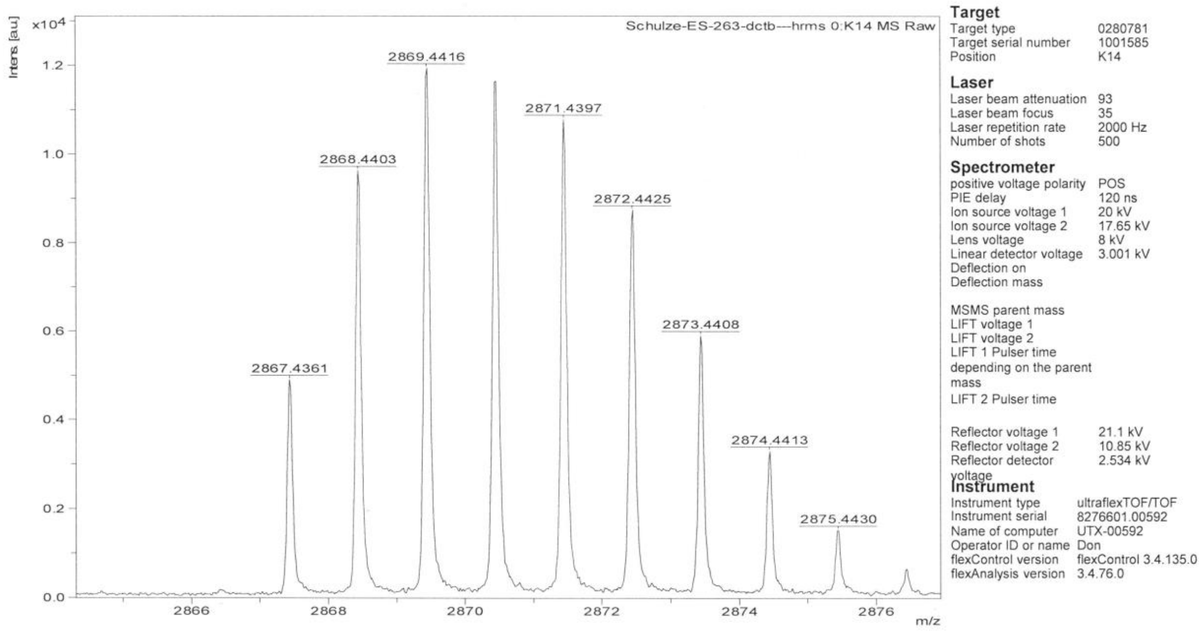

Figure S40 HRMS spectrum (MALDI-TOF) of compound **12.**

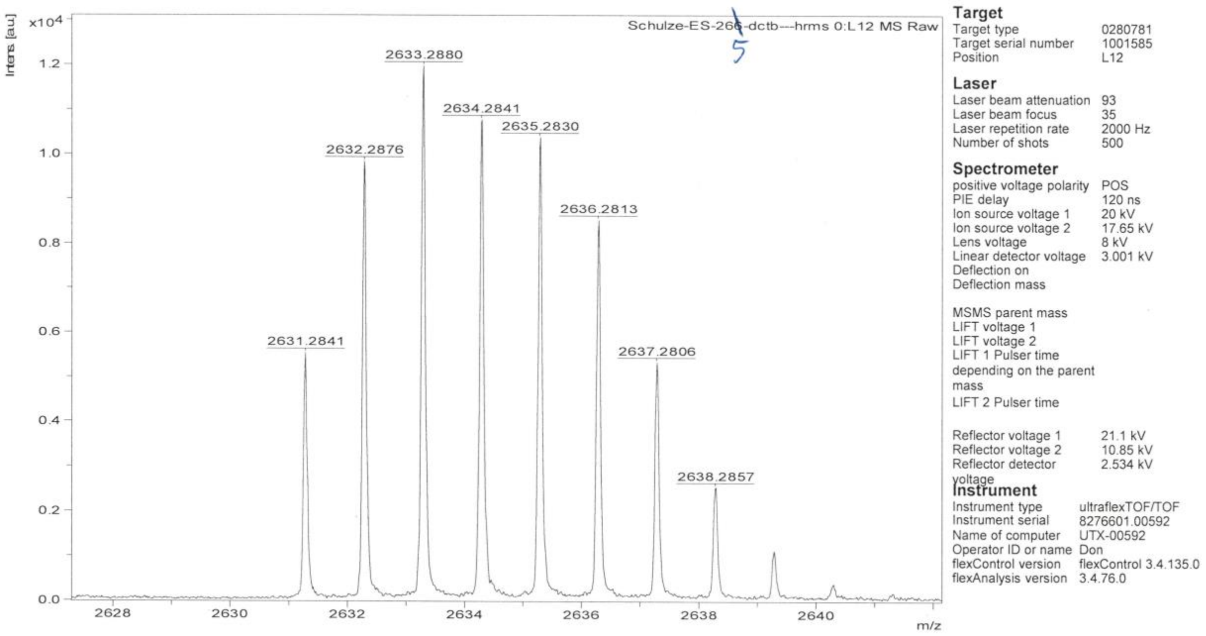

Figure S41 HRMS spectrum (MALDI-TOF) of compound **13.**

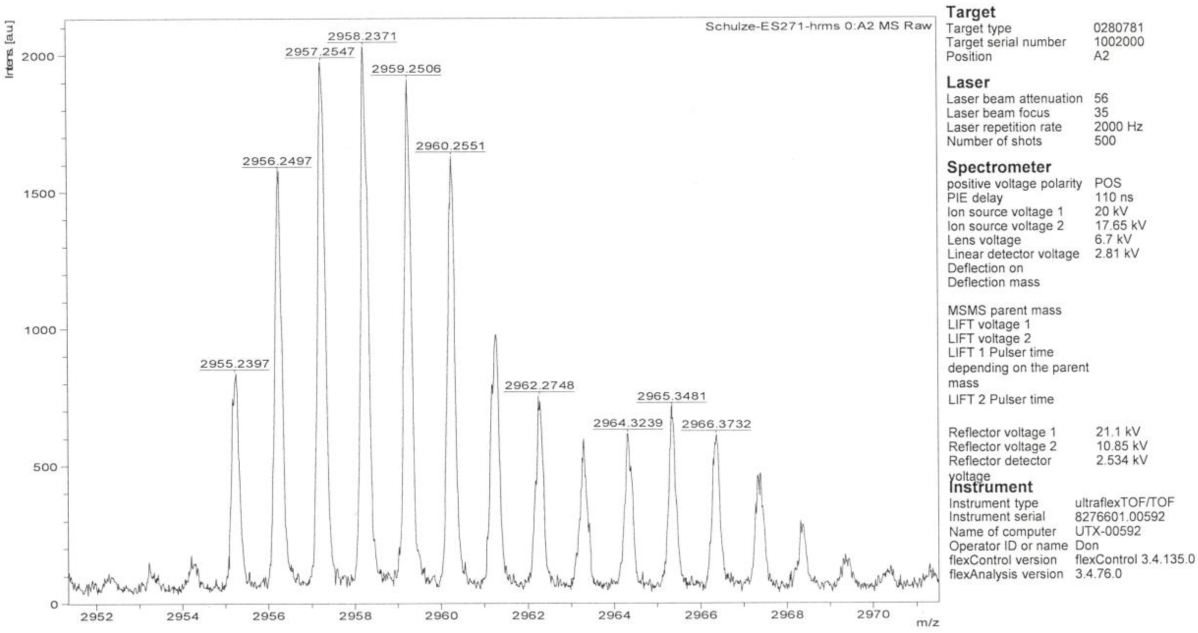

Figure S42 HRMS spectrum (MALDI-TOF) of compound **14.**

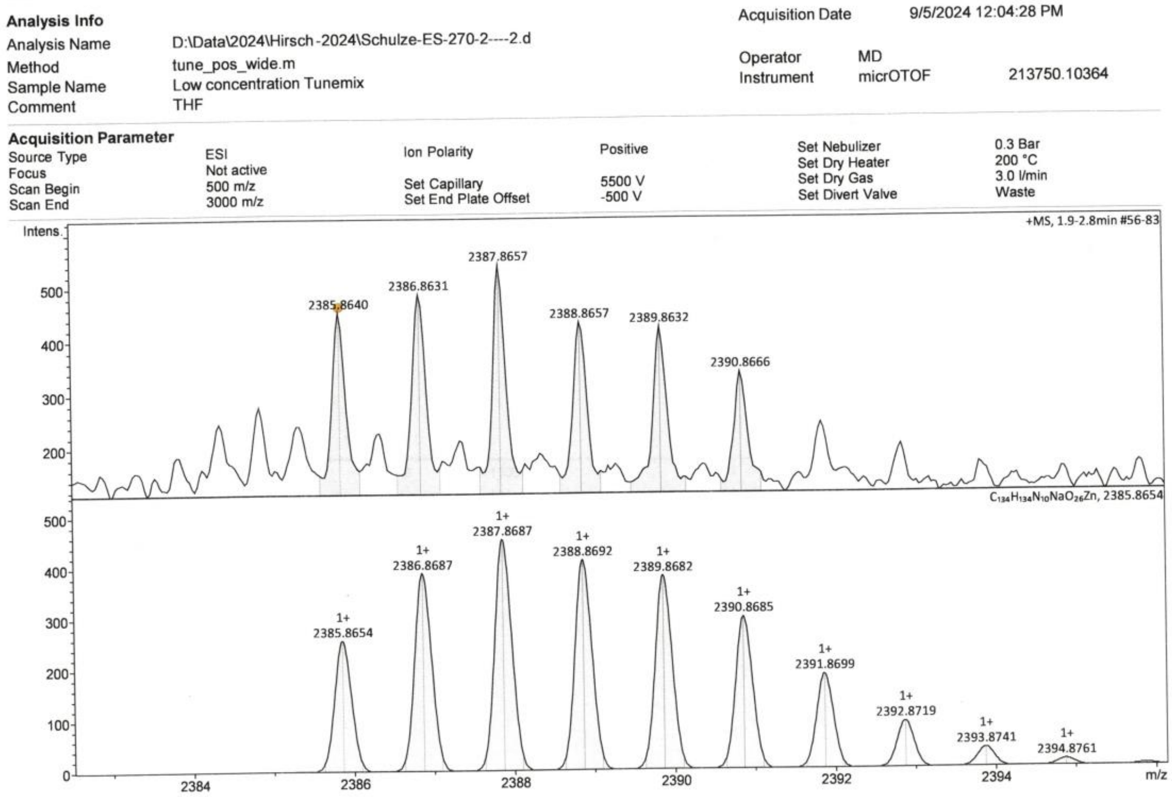

Figure S43 HRMS spectrum (ESI-TOF – positive mode) of compound **15** (Top. Simulated measured spectrum; Bottom: Simulated spectrum).

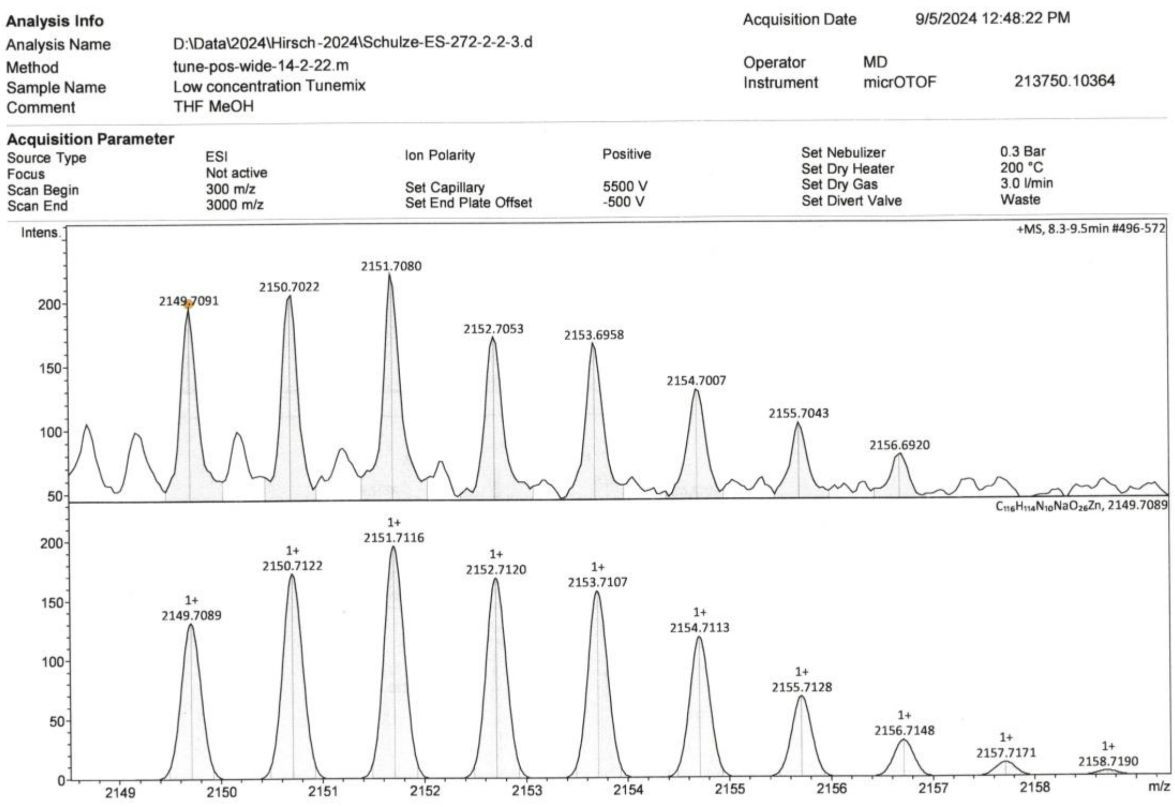

Figure S44 HRMS spectrum (ESI-TOF – positive mode) of compound **16** (Top. Simulated measured spectrum; Bottom: Simulated spectrum).

5. UV/Vis and Fluorescence Spectra

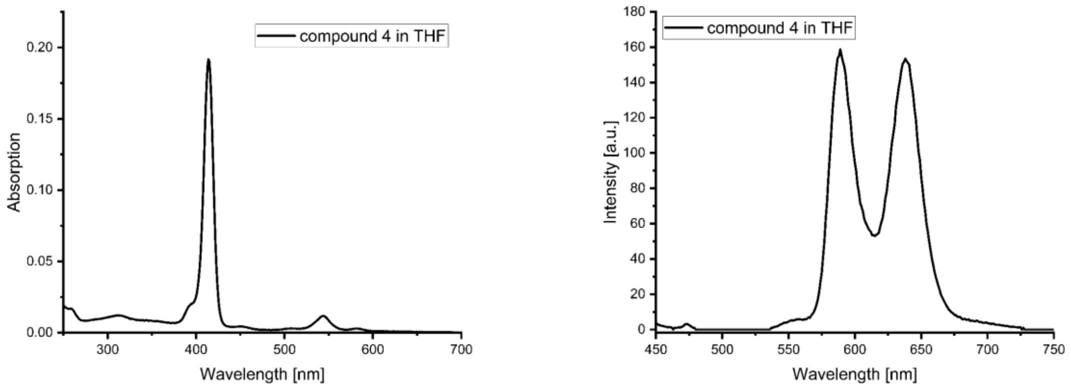

Figure S45 Left: UV/Vis spectrum of compound **4** in THF; Right: Fluorescence spectrum of compound **4** in THF, excited at 415 nm

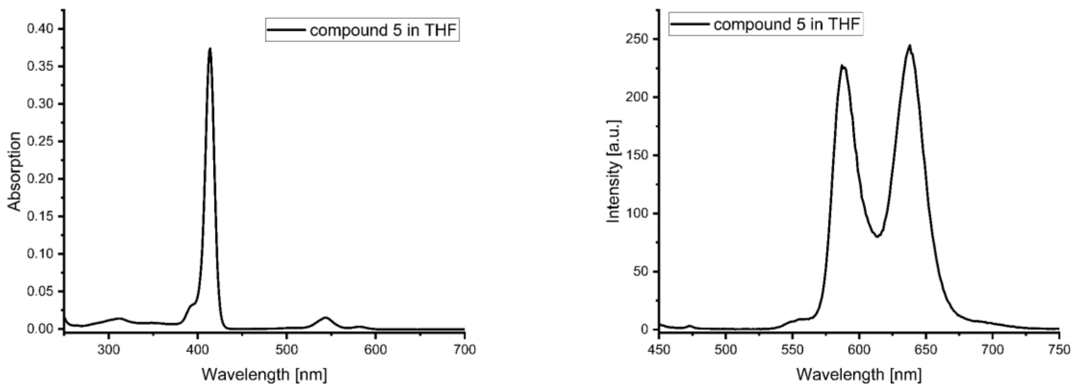

Figure S46 Left: UV/Vis spectrum of compound **5** in THF; Right: Fluorescence spectrum of compound **5** in THF, excited at 415 nm.

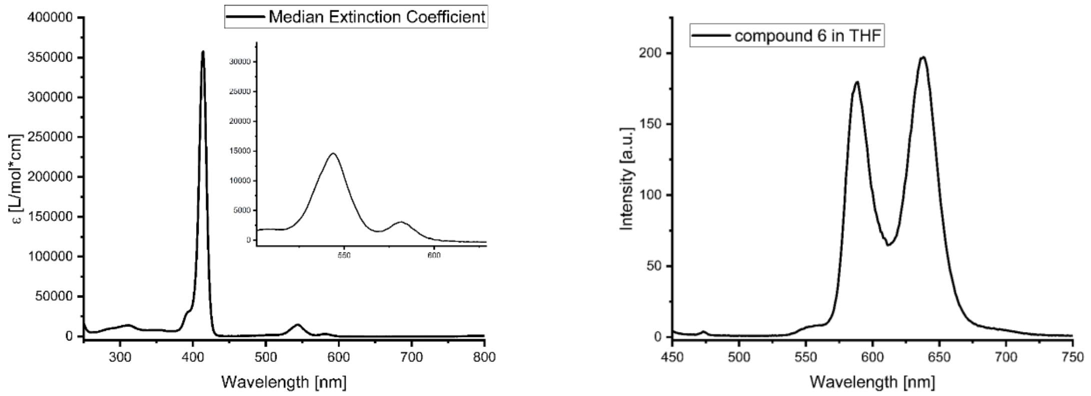

Figure S47 Left: UV/Vis spectrum of compound **6** in THF; Right: Fluorescence spectrum of compound **6** in THF, excited at 415 nm.

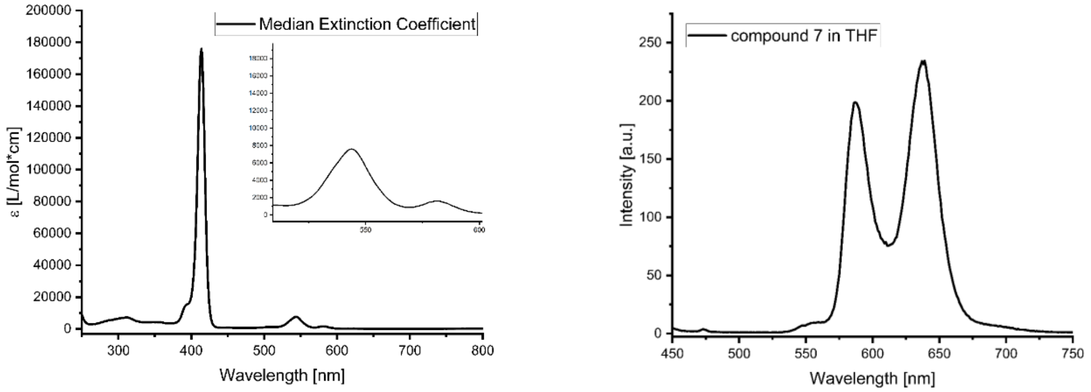

Figure S48 Left: UV/Vis spectrum of compound **7** in THF; Right: Fluorescence spectrum of compound **7** in THF, excited at 415 nm.

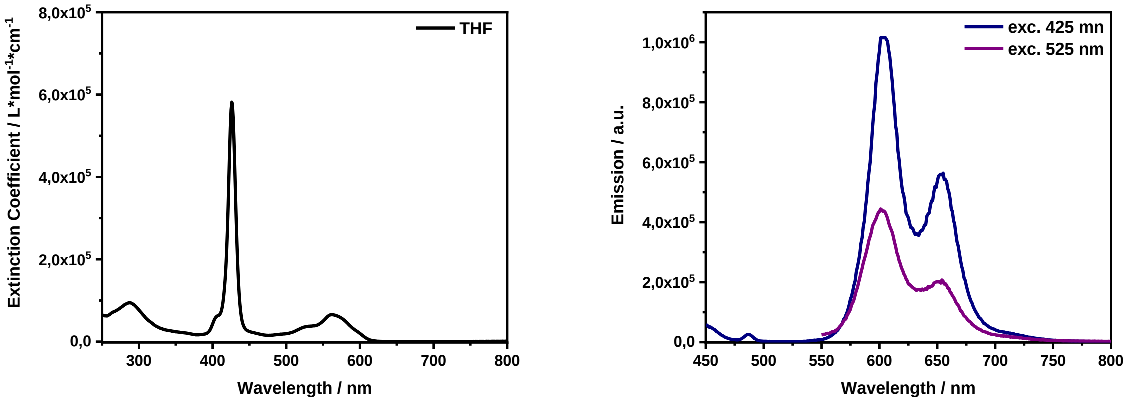

Figure S49 Left: UV/Vis spectrum of compound **11** in THF; Right: Fluorescence spectrum of compound **11** in THF, excited at 425 nm and 525 nm.

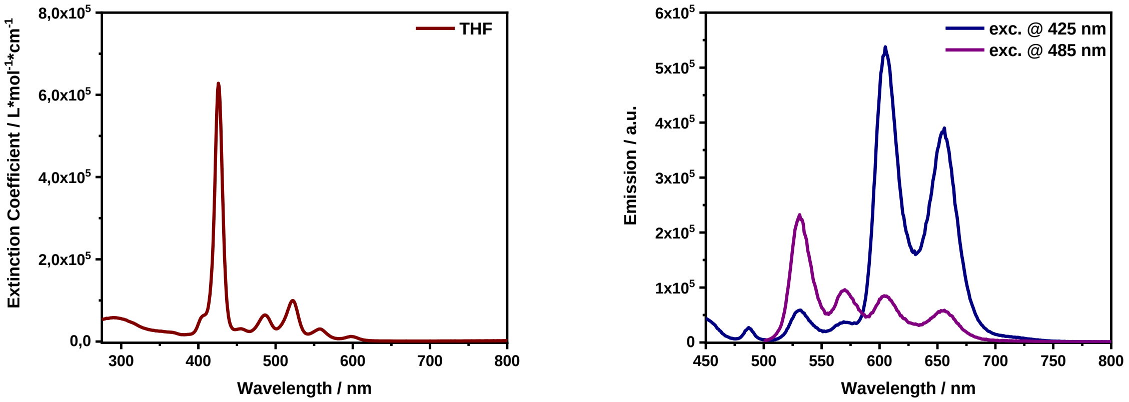

Figure S50 Left: UV/Vis spectrum of compound **12** in THF; Right: Fluorescence spectrum of compound **12** in THF, excited at 425 nm and 485 nm.

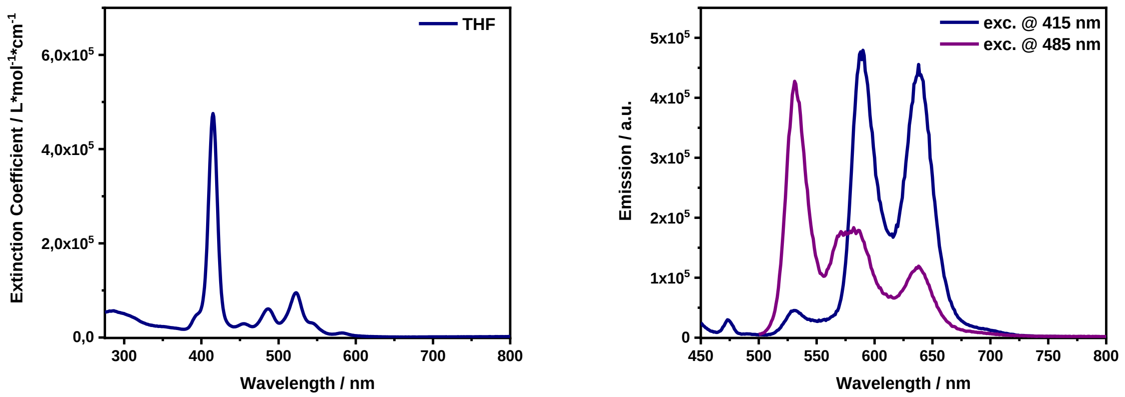

Figure S51 Left: UV/Vis spectrum of compound **13** in THF; Right: Fluorescence spectrum of compound **13** in THF, excited at 415 nm and 485 nm.

Molar extinction coefficients of the amphiphiles **14**, **15**, and **16** were determined from a mixture of THF and 10 mM aqueous NaOH solution (1:1 v/v). These are observed to be lower than their respective *tert*-butyl precursors; this can either be reasoned with just the change in solvent or possibly due to the amphiphiles not being perfectly individualized under these conditions.

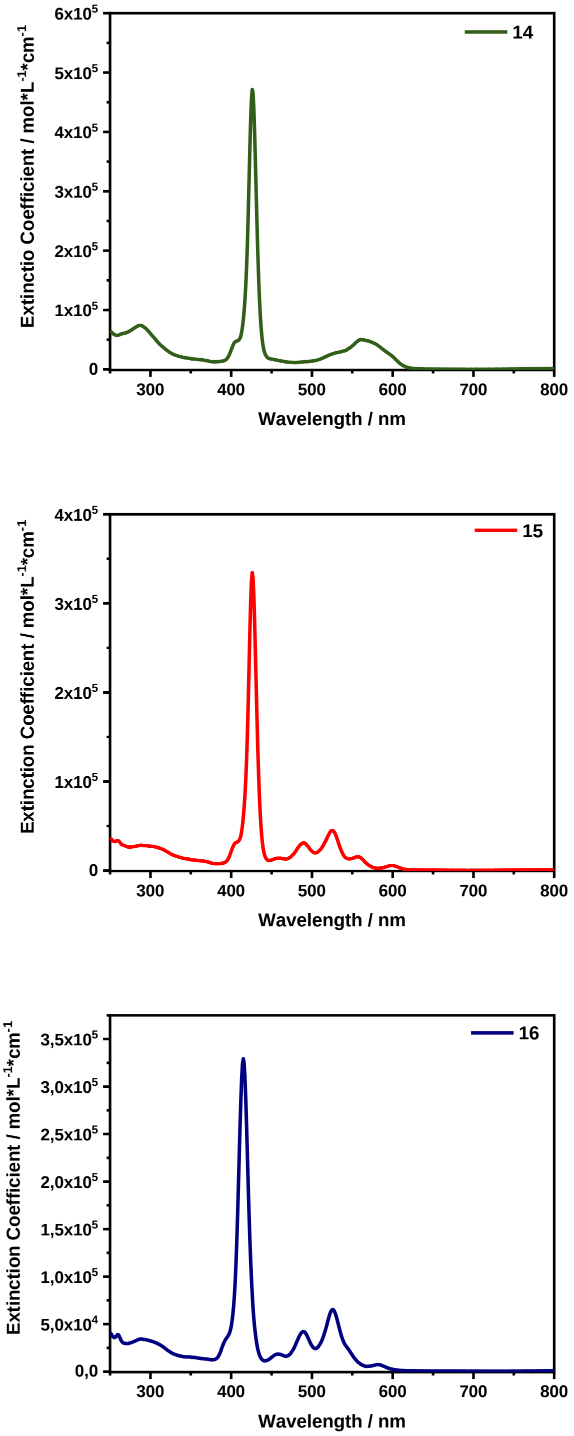

Figure S52 Molar extinction coefficients of the amphiphiles **14**, **15**, and **16**, in a mixture of mixture of THF and 10 mM aqueous NaOH solution (1:1 v/v) at room temperature.

UV/Vis Absorption THF Addition Experiments

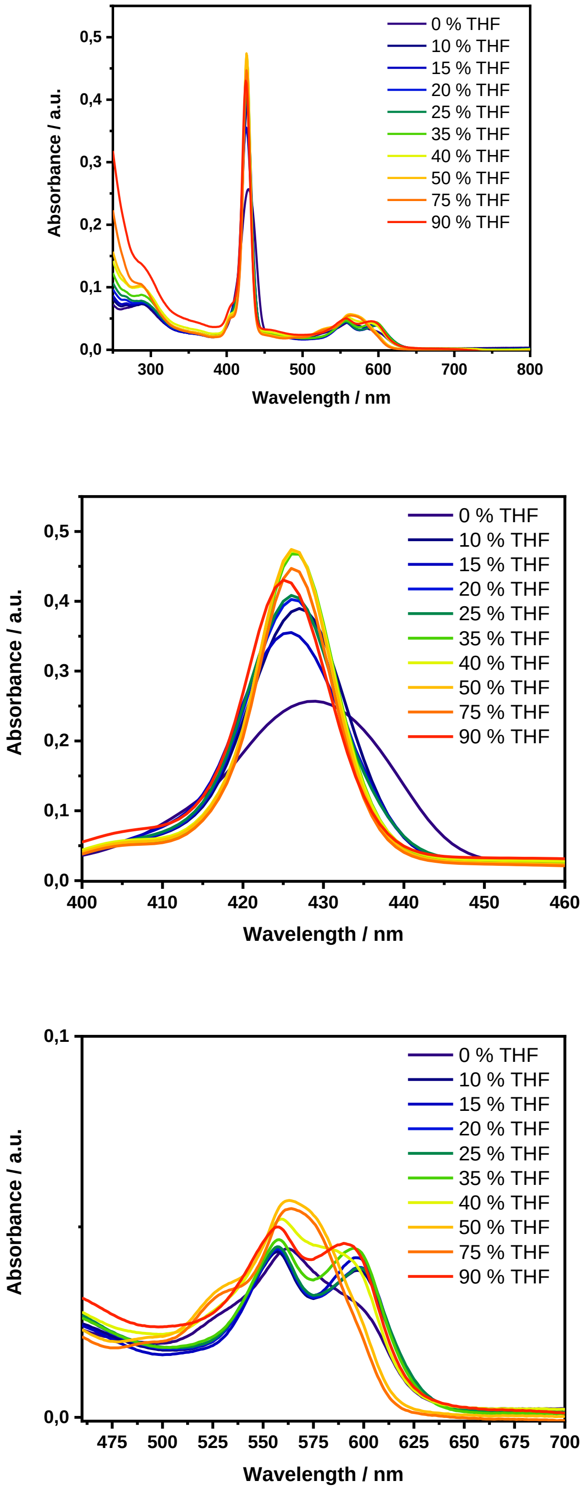

Figure S53 UV/Vis absorption spectra of **14** (c = 1.42*10^{-6} M) in 10 mM aqueous NaOH with different parts of THF as indicated. Higher parts of THF as 50 vol% lead to a re-aggregation of the samples, as the deprotonated dyads are not soluble in THF. Higher parts as 90 vol% THF lead to a precipitation of the compound.

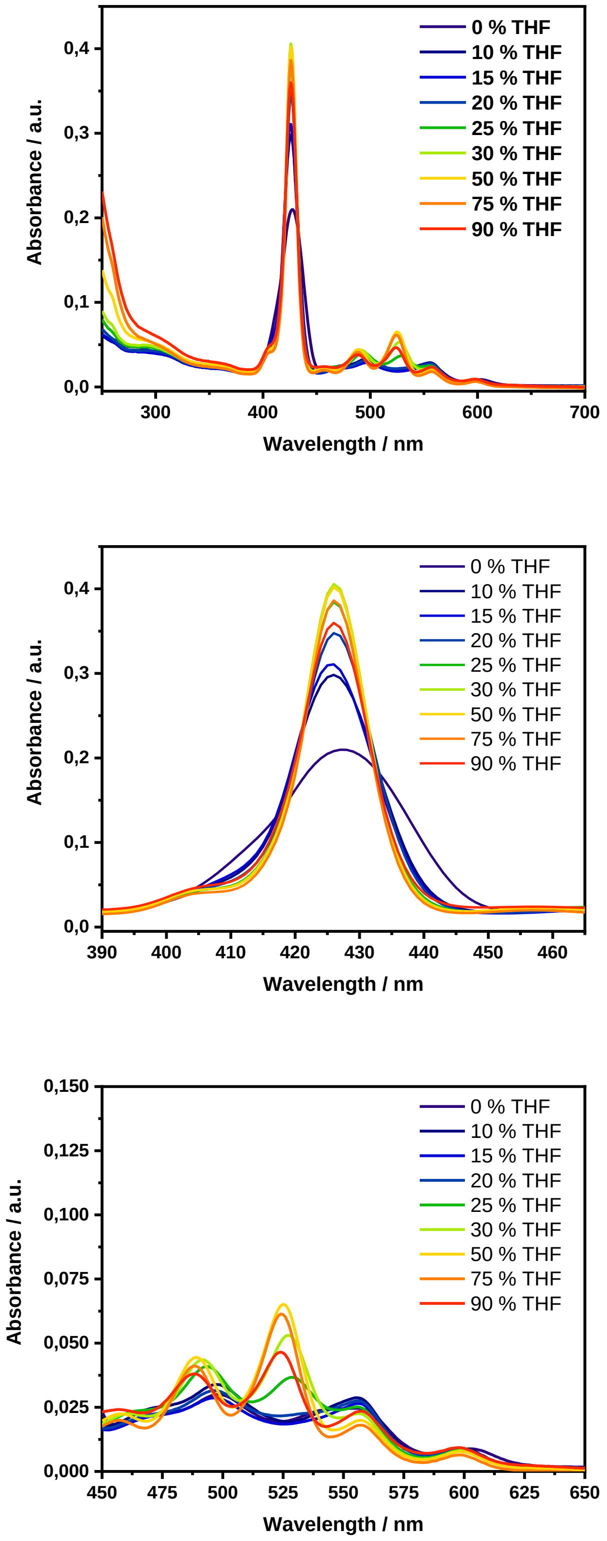

Figure S54 UV/Vis absorption spectra of **15** (c = 1.39*10^{-6} M) in 10 mM aqueous NaOH with different parts of THF as indicated. Higher parts of THF as 50 vol% lead to a re-aggregation of the samples, as the deprotonated dyads are not soluble in THF. Higher parts as 90 vol% THF lead to a precipitation of the compound.

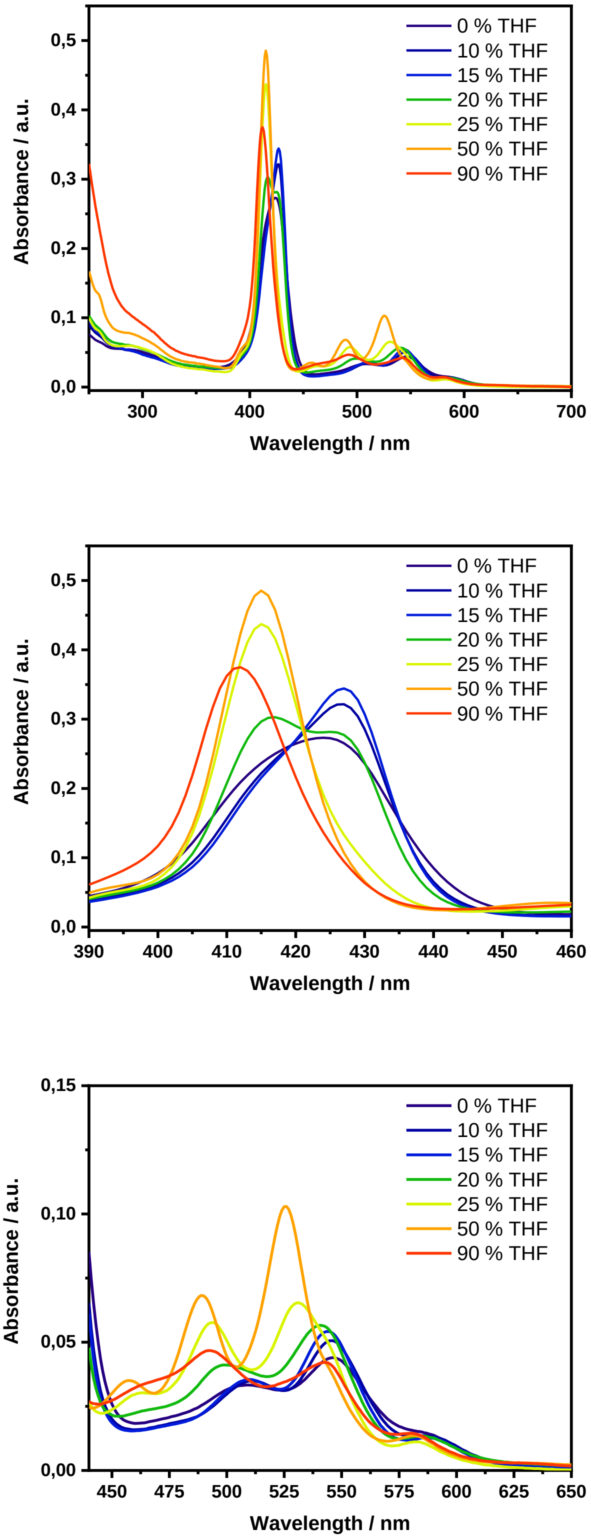

Figure S55 UV/Vis absorption spectra of **16** (c = 1.38*10^{-6} M) in 10 mM aqueous NaOH with different parts of THF as indicated. Higher parts of THF as 50 vol% lead to a re-aggregation of the samples, as the deprotonated dyads are not soluble in THF. Higher parts as 90 vol% THF lead to a precipitation of the compound.

Fluorescence emission THF addition experiments

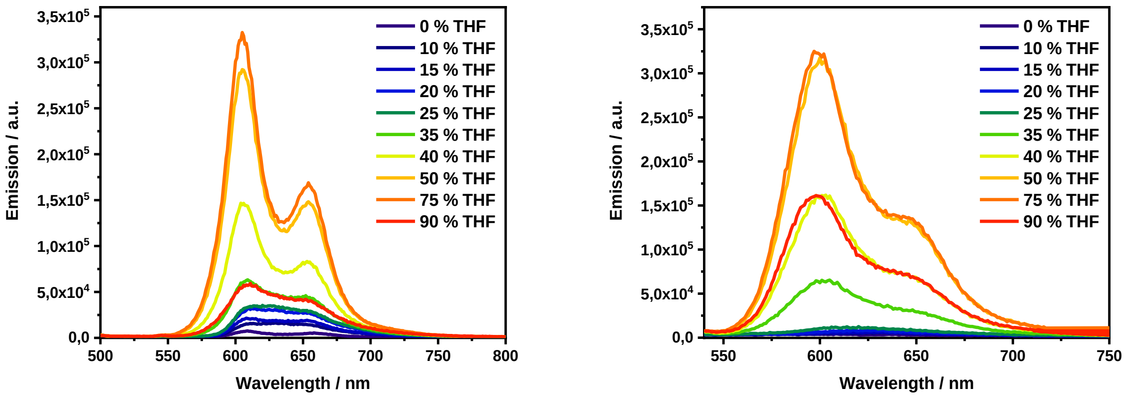

Figure S56 Left: Fluorescence emission spectrum of **14** (c = 1.42*10^{-6} M) excited at 425 nm; right: excited at 525 nm

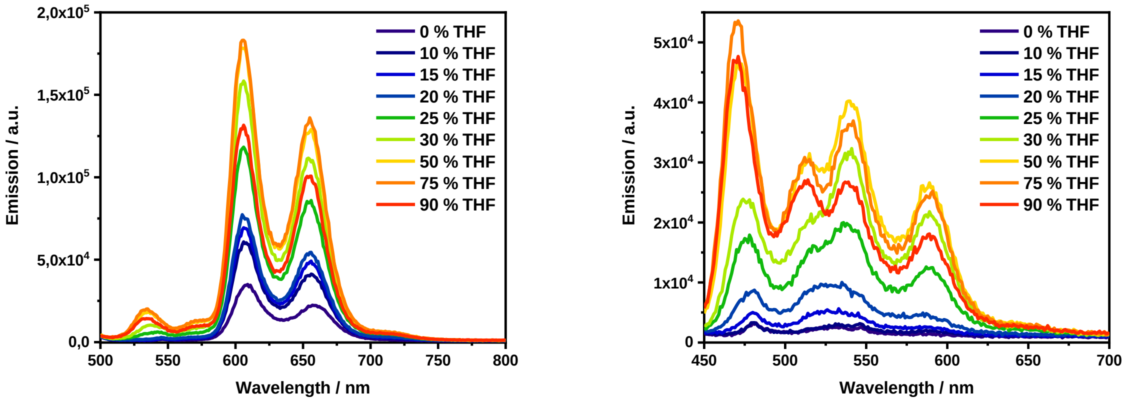

Figure S57 Left: Fluorescence emission spectrum of **15** (c = 1.39*10^{-6} M) excited at 425 nm; right: excited at 495 nm

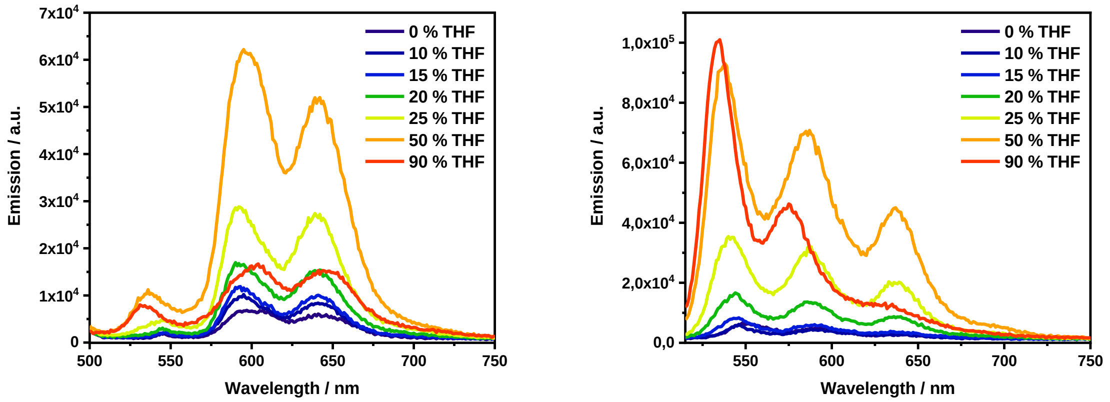

Figure S58 Left: Fluorescence emission spectrum of **15** (c = 1.39*10^{-6} M) excited at 425 nm; right: excited at 495 nm

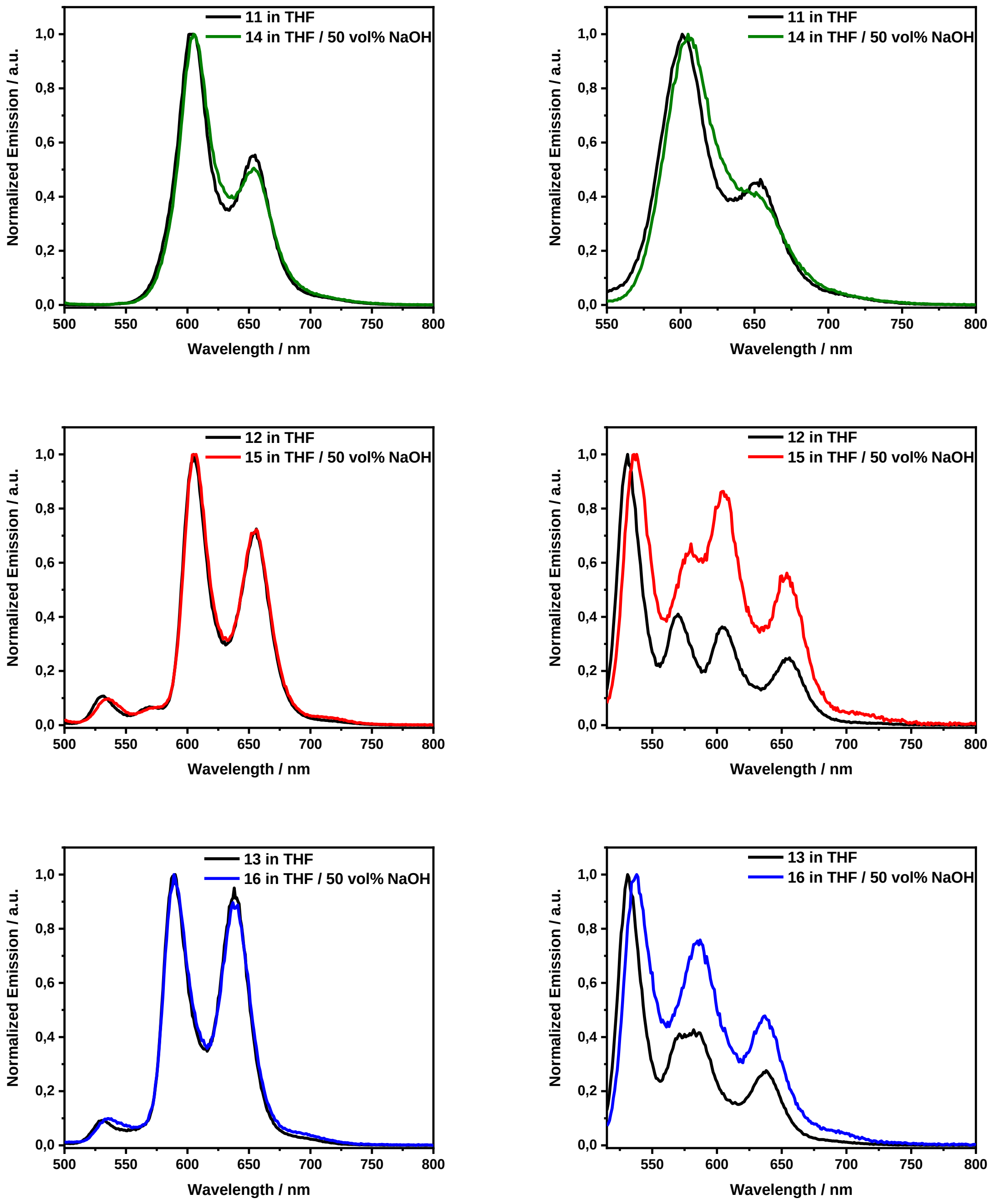

Figure S59 Normalized emission spectra of the tert-butyl ester dyads **11**, **12**, **13** and their respective amphiphilic dyads **14**, **15**, **16** in the indicated solvent mixture at room temperature. Left: excitation at the Soret-band; Right: excitation at the PBI.

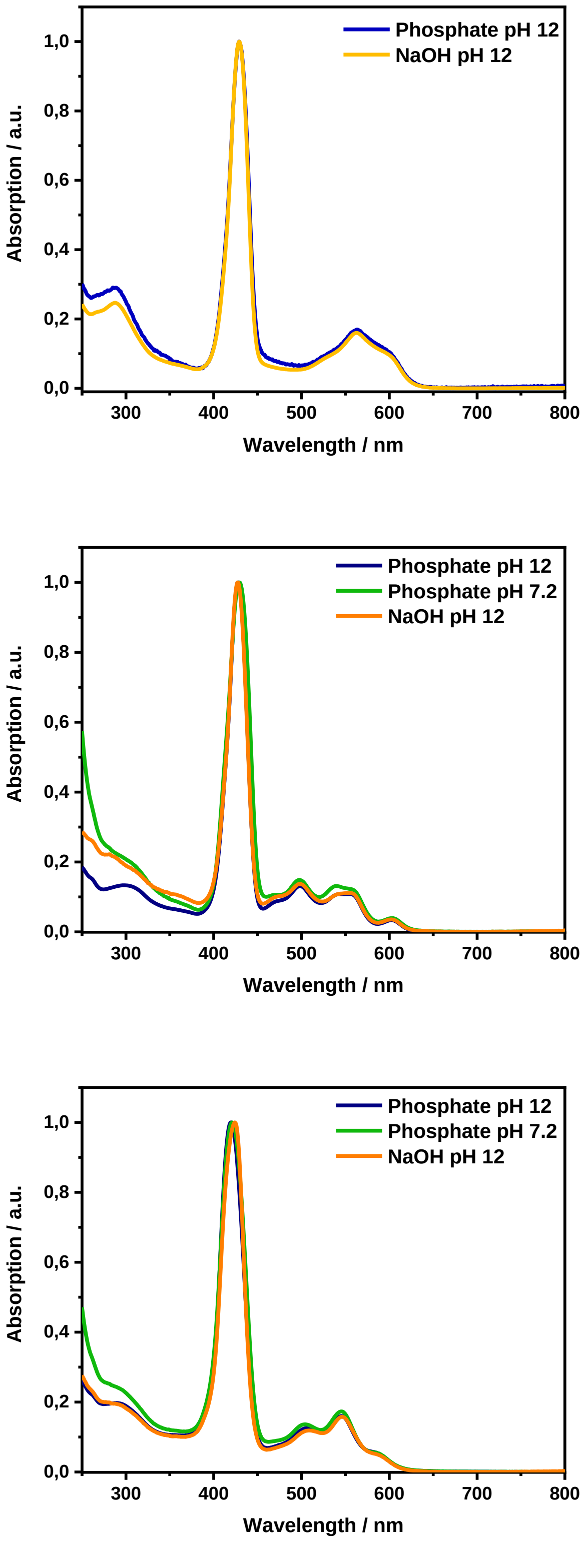

Figure S60 Absorption spectra of amphiphile **14** (top), **15** (middle), and **16** (bottom), in the indicated solvents (**14** was not soluble in the 7.2 pH phosphate buffered solution)

Time Dependent UV/Vis Absorption Experiments

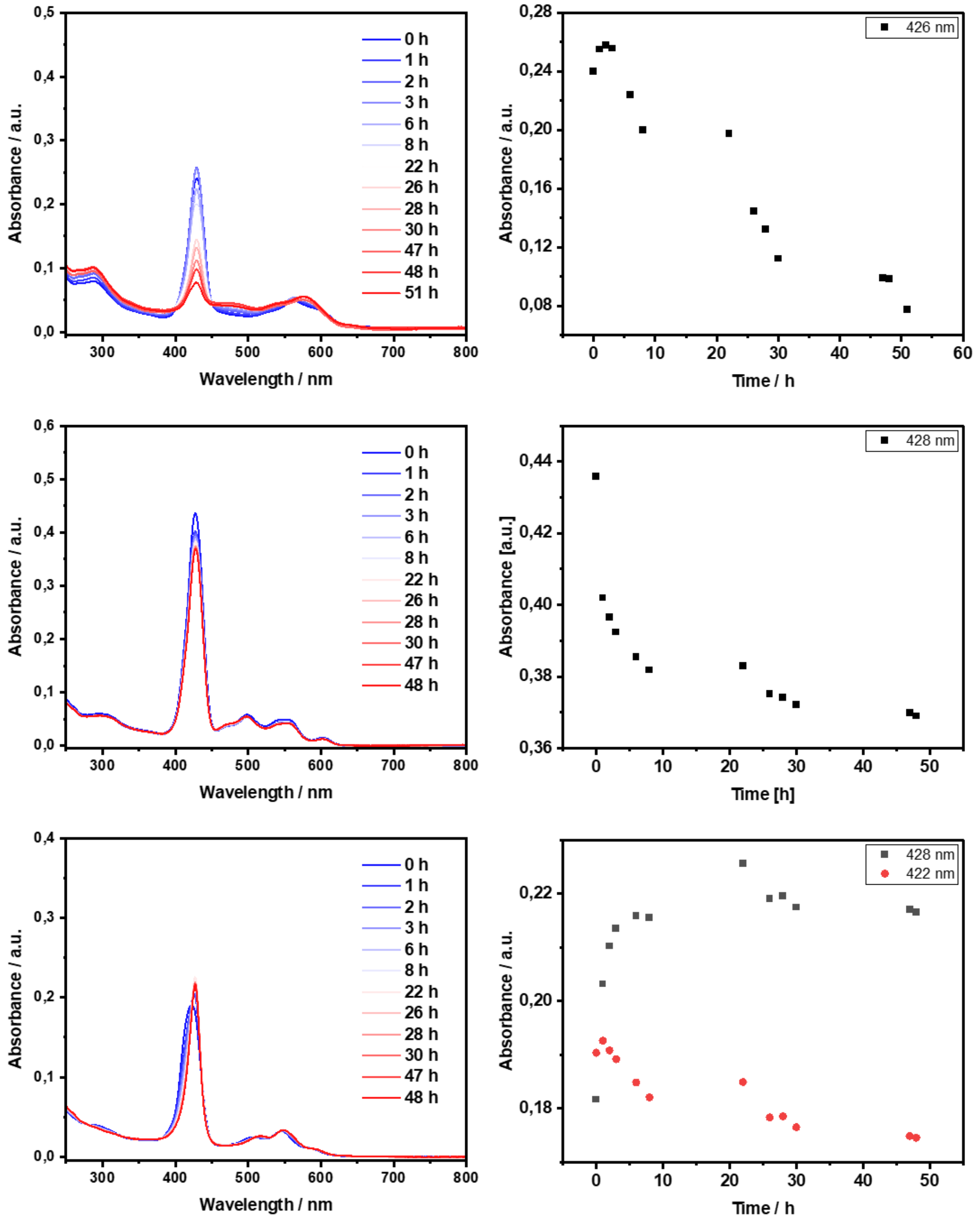

Figure S61 Left: time dependent absorption spectra of solutions of amphiphiles **14** (top; c=1.31*10^{-6}), **15** (middle; c=2.85*10^{-6}) and **16** (bottom; c=9.71*10^{-7}) in 10 mM NaOH solution; right: changes of the respective Soret-band absorbance over time.

UV/Vis Absorption THF Addition Experiments (Aged Solutions)

Denaturation experiments were carried out of the aged solutions in the same fashion as the fresh solutions. It has to be noted that the solutions for ageing and for denaturing stem from the same stock solution. However, since the ageing process is also dependent on the concentration, the starting point of the individualization experiment and the ageing endpoint are not the same.

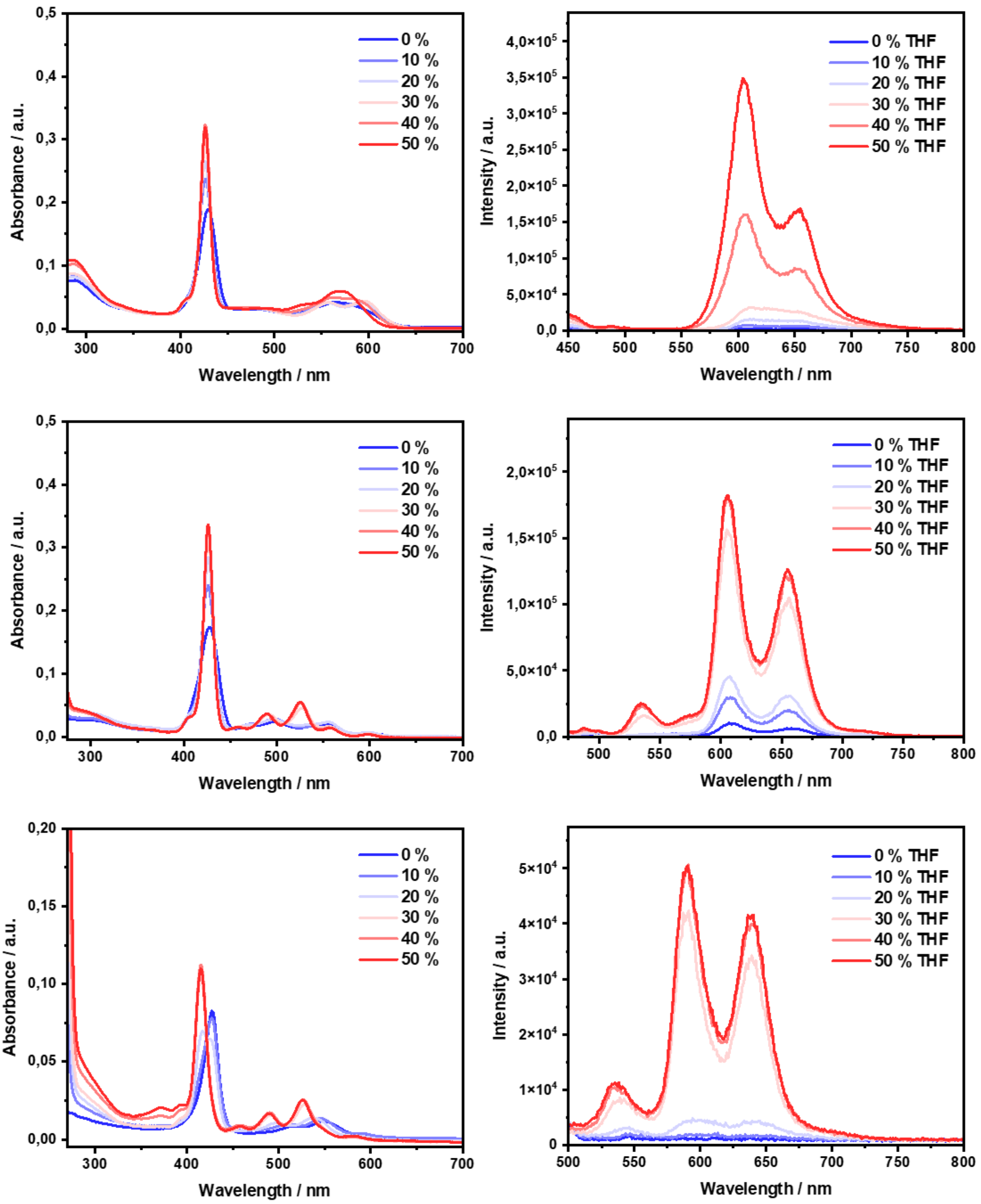

Figure S62 Left: absorption spectra of aged solutions of amphiphiles **14** (top; c=1.31*10^{-6}), **15** (middle; c=2.85*10^{-6}) and **16** (bottom; c=9.71*10^{-7}) in 10 mM NaOH solution containing different parts of THF; right: corresponding fluorescence emission spectra excited at 425 nm (**14** / **15**) and the 425 nm – 415 nm (**16**) following the Soret band

6. Theoretical Calculations

DFT calculation (geometry optimization, B3LYP, 631Gs) were performed by the SPARTAN '16 MECHANICS PROGRAM (Win/64b) Release 2.0.0[6]

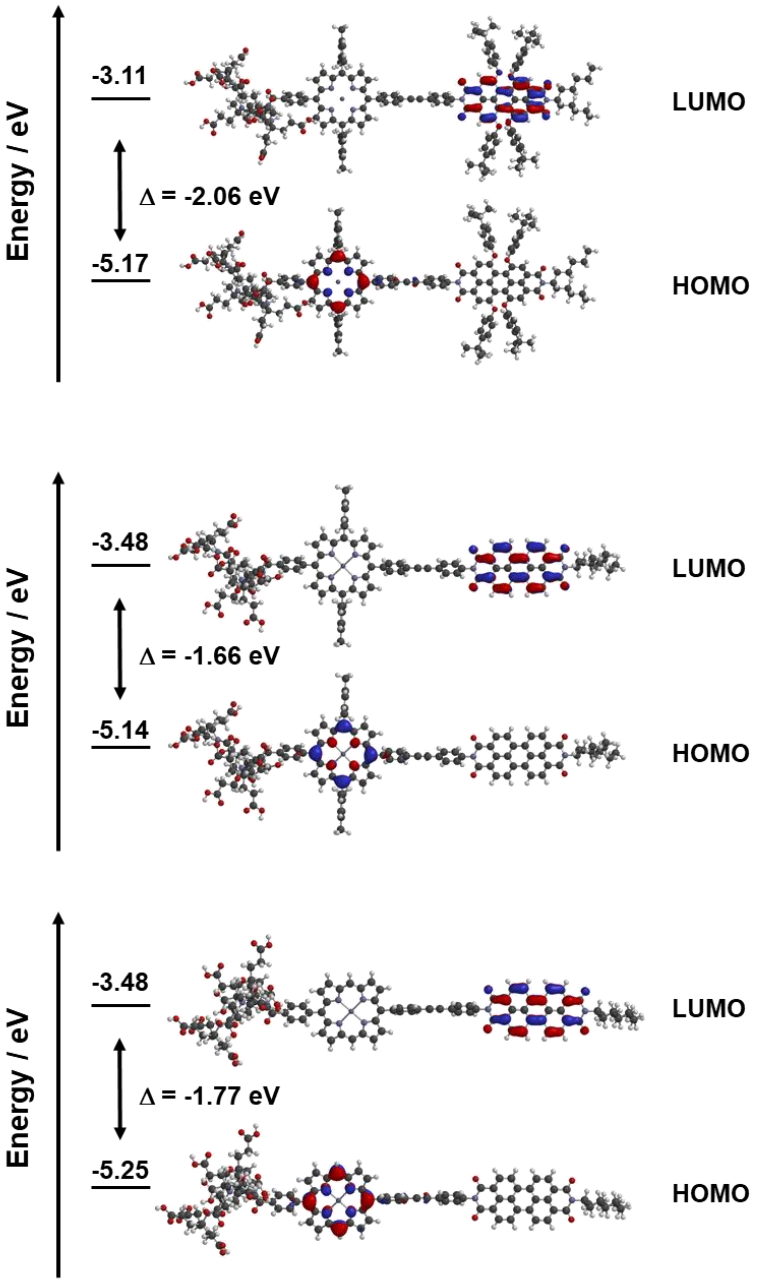

Figure S63 Geometry optimized structures of the amphiphiles **14**, **15**, and **16,** at the DFT B3LYP 6-31G* level of theory; orbitals are visualized at an iso-value = 0.03 with their HOMO-LUMO levels

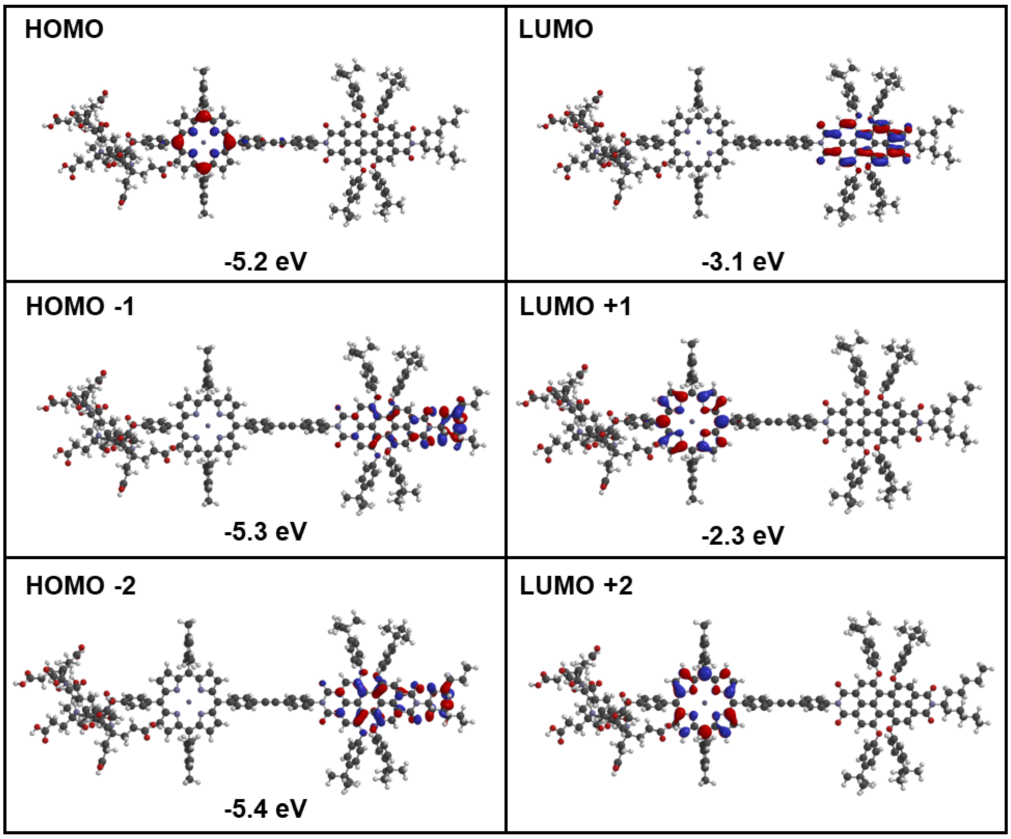

Figure S64 Geometry optimized structures of the amphiphile **14** at the DFT B3LYP 6-31G* level of theory; orbitals are visualized at an iso-value = 0.03

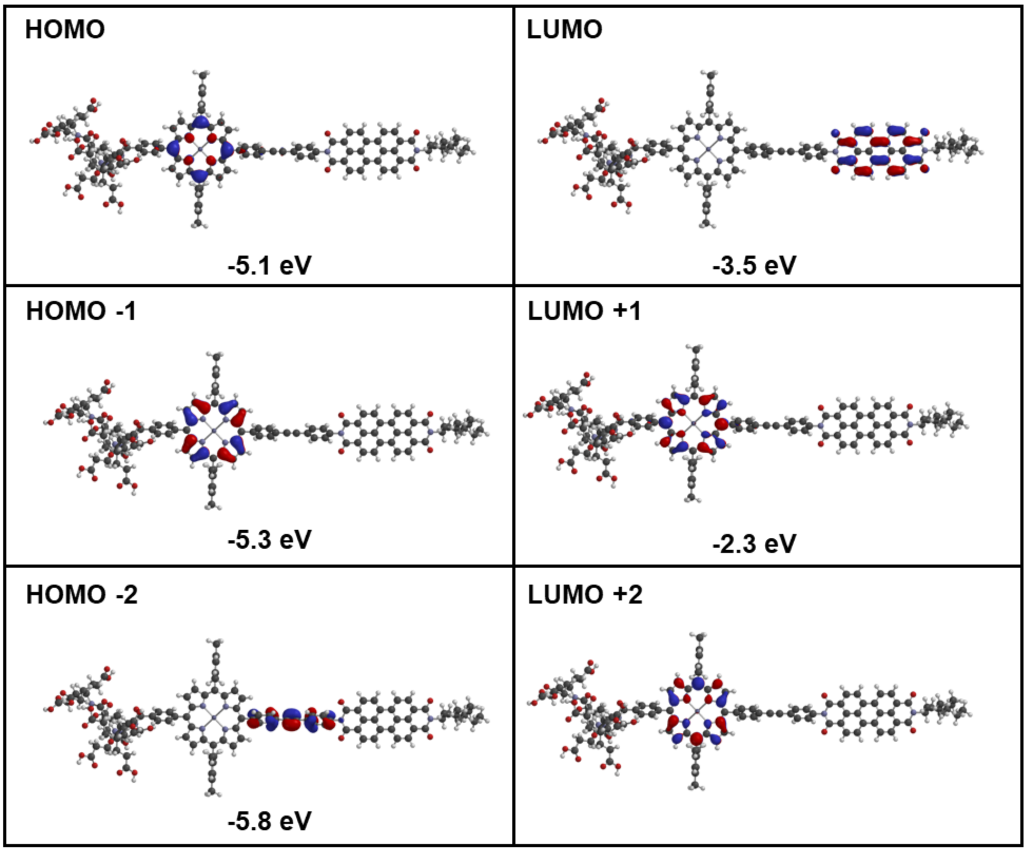

Figure S65 Geometry optimized structures of the amphiphile **15** at the DFT B3LYP 6-31G* level of theory; orbitals are visualized at an iso-value = 0.03

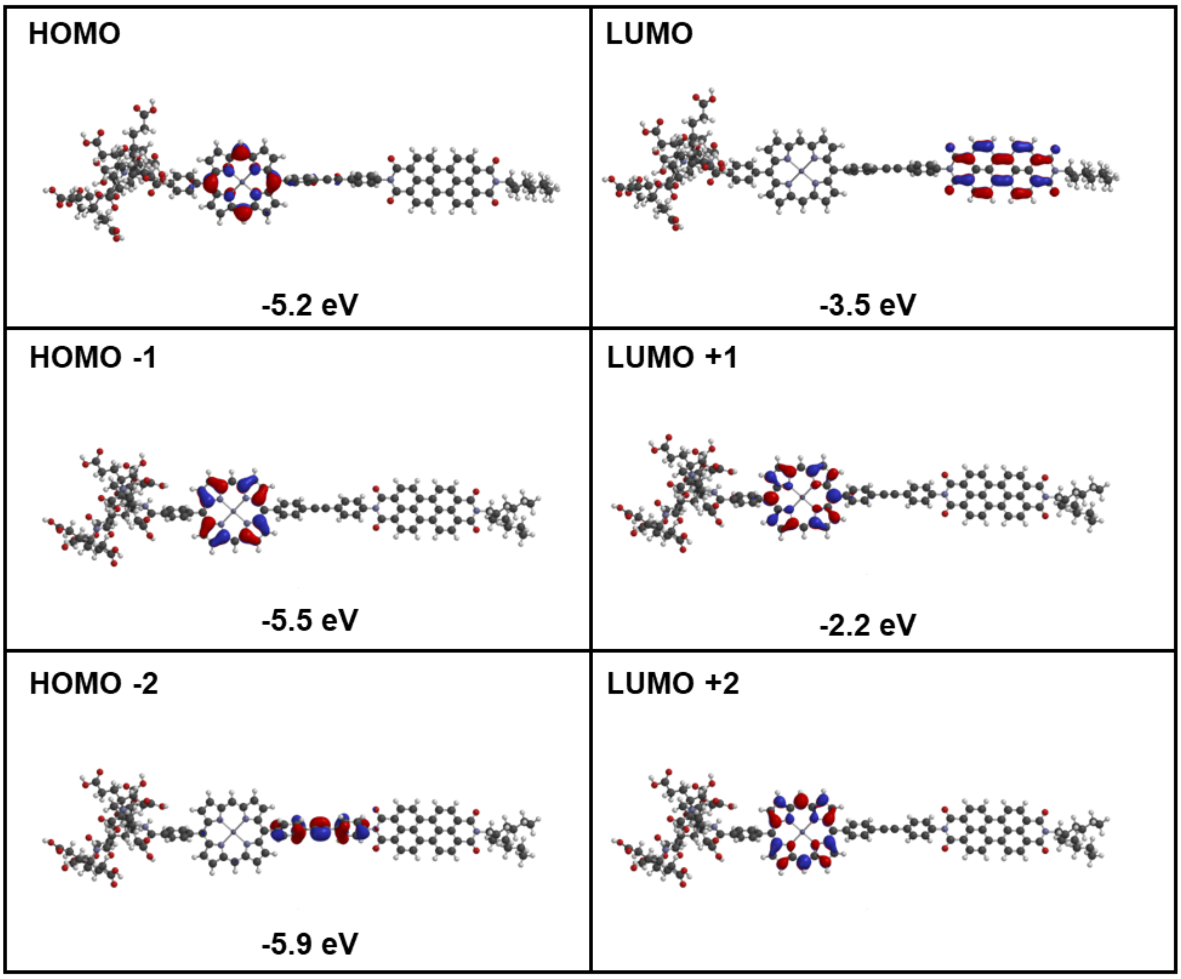

Figure S66 Geometry optimized structures of the amphiphile **16** at the DFT B3LYP 6-31G* level of theory; orbitals are visualized at an iso-value = 0.03

7. Dynamic Light Scattering (DLS)

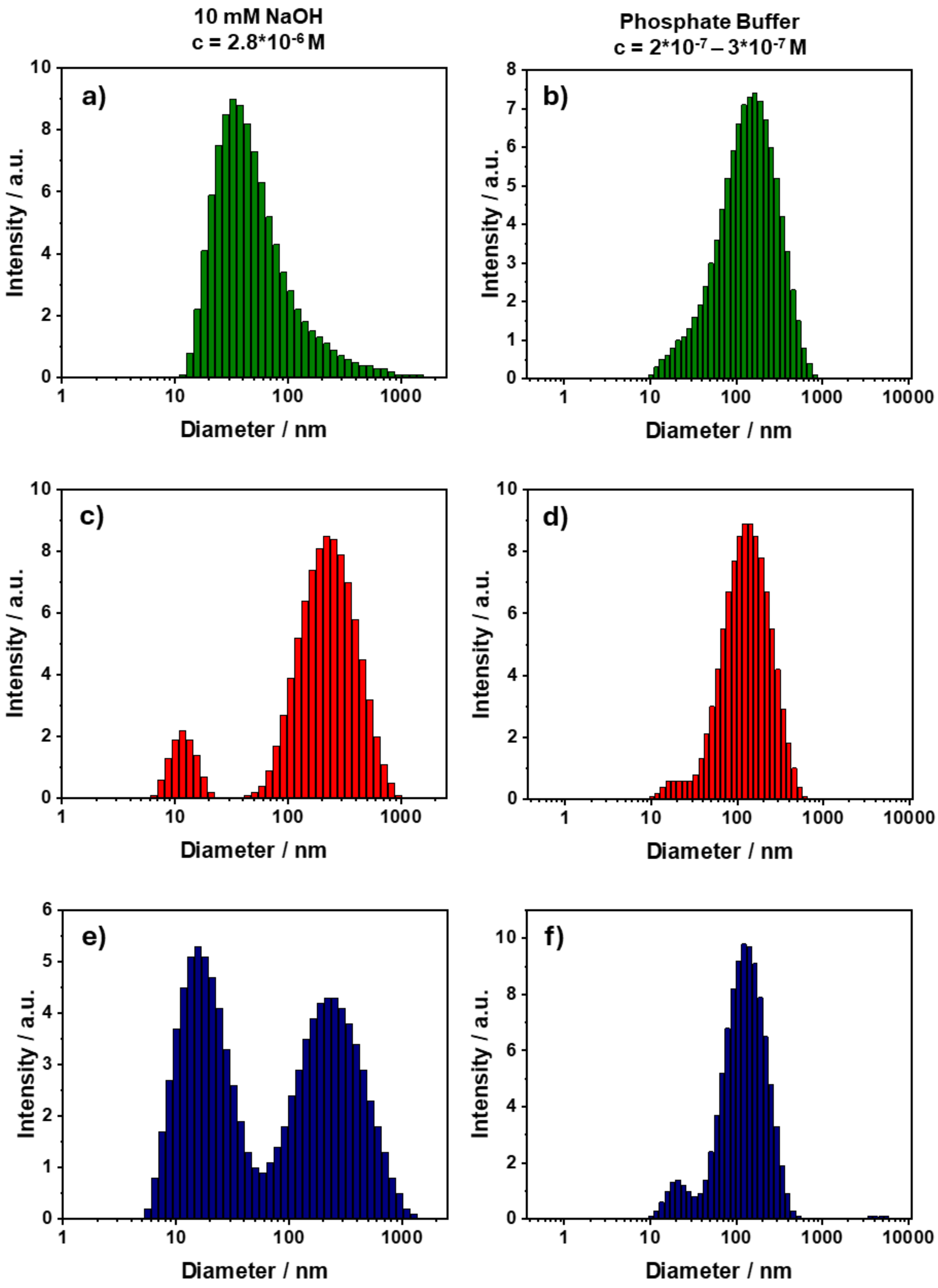

Figure S67 Left: DLS size distributions (intensity) of **14** (c = 2.8*10^{-6} M) b) **15** (c = 2.8*10^{-6} M) c) **16** (c = 2.8*10^{-6} M) in 10 mM aqueous NaOH (approx. pH 10); Right: DLS size distributions (intensity) of **14** (c = 2.6*10^{-7} M) b) **15** (c = 4.2*10^{-7} M) c) **16** (c = 1.9*10^{-7} M) in phosphate buffered solution (pH 12);

8. IR-Spectra

Protected Dyads

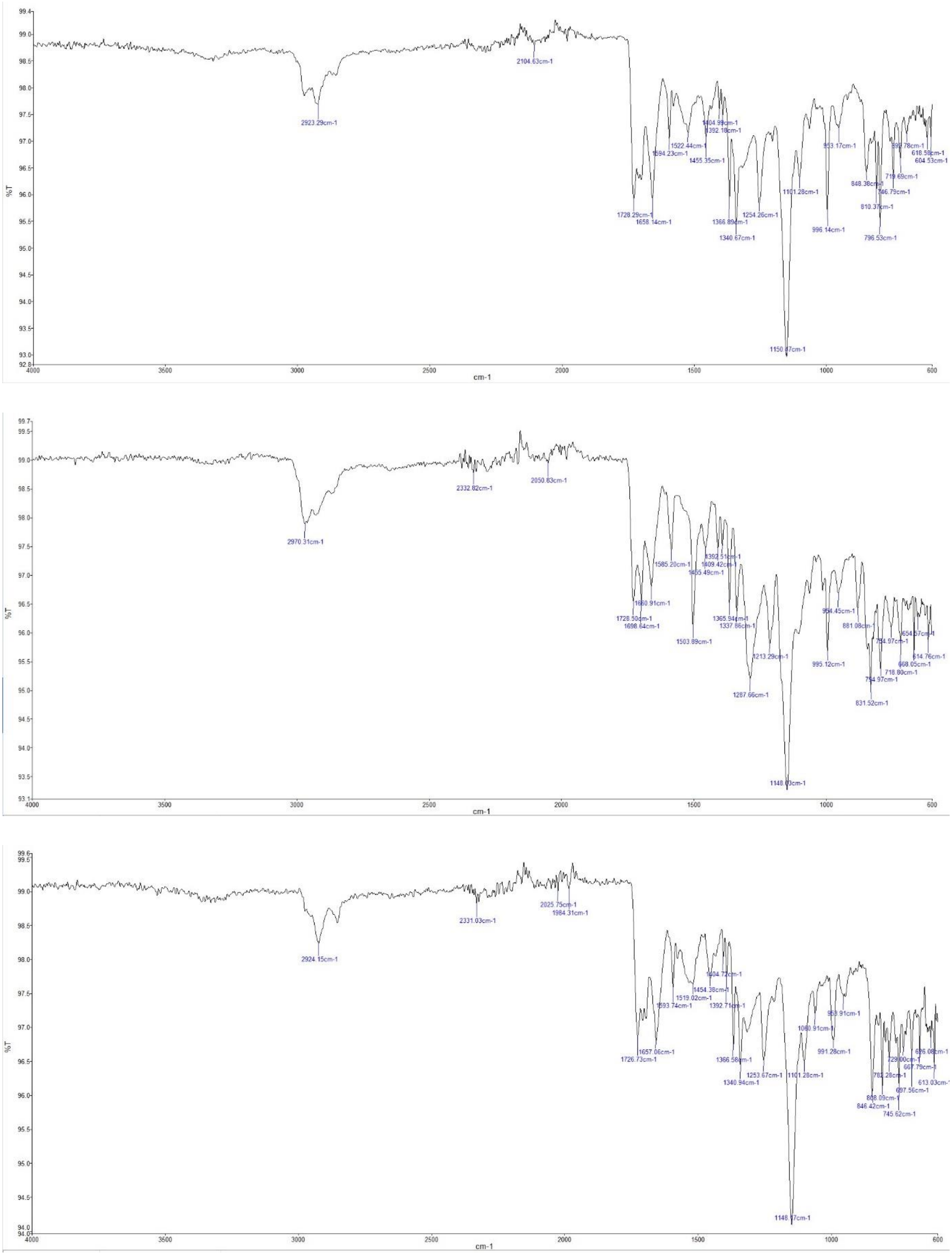

Figure S68 ATR-IR spectra of the tert-Butyl ester dyads, top: **11**, middle: **12**, bottom: **13**

Amphiphilic Dyads

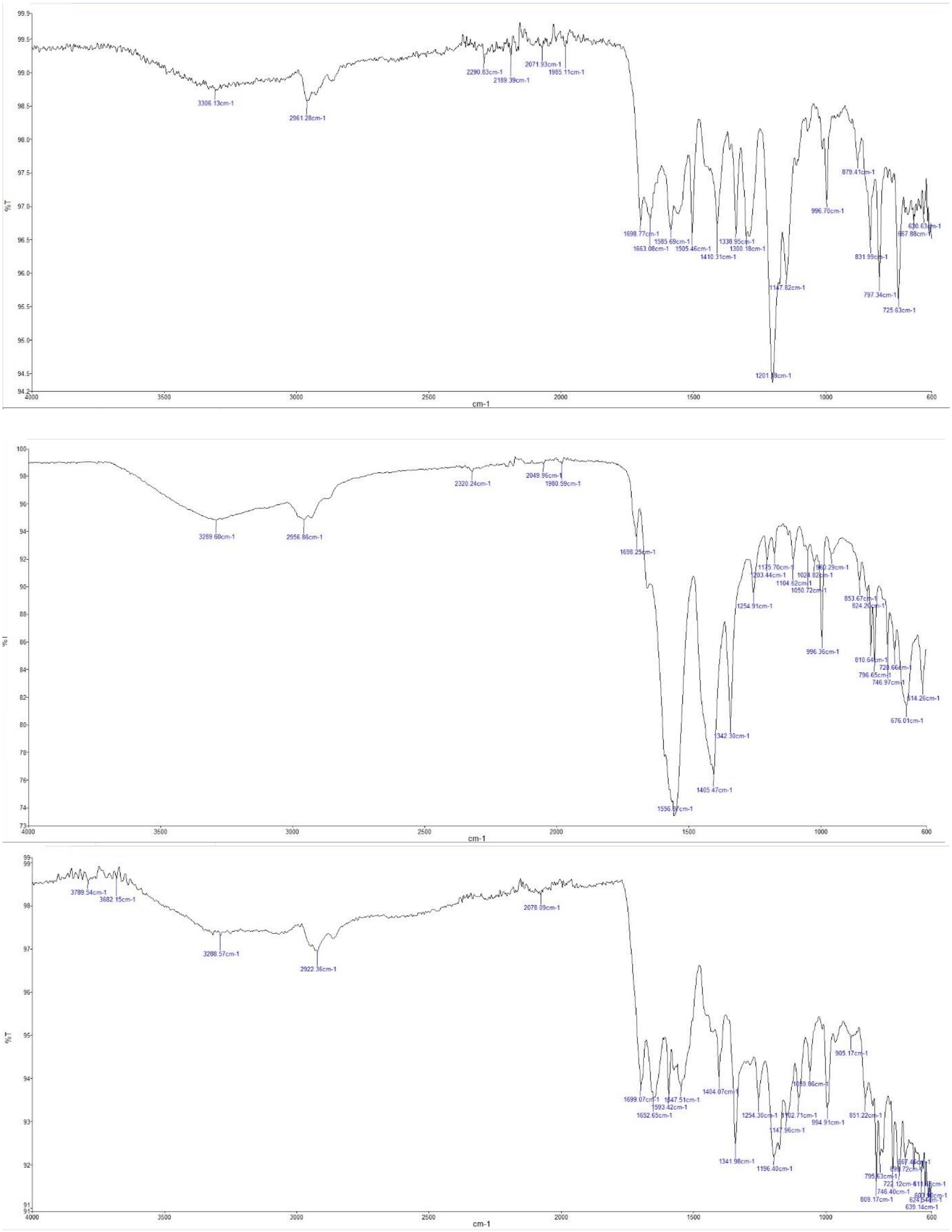

Figure S69 ATR-IR spectra of the tert-Butyl ester dyads, top: **14**, middle: **15**, bottom: **16**

9. STEM Imaging

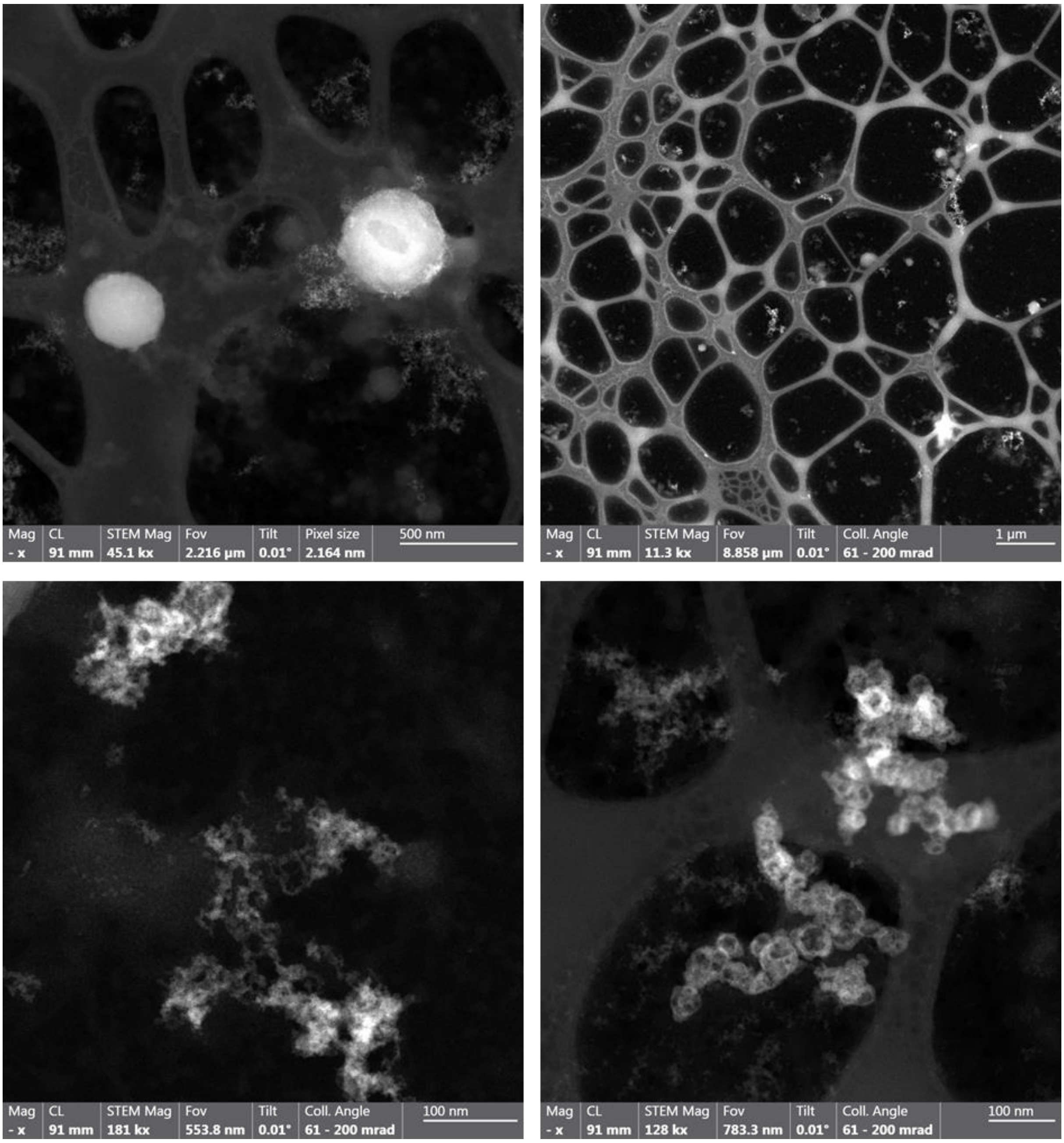

Figure S70 STEM images of the sodium salt of **14** (1.0 mM aqueous solution)

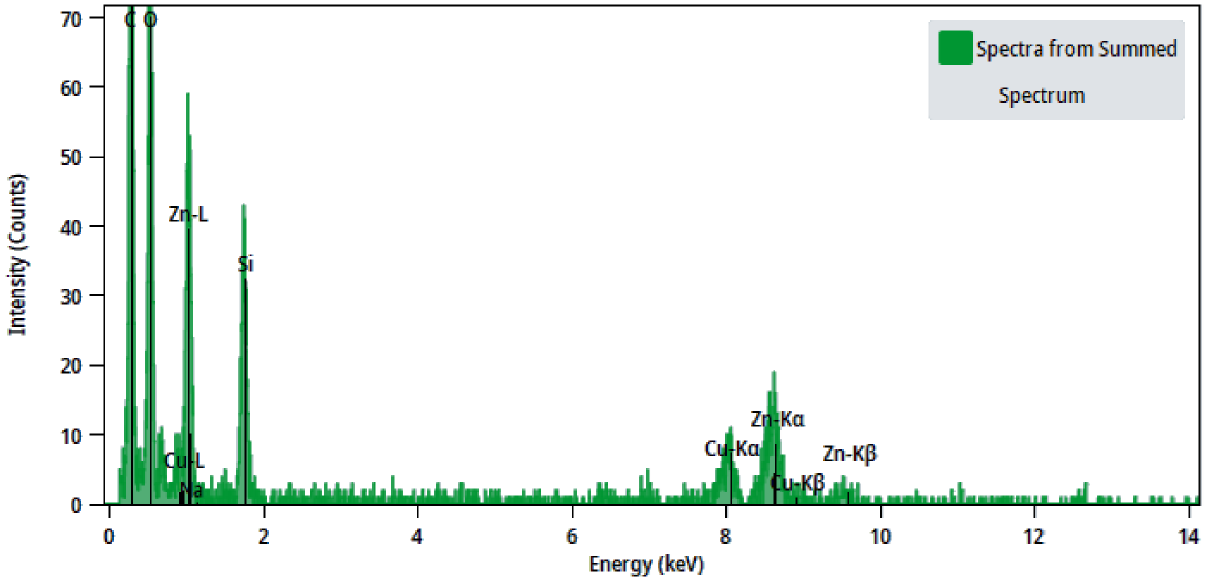

Figure S71 EDXS images of 14. These reveal the Zn signal which is present in the spheres. (Cu and Si are known artifact from the instrument setup)

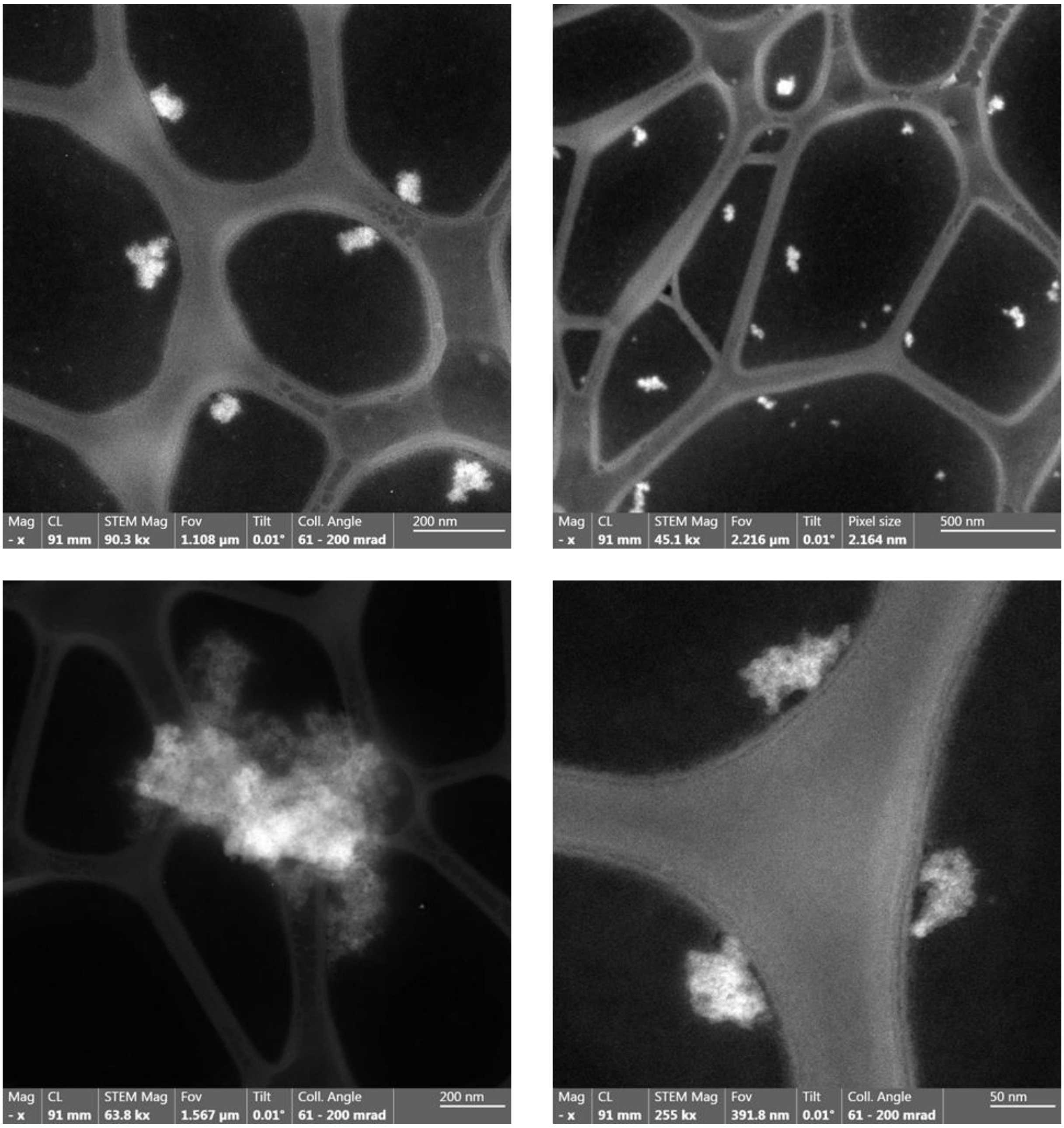

Figure S72 STEM images of the sodium salt of **15** (2.0 mM aqueous solution)

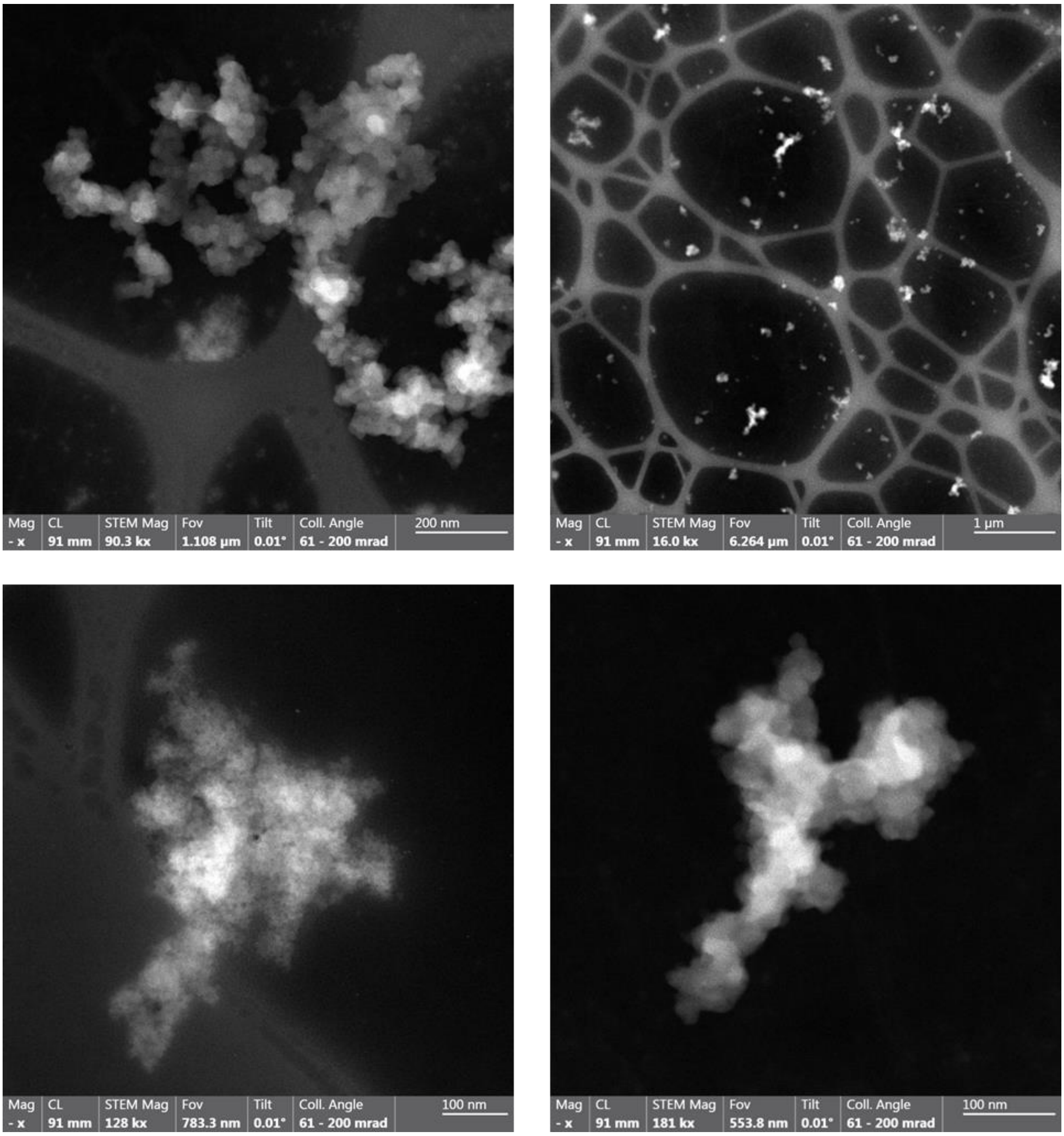

Figure S73 STEM images of the sodium salt of **16** (2.0 mM aqueous solution)

10. References

[1] E. J. Schulze, C. L. Ritterhoff, E. Franz, O. Tavlui, O. Brummel, B. Meyer, A. Hirsch, *Chem. Eur. J.* **2024**, *30*, e202303515.

[2] C. Oleszak, C. L. Ritterhoff, E. J. Schulze, A. Hirsch, B. Meyer, N. Jux, *RSC Adv* **2025**, *15*, 1212–1219.

[3] J. J. Lee, S. C. Lee, D. Zhai, Y. H. Ahn, H. Y. Yeo, Y. L. Tan, Y. T. Chang, *Chem. Commun.* **2011**, *47*, 4508–4510.

[4] W. S. Cho, H. J. Kim, B. J. Littler, M. A. Miller, C. H. Lee, J. S. Lindsey, *J. Org. Chem.* **1999**, *64*, 7890–7901.

[5] G. Hu, H. S. Kang, A. K. Mandal, A. Roy, C. Kirmaier, D. F. Bocian, D. Holten, J. S. Lindsey, *RSC Adv.* **2018**, *8*, 23854–23874.

[6] Y. Shao, L. F. Molnar, Y. Jung, J. Kussmann, C. Ochsenfeld, S. T. Brown, A. T. B. Gilbert, L. V Slipchenko, S. V Levchenko, D. P. O'Neill, R. A. DiStasio, R. C. Lochan, T. Wang, G. J. O. Beran, N. A. Besley, J. M. Herbert, C. Yeh Lin, T. Van Voorhis, S. Hung Chien, A. Sodt, R. P. Steele, V. A. Rassolov, P. E. Maslen, P. P. Korambath, R. D. Adamson, B. Austin, J. Baker, E. F. C. Byrd, H. Dachsel, R. J. Doerksen, A. Dreuw, B. D. Dunietz, A. D. Dutoi, T. R. Furlani, S. R. Gwaltney, A. Heyden, S. Hirata, C. P. Hsu, G. Kedziora, R. Z. Khalliulin, P. Klunzinger, A. M. Lee, M. S. Lee, W. Z. Liang, I. Lotan, N. Nair, B. Peters, E. I. Proynov, P. A. Pieniazek, Y. Min Rhee, J. Ritchie, E. Rosta, C. David Sherrill, A. C. Simmonett, J. E. Subotnik, H. Lee Woodcock, W. Zhang, A. T. Bell, A. K. Chakraborty, D. M. Chipman, F. J. Keil, A. Warshel, W. J. Hehre, H. F. Schaefer, J. Kong, A. I. Krylov, P. M. W. Gill, M. Head-Gordon, *Phys. Chem. Chem. Phys.* **2006**, *8*, 3172–3191.
